# Targeting Urease: A Promising Adjuvant Strategy for Effective *Helicobacter pylori* Eradication

**DOI:** 10.1021/acsomega.5c02725

**Published:** 2025-07-04

**Authors:** Shivani Kunkalienkar, Neha S. Gandhi, Ashutosh Gupta, Moumita Saha, Aravinda Pai, Shiran Shetty, Abhishek Gupta, Namdev Dhas, Raghu Chandrashekar Hariharapura, K. Nandakumar, Nagalakshmi Narasimhaswamy, Sudheer Moorkoth

**Affiliations:** † Department of Pharmaceutical Quality Assurance, Manipal College of Pharmaceutical Sciences, 76793 Manipal Academy of Higher Education, Manipal 576 104, Karnataka, India; ‡ Department of Biotechnology, Manipal Institute of Technology, Manipal Academy of Higher Education, Manipal 576 104, India; § Department of Pharmaceutical Chemistry, Manipal College of Pharmaceutical Sciences, Manipal Academy of Higher Education, Manipal 576 104, Karnataka, India; ∥ Department of Gastroenterology and Hepatology, Kasturba Medical College, Manipal Academy of Higher Education, Manipal 576 104, Karnataka, India; ⊥ School of Pharmacy and Life Sciences, Faculty of Science and Engineering, 8695 University of Wolverhampton, Wulfruna Street, Wolverhampton WV1 1LY, U.K.; # Department of Pharmaceutics, Manipal College of Pharmaceutical Sciences, Manipal Academy of Higher Education, Manipal 576 104, Karnataka, India; ¶ Department of Pharmaceutical Biotechnology, Manipal College of Pharmaceutical Sciences, Manipal Academy of Higher Education, Manipal 576 104, Karnataka, India; ∇ Department of Pharmacology, Manipal College of Pharmaceutical Sciences, Manipal Academy of Higher Education, Manipal 576 104, Karnataka, India; ○ Department of Microbiology, Faculty of Medicine, Manipal University College Malaysia, Melaka 75150, Malaysia

## Abstract

*Helicobacter pylori*, a Gram-negative bacterium, exhibits unique adaptations to thrive in an acidic gastric environment. Urease enzyme present in the bacteria converts urea to ammonia and carbon dioxide, making the surrounding acidic environment of the bacteria neutral. This adaptation helps the bacteria to survive and travel further to gastric epithelial cells, where it attaches to mucin and damages the tissues, leading to gastritis, peptic ulcer, and ultimately, cancer. Physicians typically prescribe first-, second-, and third-line antibiotic therapies to eliminate the bacterium, but these treatments frequently fail to achieve complete eradication. This failure, driven by factors such as the coccoid form, high bacterial load, and biofilm formation, contributes to the growing problem of antibiotic resistance. Targeting urease activity presents a promising strategy to reduce the *H. pylori* pathogenicity and enhance its susceptibility to antibiotics. Inhibiting urease enzyme activity would be an option to make the bacteria less pathogenic and more prone to antibiotic treatment. Including the urease inhibitors as an adjuvant with the current antibiotic treatment regimen would effectively eradicate the bacteria. This comprehensive review discusses the structural characteristics of the urease enzyme and its role in pathogenesis and the available urease inhibitors along with their pharmacophoric features. An elaborative pharmacophore-based screening and docking study on scaffolds such as chlorogenic acid, catechol, and hydroxamic acid to discover a potent urease inhibitor is a future scope identified in this review.

## Graphical abstract

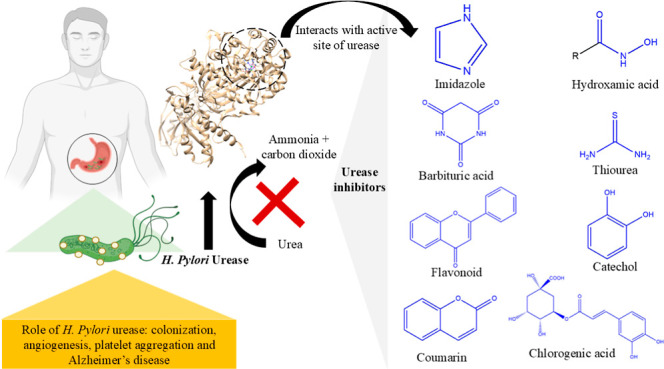

## 1 Introduction

*Helicobacter pylori* (*H. pylori*) is a rod-shaped, multiflagellate, microaerophilic, and Gram-negative bacteria infecting greater than half of the world’s population.^1,2^ The bacterium was first recognized as a significant cause of gastric ulcers in 1982 by two Australian physicians, Barry Marshall and Robin Warren. Their groundbreaking discovery led to their recognition with the Noble Prize in Physiology or Medicine in 2005, highlighting the association of *H. pylori* in gastrointestinal health.^1^ The bacterium causes diseases like chronic gastritis, peptic ulcer disease, MALT lymphoma, nonulcer dyspepsia, inflammatory bowel disease, gastroesophageal reflux disease, diffuse duodenal nodular lymphoid hyperplasia, antral polyp, and gastric cancer.^3–11^ *H. pylori* can be transmitted through oral–oral, fecal–oral, or sexual route.^12^ Several risk factors are associated with infection by *H. pylori*, including, but not limited to, lower socio-economic status (SES), overcrowded living conditions, inadequate sanitation and hygiene, close contact with infected individuals, consumption of contaminated food or water, smoking, alcohol use, dental conditions such as dental caries, and certain dietary habits like consumption of undercooked meat and spicy foods.^13–20^

In addition to SES, the prevalence of *H. pylori* varies with gender, age, and geographic location.^21^ The infection is most widespread in Africa, where prevalence rates often reach or exceed 70%. In West Africa, Nigeria has reported a wide range of prevalence ranging from 38% to as high as 92%, with children being the most affected group. Within Nigeria, the northern region reported a prevalence of 87.8%, while the southern region showed rates ranging from 28% to 51.4%. Other West African countries, such as Togo (93.1%), Cameroon (73.2%), the Republic of Benin (71.5%), and Congo-Brazzaville (70.41%) also reported high prevalence rates. In countries like Côte d’Ivoire, Ghana, and Senegal, the *H. pylori* infection rate was approximately 50%. In East Africa, Burundi and Rwanda had a prevalence of 70.8% and 75% respectively.^22^ Outside Africa, high seroprevalence rates were observed in Bangladesh (92%), India (79%), and Vietnam (74.6%).^23^ Overall, developing countries have shown a higher prevalence of *H. pylori* infection (50.8%) compared to developed countries (34.7%).^24^

Age-related differences were also noted, with higher infection rates found in young adults (48.6%) compared to children (32.6%).^24^ India has a large population, with more than 70% of its people under the age of 35 years.^25^ As a result, the country is particularly vulnerable to the burden of *H. pylori* infection. This public health challenge could have a substantial impact on national development and progress.

Treating *H. pylori* is very crucial for preventing conditions like peptic ulcer and gastric cancer.^26^ Most clinical guidelines recommend triple therapy as the first-line treatment for management of *H. pylori*.^21,27–30^ This typically includes PPI and two antibiotics, commonly amoxicillin, clarithromycin, or metronidazole. Triple therapy with clarithromycin and nonbismuth and bismuth quadruple therapy are the most popular first-line of treatment. When first-line treatment fails, a second-line treatment regimen is often prescribed, which includes a levofloxacin-based triple therapy. This regimen showed an eradication rate of 69% in 7 days and 84% in 10 days of treatment. However, increased resistance to levofloxacin, particularly due to its frequent use to treat pneumonia, poses a clinical challenge.^31^ An alternative, second-line treatment option is a sequential therapy with moxifloxacin and levofloxacin.^31–33^ If both first- and second-line treatment fails, then physicians recommend antibiotic susceptibility testing since options become restricted. Third-line treatment includes the use of rifabutin, rifaximin, and sitafloxacin. These treatment regimens are very intensive yet unable to eradicate the organism completely, resulting in antibiotic resistance. Several factors lead to treatment failure, including the bacterium’s ability to survive in the acidic gastric environment leading to high bacterial load, biofilm formation, and the limited penetration of antibiotics into the deeper layers of the gastric epithelium where the bacteria reside.^34^

*H. pylori* has several unique adaptations to thrive in a harsh acidic environment and colonize in the stomach. The unique mechanisms including, but not limiting to, urease production, motility, and adhesion, help it to reside in the gastric environment.^1^ The urease found in the cytosol and on the surface of *H. pylori*, hydrolyses urea to ammonia and carbon dioxide.^1,35–38^ The resulting ammonia neutralizes the gastric acidic environment around the bacteria. Once *H. pylori* survives the acidic environment, it penetrates the basal layer of the stomach mucosal epithelial layer using its sheathed flagella, which facilitates motility through gastric mucosa. Here, the outer membrane proteins (OMP) of *H. pylori* serve as adhesins, allowing it to attach to the gastric mucosa, especially mucin (MUC5AC) of the stomach. Proteins such as sialic acid-binding adhesin, blood-antigen binding protein A (BabA), lacdiNAc-binding adhesin, neutrophil-activating protein, heat shock protein 60, outer membrane protein (HopZ), and adherence-associated proteins (AlpA and AlpB), play essential roles in bacterial adhesion and pathogenesis, such as inflammation and ulceration which may further lead to cancer.^39,40^ Considering these special adaptive features of the bacterium, one potential approach to prevent colonization could be by targeting *H. pylori* urease enzyme with specific urease inhibitors. This article explores the potential of urease inhibitors, as an adjuvant, with antibiotic therapy for eradication of the organism.

## 2 *H. pylori* Urease

### 2.1 *H. pylori* Urease Genes

*H. pylori* urease enzyme is a large assembly of structures with a molecular weight (300–550 kDa).^41,42^ The enzyme consists of seven gene clusters, which makes it active in carrying out urea hydrolysis (Table 1). These seven genes are present in chromosomes of *H. pylori* in a single 6.13 kb gene cluster, where all genes are transcribed in the same direction.^43^ It also has nickel ions, which is a metal cofactor.^39^ The supramolecular assembly of urease contributes to acid tolerance and *H. pylori*’s ability to endure in the gastric acidic environment.^39,44^

**Table 1:** Role of *H. pylori* Urease Genes

urease genes of *H. pylori*	role
Structural Genes	
UreA (mol. mass 29.5 kDa)	produce assembled apoenzyme^45,46^
UreB (mol. mass 66 kDa)	produce assembled apoenzyme^45,46^
Accessory Genes	
UreE (mol. mass 19.5 kDa)	incorporates nickel ion to urease apoenzyme^47,48^
UreF (mol. mass 28.6 kDa)	regulates folding and function of UreG^46,47^
UreG (mol. mass 22 kDa)	couples GTP hydrolysis^46,47,49^
UreH (mol. mass 29.7 kDa)	along with other accessory proteins helps in urease maturation process^50,51^
Acid Gated Channel	
UreI (mol. mass 21.7 kDa)	transport of urea^52^

#### 2.1.1 Catalytic Subunits: ureA/B

The ureA subunit is encoded by ureA gene whereas the ureB subunit is encoded by ureB genes.^45,46^ The ureA of *H. pylori* urease is unusual, as a single ureA gene encodes its amino acid sequence, whereas two separate genes encode the amino acid sequence in other bacterial species. The peculiarity could be because two smaller genes from other species may have been fused and formed ureA of the *H. pylori* urease. The ureA/B genes can encode a fully assembled apoenzyme adequately, but the apoenzyme will be catalytically inactive.^46^

#### 2.1.2 Accessory Proteins: ureE, ureF, ureG, ureH

The ureE of *H. pylori* functions as a metallochaperone and plays a crucial role in incorporating nickel ions into the urease apoprotein.^47,48^ In solution, ureE exists as a dimer and binds with 0.5 nickel ion per monomer indicating stoichiometry of 1:1 dimer to one nickel ion.^43^ UreG, another accessory protein of *H. pylori* urease functions as a GTPase that couples GTP hydrolysis and helps in urease maturation and activation process.^46,47,49^ The GTP hydrolysis is needed for transfer of nickel.^53^ The ureG contains the metal binding motif Cys–Pro–His, and substitution of this abolishes the maturation of urease. UreE with UreH supplies the nickel and helps in maturation of urease.^47^

UreF of urease in *H. pylori* regulates the folding and function of UreG which acts as a GTPase-activating protein.^46,47^ Another accessory protein, UreH, helps in urease maturation.^50,51^ The accessory proteins, ureE, ureF, ureG, and ureH are located downstream of ureA and ureB and are necessary for the urease enzyme to be active.^54^

#### 2.1.3 UreI: Acid-Gated Urea Channel

UreI of *H. pylori* is an internal proton-gated protein and plays a crucial role in urea transport and acid resistance. It has six membrane-spanning segments and histidine residues.^51,55^ UreI plays various roles in urea transport; it acts as an uptake system that helps maintain the intracellular concentration of ammonia and exports the intracellular ammonia that is in access.^52,56^ The ureI pore is regulated by external pH through a periplasmic pH shift.^57^ When the medium pH drops below pH 6.5, the ureI pore opens, allowing urea entry into the cytoplasm of the bacteria. This urease activity results in ammonia that neutralizes the acidic environment, and as the pH reaches neutrality, the urease enzyme denies the substrate urea as the ureI pore closes, avoiding the excess of ammonia.^43,58^

#### 2.1.4 Nickel

Nickel is an important nutrient required for bacteria and to ensure sufficient nickel ions for urease enzyme.^59,60^ *H. pylori* requires it as a cofactor of urease and hydrogenase.^61,62^ The homeostasis of nickel in *H. pylori* is controlled by NikR, a ribbon-helix–helix regulatory protein that acts as an activator or repressor based on the target protein.^60,63,64^ NikR has two dimeric N-terminal DNA-binding domains and a C-terminal domain required for nickel binding and tetramerization.^59,64–66^ NikR regulates gene transcription in response to nickel availability. It activates genes involved in nickel metabolism (nixA, nikABCDE, hpn-like, hpn, ureA, and ureB) and also downregulates the genes involved in iron storage and uptake (fur, pfr, and exbB/exbD), motility (flaA, flab, and cheV), and stress responses involving OMP (omp31, omp32, and omp11). The nickel ions are imported to *H. pylori* through NixA, a high affinity metal permease located in the cytoplasmic membrane.^67–69^ NixA is necessary for *H. pylori* to colonize the stomach mucosa.^67^ NixA has 331 amino acid residues accounting for a molecular weight of 37 kDa and consisting of eight transmembrane spinning helices.^67,68^

### 2.2 Roles of Urease in Pathogenesis

#### 2.2.1 Role of Urease in Colonization and Survival of *H. pylori*

Once *H. pylori* enter the human stomach, it uses a sophisticated mechanism to adapt to the acidic conditions, primarily through the urease enzyme.^70^ As the enzyme is activated, the ureI channel opens to allow entry of only urea in an acidic environment. Porins in the outer membrane of *H. pylori* is permeable to urea, and urea travels to cytoplasm through the ureI channel. This results in hydrolysis of urea to ammonia (NH_3_) and carbon dioxide (CO_2_), which diffuses into the periplasm. NH_3_ neutralizes the surrounding acidic environment and forms NH_4_ by reaction with protons. The carbonic anhydrase converts the released CO_2_ to HCO_3_^–^ which acts as a buffer.^71–73^ This dual activity helps the bacteria maintain cellular homeostasis, ensuring that biochemical processes continue without interruption.^74,75^ A study conducted by Eaton et al., in piglets revealed that the urease-negative mutant of *H. pylori* did not colonize in the gut, while the piglets that were inoculated with the parent strain of *H. pylori* had colonization ranging from 4.4 + 1.5 log_10_ CFU/g to 6.9 + 0.5 log_10_ CFU/g of gastric mucosa in piglets sacrificed after 5 days.^76^ The role of urease in the colonization and survival of *H. pylori* is depicted in Figure 1.

**Figure 1:**
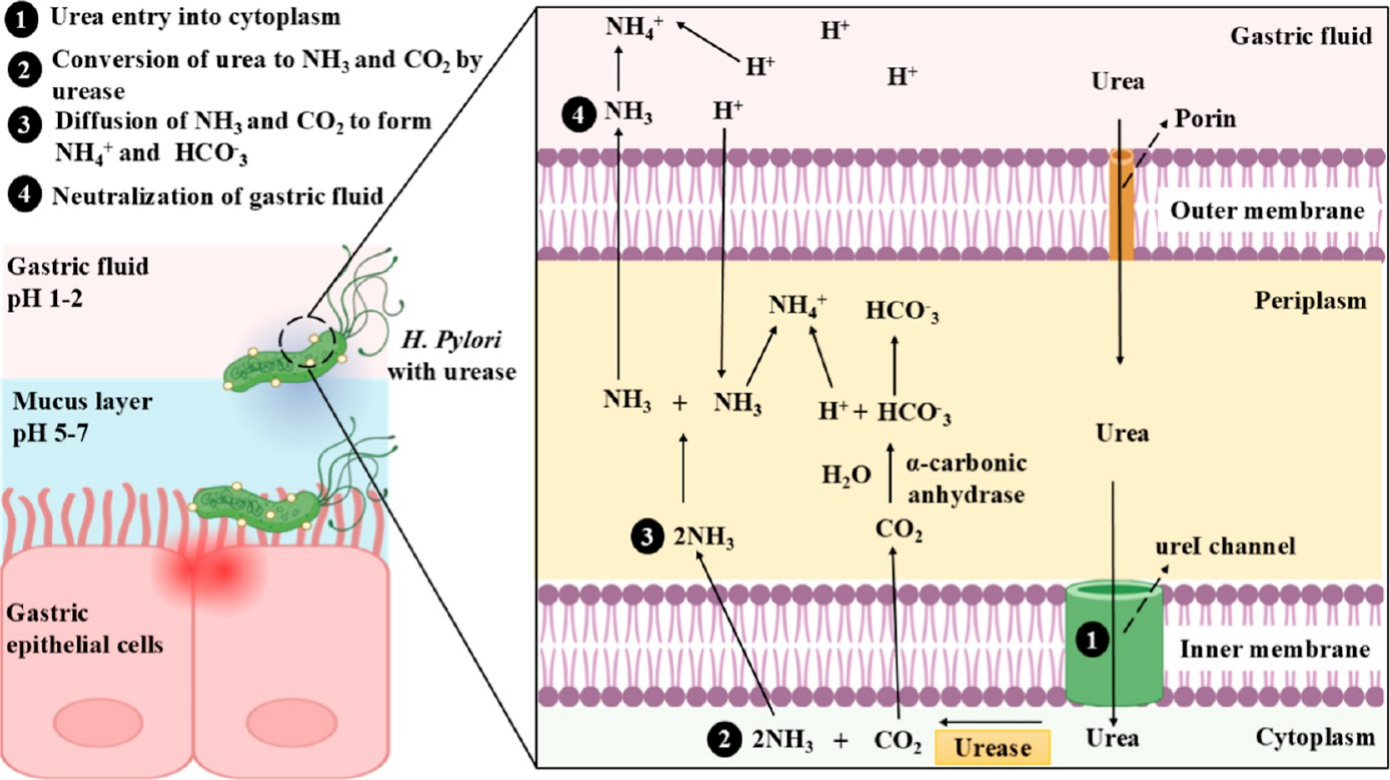
Role of *H. pylori* urease in colonization and survival of *H. pylori*.

#### 2.2.2 Role of Urease in Angiogenesis and Tumor Growth

*H. pylori* urease not only helps in survival but is also involved in the process of angiogenesis and tumor growth through the following processes. (A) Internalization and cellular activation: *H. pylori* urease is rapidly internalized into gastric epithelial cells through cholesterol-rich lipid rafts, and activates signaling events that alter cellular gene expression.^77^ (B) Direct induction of VEGF-mediated angiogenesis: *H. pylori* urease directly upregulates VEGF, a key mediator in angiogenesis, inducing new blood vessel formation and enhancing nutrient and oxygen supply to enable tumor growth.^77^ (C) Ammonia and chronic inflammation: ammonia released due to urea hydrolysis by urease enzyme leads to mucosal damage.^78^ It also increases gastrin levels and stimulate cell proliferation.^79^ *H. pylori* urease also activates neutrophils and monocytes leading to pro-inflammatory cytokines. This results in recruiting more immune cells and sustaining chronic inflammation.^77,80^ (D) Antiapoptotic effect: *H. pylori* urease exerts antiapoptotic effects in gastric epithelial cells by increasing the expression of antiapoptotic proteins like Bcl-xL (B-cell lymphoma extra large) and decreasing the expression of pro-apoptotic proteins like BAD (Bcl-2-associated death promoter), both of which are mitochondrial proteins involved in the regulation of apoptosis. This antiapoptotic activity allows genetically damaged or mutated cells to survive, further supporting angiogenesis and facilitating tumor growth.^77^ (E) Stabilization of hypoxia-inducible factor 1-α (HIF-1α): *H. pylori* urease stabilizes HIF-1α under normoxic conditions, enabling angiogenesis and tumor growth.^81^ By enhancing angiogenesis, *H. pylori* urease increases nutrient and oxygen supply to the tumor, facilitating its growth, invasion, and metastasis.^77,82^ Given its role in promoting angiogenesis, urease could be a potential therapeutic target. Inhibiting urease activity may reduce angiogenesis, thereby limiting tumor growth and spread. Role of urease in angiogenesis and tumor growth is depicted in Figure 2.

**Figure 2:**
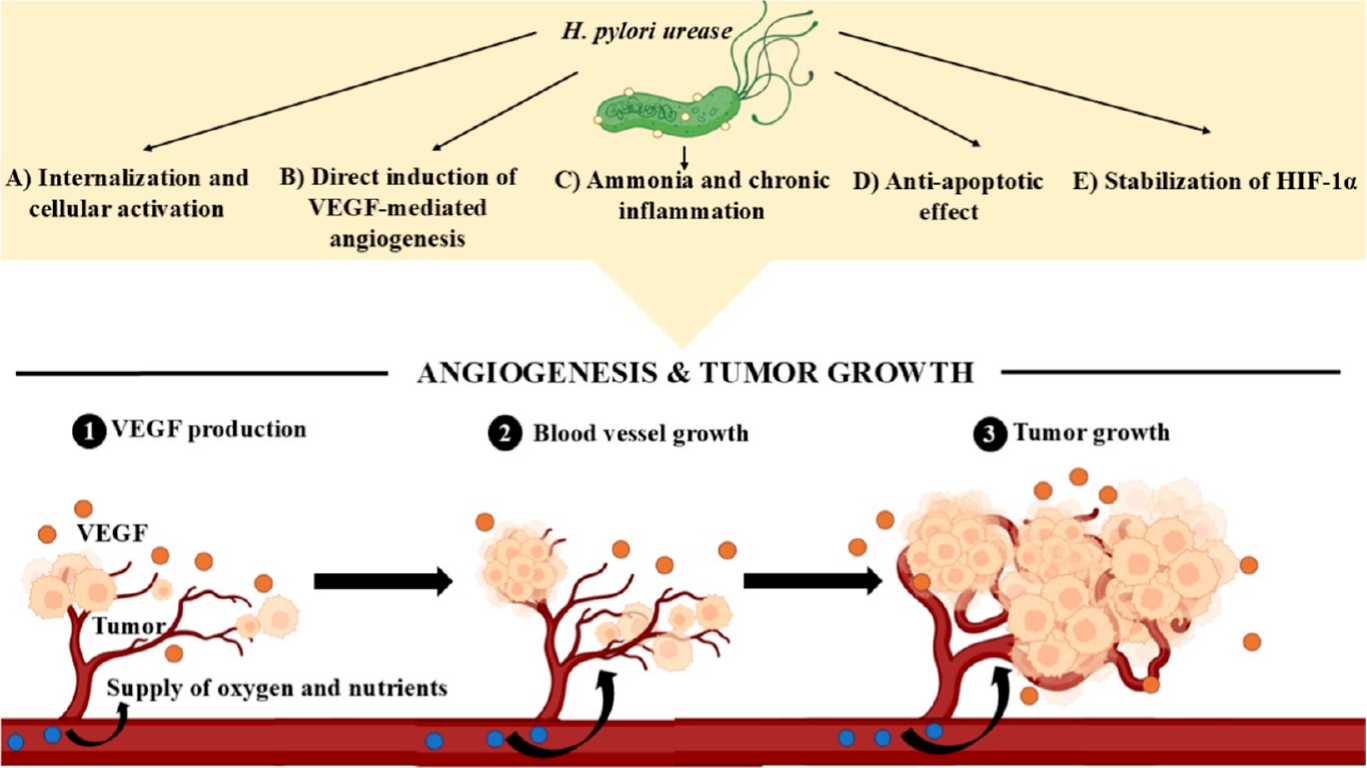
Role of *H. pylori* urease in angiogenesis and tumor growth.

#### 2.2.3 Role of Urease in Platelets Aggregation

Platelets, also known as thrombocytes, play a role in hemostasis, thrombosis, inflammatory responses, vascular integrity maintenance, and wound healing.^83^ In a healthy individual, platelet aggregation maintains hemostasis.^84^ When a blood vessel is injured, endothelial damage exposes subendothelial matrix components such as collagen and von Willebrand factor, enabling platelets to adhere to the exposed surface via receptors such as GP lb-IX-V and GP VI. This adhesion activates platelets, triggering the release of ADP, thromboxane A2 (TxA2), and serotonin from dense granules, amplifying platelet activation and recruiting additional platelets. Activated platelets undergo a morphological change and form a stable plug to stop bleeding. This process is localized and self-limiting to prevent excessive clot formation. However, in case of infection due to *H. pylori*, the urease enzyme dysregulates the platelet aggregation, leading to excessive clotting or inflammation. *H. pylori* urease binds to specific receptors on platelets, leading to the activation of calcium channels and subsequent release of ADP. Concurrently, platelets produce 12-lipoxygenase (12-LOX) metabolites, which further promotes platelet aggregation. Aggregated platelets release inflammatory mediators such as reactive oxygen species (ROS), interleukin-1 β (IL-1β), and tumor necrosis factor-α (TNF-α), which cause tissue damage. This aggregation leads to microthrombi, forming in the gastric mucosa, obstructing small blood vessels and reducing blood flow, thereby exacerbating ischemia and delaying the repair of gastric mucosal cells. Consequently, the prolonged healing process intensifies the severity and duration of gastric ulcers. Chronic inflammation resulting from this interaction can lead to conditions such as chronic gastritis and the risk of severe complications like gastric cancer due to ongoing cycles of tissue damage and regeneration.^85–88^ These effects highlight the importance of managing *H. pylori* infections by targeting its urease to prevent platelet dysfunction and associated complications. The role of urease in platelet aggregation is depicted in Figure 3.

**Figure 3:**
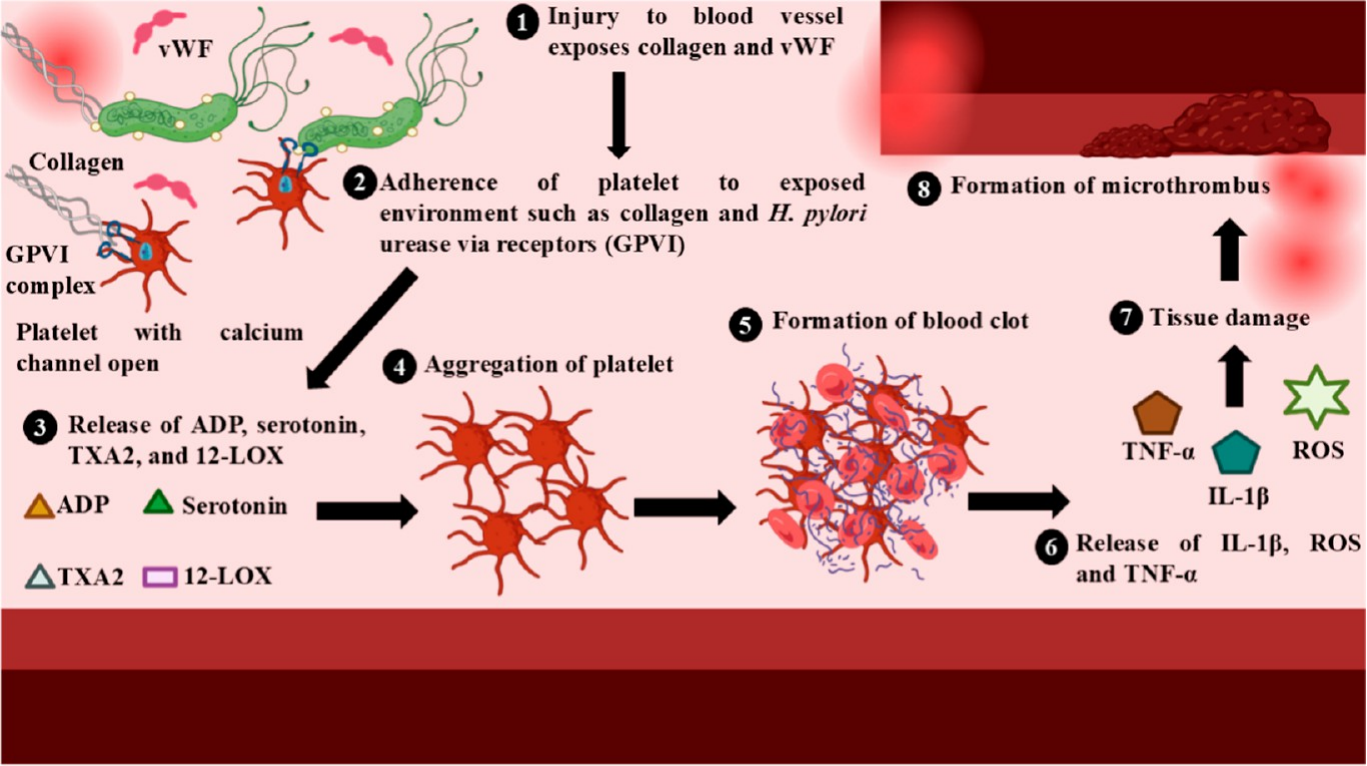
Role of *H. pylori* urease in platelet aggregation.

#### 2.2.4 Role of *H. pylori* Urease in Alzheimer’s Disease (AD)

AD is a neurodegenerative condition that causes dementia, leading to impairments in cognitive function. This results in symptoms such as aphasia (language impairment), amnesia (memory loss), agnosia (inability to recognize people, objects, or sounds despite intact sensory function), and apraxia (difficulty performing tasks or movements). AD can also cause behavioral disturbances, including personality changes, misidentifications, hallucinations, delusions, and depression.^89,90^ Emerging evidence highlights the role of the *H. pylori* urease enzyme in AD pathogenesis as it contributes to neurodegeneration. Infection with *H. pylori* elevates levels of pro-inflammatory cytokines, including TNF-α and IL-1β, which can cross the blood–brain barrier (BBB). These cytokines initiate and sustain chronic neuroinflammation, a hallmark feature of AD pathology. Chronic neuroinflammation accelerates neuronal injury and functional decline, heightening the risk of neurodegeneration.^91^ The urease activity also generates excessive ammonia by catalyzing the hydrolysis of urea into ammonia and carbon dioxide.^76^ Ammonia, a highly neurotoxic molecule, can traverse BBB and contribute to neuronal damage.^92–94^ Clinical studies have further highlighted the role of urea metabolism in neurological disorders, such as the association between elevated blood urea nitrogen and poor outcomes in ischemic stroke, underscoring the systemic impact of urea-derived toxicity.^95–97^ Tau, a microtubule-stabilizing protein in neurons, is known to become hyperphosphorylated in AD. Hyperphosphorylation of tau involves the excessive accumulation of phosphate groups, leading to neurofibrillary tangles. These tangles disrupt cellular function and are toxic to neurons, ultimately contributing to neuronal death.^98^ Study suggested that *H. pylori* urease can induce tau hyperphosphorylation in neural cells, linking enzyme to this hallmark of AD pathology.^91^ *H. pylori* urease also produces ROS thereby affecting cellular components such as proteins, lipids, and DNA. The generation of ROS directly damages neurons, exacerbates tau hyperphosphorylation, and accelerates the aggregation of amyloid β (Aβ), a key protein involved in AD. Aβ plaques, which are abnormal clumps of protein, accumulate in the brain of AD patients, contributing to neurotoxicity and dysfunction.^91^ Additionally, *H. pylori* releases outer membrane vesicles (OMVs) containing urease and other bacterial components such as lipopolysaccharides, cytotoxin-associated gene A (CagA), and vacuolating cytotoxin A (VacA).^99^ These OMVs can cross the BBB and enter the brain, promoting Aβ aggregation and plaque formation. The accumulation of these plaques is toxic to neurons and further advances AD progression.^100^ These evidence underscore the potential of targeting *H. pylori* urease as an effective therapeutic strategy for mitigating the advancement of AD (Figure 4).

**Figure 4:**
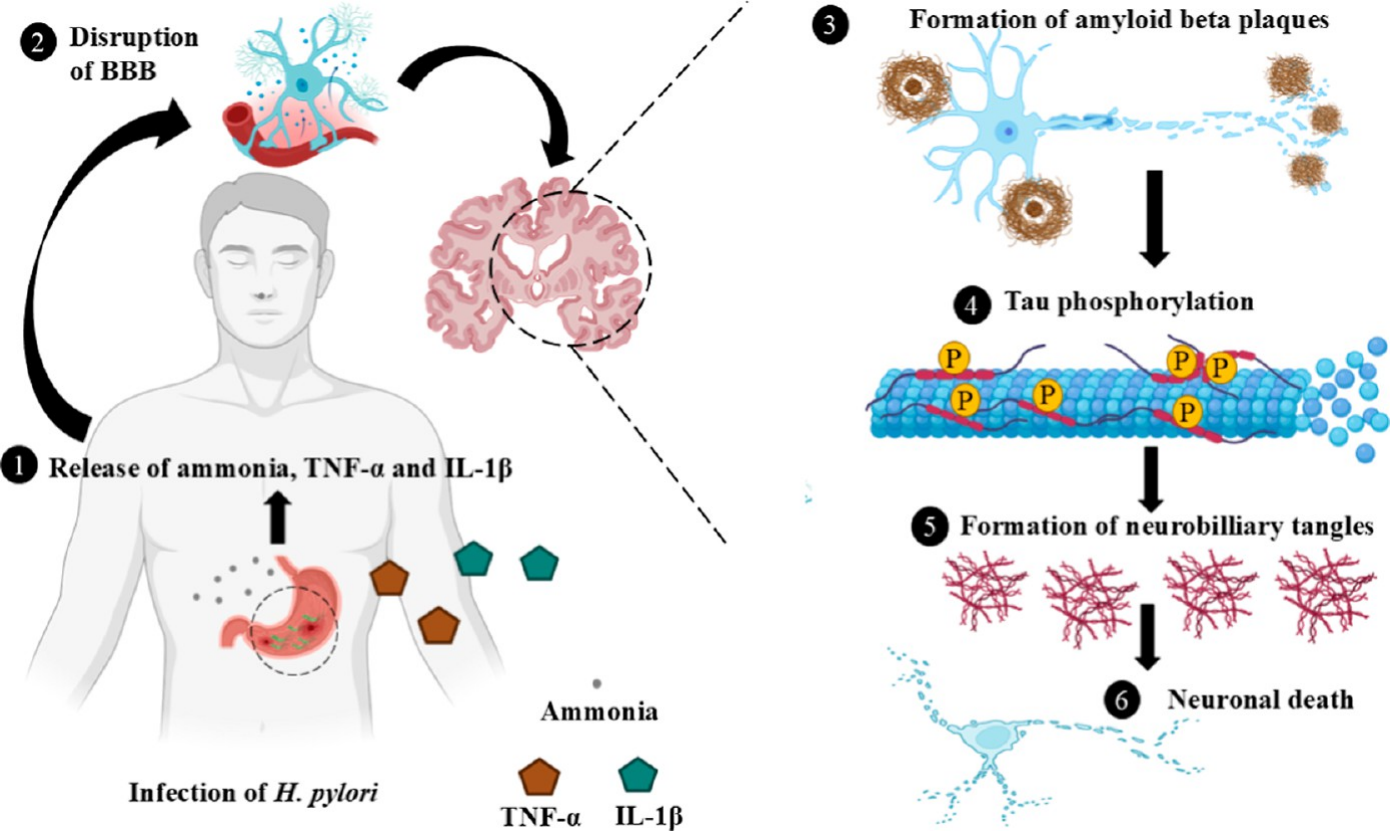
Role of *H. pylori* urease in AD.

## 3 Challenges in *H. pylori* Eradication and Potential of Urease Inhibitors in Overcoming Them

Eradication of *H. pylori* continues to be a worldwide challenge despite decades of therapeutic advancement. This is mainly attributed to increased antibiotic resistance, the coccoid form of bacteria, high bacterial load, biofilm formation, and patient-related challenges. The current treatment regimen causes side effects and also decreases the efficacy with time.^34,101^ As the effectiveness of the regimen diminishes, the risk of reoccurrence and reinfection of the bacteria increases.^102^ Urease, a crucial enzyme that enables *H. pylori* to thrive in the stomach’s acidic environment has been identified as a promising target for therapy.^1^ Urease inhibitors offer a new strategy by breaking this survival pathway, potentially enhancing the treatment efficacy. Here, we discuss the major challenges associated with the eradication of *H. pylori* and how urease inhibitors could help to overcome them.

### 3.1 Antibiotic Resistance

A primary reason for resistance of antibiotics to *H. pylori* is the insufficient reach of antibiotics to the inner layer of mucosa where it resides.^34,103–105^ The antibiotics show a topical effect for a shorter duration of time until they are present in the stomach.^105^ Resistance to metronidazole, clarithromycin, and levofloxacin is increasingly on the rise, particularly after failed eradication attempts.^106^ Research indicates that resistance to clarithromycin and levofloxacin is associated with a 7-fold and 8.2-fold increase in treatment failure respectively, in both treatment-naive and refractory infections.^107^ The primary resistance rates of clarithromycin, amoxicillin, tetracycline, metronidazole, and levofloxacin are 34.1–55.2%, 15.0%, 17.9%, 69.4–71.3%, and 18.4–27.9%, respectively.^34^ This increasing pattern of resistance creates a difficult cycle in which each failed treatment further reduces the effectiveness of future therapies.

### 3.2 The Coccoid Form of *H. pylori*

Most of the *H. pylori* bacteria are spiral in shape but under unfavorable conditions and on long-term contact with antibiotics, *H. pylori* convert into coccoid form. Coccoid form of *H. pylori* contributes to immune invasion, allowing the bacterium to persist undetected and sustain chronic infection.^108,109^ These coccoid forms may continue to remain dormant for a long period of time in the gastric tissues and can retain virulence factors. This may lead to failure of treatment and reinfection of *H. pylori*.^108,109^ Among amoxicillin, metronidazole, and clarithromycin, it is reported that amoxicillin showed pronounced effect with more than 90% conversion to coccoid from at 1/2 MIC after 72 h, and lead to treatment failure after suboptimal therapy.^109,110^ It is essential to explore unconventional antimicrobial approaches, especially in the case of recurrent infection.

### 3.3 High Bacterial Load

Increased *H. pylori* density is associated with more intense inflammation and higher risk of diseases.^111^ Certain virulence factors, including the cagA gene, are associated with increased bacterial loads in the gastric mucosa.^112^ Clinical studies conducted by Lai et al., demonstrate that pretreatment density of *H. pylori* considerably influences eradication as well as ulcer healing rates. In patients with mild and moderate *H. pylori* density, eradication rate of *H. pylori* was 88.9%, and 94.3% respectively. While, the eradication rate was only 69.7%, in patients with high bacterial density.^113^ These results point out the direct association of the bacterial load and treatment outcome.

### 3.4 Biofilm Formation

*H. pylori* forms a biofilm, protecting it from antibiotics.^114^ Biofilms are microorganism communities attached to the surface and embedded by matrix composed of polysaccharide, lipids, DNA, and proteins that acts as shield, limiting antibiotic penetration and protecting bacteria from host immune systems.^115–117^ Biofilm of *H. pylori* also has efflux pump genes such as HP1181, HP1165, hefA, and gluP that expels the antibiotic before reaching bacterial cells contributing to multidrug resistance.^118^ Biofilms reduce the diffusion of antibiotics, requiring 10–1000 times higher antibiotic concentration as compared to planktonic bacteria.^119^ It is reported that biofilm leads to antibiotic resistance.^120^ As a result, we should consider the impact of biofilm formation while developing a new drug against *H. pylori*.

### 3.5 Complexity of Therapy and Patient Noncompliance

Standard triple or quadruple therapies demand patients to take multiple medications several times in a day for up to 2 weeks or beyond. The complexity and pill burden overwhelm patients leading to missed doses or early discontinuation.^121^ Eradication of *H. pylori* depends strongly on patients receiving at least 90% of the prescribed medication. Noncompliance is greater with a more prolonged or complicated regimen, with rescue treatment, and when the patient experiences adverse effects. Evidence indicates that approximately 1.7% to 10% of patients fail to complete treatment as directed, with less compliance in regimens involving multiple doses or large pill burdens.^122^ Adverse effects related to the prolonged use of antibiotics also lead to discontinuation of treatment. Common noncompliance includes the severe diarrhea abdominal pain associated with amoxicillin and levofloxacin, appearance of metallic taste associated with metronidazole and clarithromycin, mineralization and bone calcification of tetracycline and the bitter taste, nausea, and dark stools associated with bismuth.^123–126^

### 3.6 Targeting Urease as a Promising Approach to Overcome the Challenges

Targeting the urease enzyme offers a promising strategy to overcome the above challenges. Since urease is pivotal for gastric survival, inclusion of a urease inhibitor as an adjuvant with antibiotics can provide an additive antimicrobial effect, resulting in eradication of the organism and prevent the development of resistance. This adjuvant approach does not interfere with the mechanism of antibiotic action and thus provides an additive effect.^127^

The coccoid form of *H. pylori* is a dormant, nonreplicative state with lowered metabolism than the spiral form.^128,129^ By directly inhibiting urease, these dormant forms can be deprived of their primary acid-neutralizing mechanism, impairing their capacity to survive in the stomach leading to their eradication where antibiotics are not effective.^127^

High bacterial loads overwhelm the host’s defenses and diminish antibiotic effectiveness, particularly when there is resistance. Some urease inhibitors like acetohydroxamic acid and baicalin have been reported to profoundly decrease the viability of *H. pylori* and ATP synthesis irrespective of bacterial load.^127^ By inhibition of a key survival process, urease inhibition can reduce the bacterial burden and augment the efficacy of antibiotics.

Biofilms shield *H. pylori* from antibiotics and immune responses of the host, allowing for chronic infection and recurrence. The activity of the urease is responsible for biofilm development by establishing a localized pH-neutral environment.^130^ Inhibition of urease dismantles this protective niche, making bacteria within biofilms more susceptible to immune clearance and antimicrobials.

Today’s standard regimen tend to involve several antibiotics and proton pump inhibitors which results in a high pill burden and complicated dosing schedule, both of which can have negative effects on adherence.^131,132^ Urease inhibitors, specifically attacking one of the most vital survival mechanism of bacteria, is expected to decrease the number of antibiotics needed, making the regimen simpler and can help in reducing the adverse effects.^127,133^ Less complicated regimen are highly correlated with improved adherence.^131,134^ Role of urease inhibitors in overcoming the challenges of *H. pylori* eradication is depicted in Table 2.

**Table 2:** Potential Role of Urease Inhibitors to Overcome the Challenges Associated with *H. pylori* Eradication

sr. no.	challenges	overcoming the challenges by urease inhibitors
1	antibiotic resistance	bypasses antibiotic resistance mechanism
2	bacterial coccoid form	reduces acid resistance, targets dormant cells
3	high bacterial burden	reduces viability and ATP production
4	biofilm formation	disrupts biofilm stability and persistence
5	poor adherence to therapy	simplify the regimen and reduce the pill burden
6	complexity and duration of treatment	fewer drugs and shorter courses
7	adverse effects	lower doses, improved tolerability

## 4 Three-Dimensional Structure of *H. pylori* Urease

Understanding the structure of *H. pylori* urease is vital in designing targeted urease inhibitors, as it reveals the enzyme’s active site architecture, enabling the design of compounds that can block its function. The two subunits, α and β, make up the urease. These two subunits combine to form heterodimer αβ. The heterodimeric unit can then form a trimeric structure (αβ)_3_, which later forms tetrahedral structure ((αβ)_3_)_4_ or a dodecameric assembly having 12 active sites. These active sites have two nickel ions, and this region is also denoted as “flap” region since it has the flexibility to change from the closed state to the open state to provide access to urea at this two-nickel region.^135^ Figure 5 depicts the formation of the structure of the urease and the flap region in the urease’s active site.

**Figure 5:**
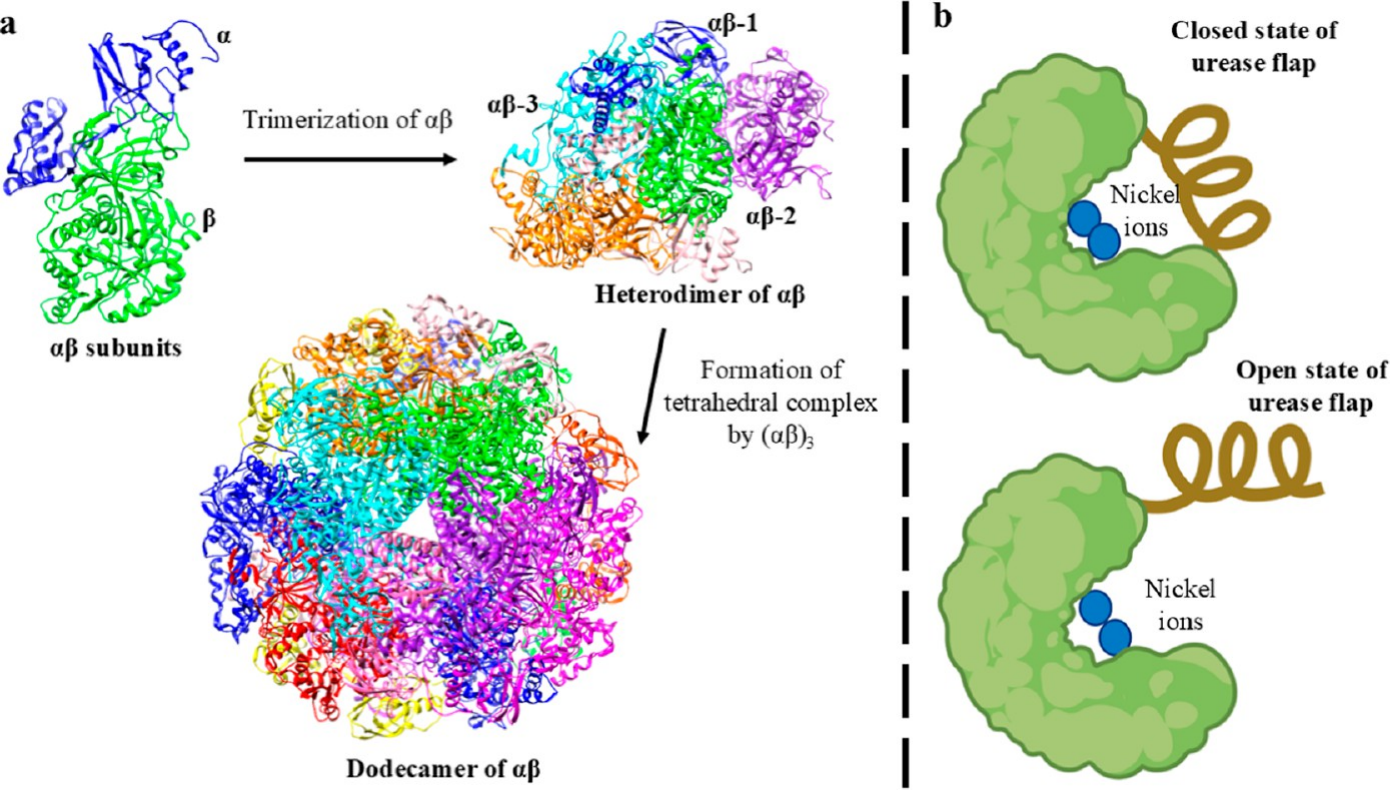
Three-dimensional structure of urease enzyme (a) formation of dodecameric assembly of *H. pylori* urease (b) flap region of active site of urease in closed and open state.

The study on *H. pylori* urease has advanced through homology modeling, structural experimental studies, and computational drug design to identify novel inhibitors. Homology modeling was the first tool to understand the urease of *H. pylori*. The crystal structure of *H. pylori* urease (Protein Databank Bank (PDB) 1E9Z) was modeled from *Klebsiella aerogenes* (PDB 1KAU) as it is homologous to *K. aerogenes*. This structure helped the researchers understand the interaction of the known urease inhibitor, acetohydroxamic acid, in the *H. pylori* urease active site. The residues present in *H. pylori* urease active sites have four histidines (His138, His136, His274, and His248), one aspartic acid (Asp362), one carbamylated lysine (KCX219), and two nickel ions. The *H. pylori* urease in complex with ligand acetohydroxamic acid (HAE) was determined by X-ray diffraction technique at 3.00 Å and it is deposited in the PDB with the ID 1E9Y.^45,135^ Similarly, the cryo-EM structures of *H. pylori* urease with ligands 2-{[1-(3,5-dimethylphenyl)-1*H*-imidazol-2-yl]­sulfanyl}-*N*-hydroxyacetamide (DJM) and β-mercaptoethanol (BME) determined at 2.00 Å and 2.40 Å resolution are also deposited in PDB under the accession ID 6ZJA and 6QSU, respectively. The active site residues of *H. pylori* urease bound to ligands HAE, DJM, and BME are shown in Figure 6. The PDB IDs of *H. pylori* urease bound to ligands along with details on mutation, their activity, and active site residues are given in Table 3.

**Figure 6:**
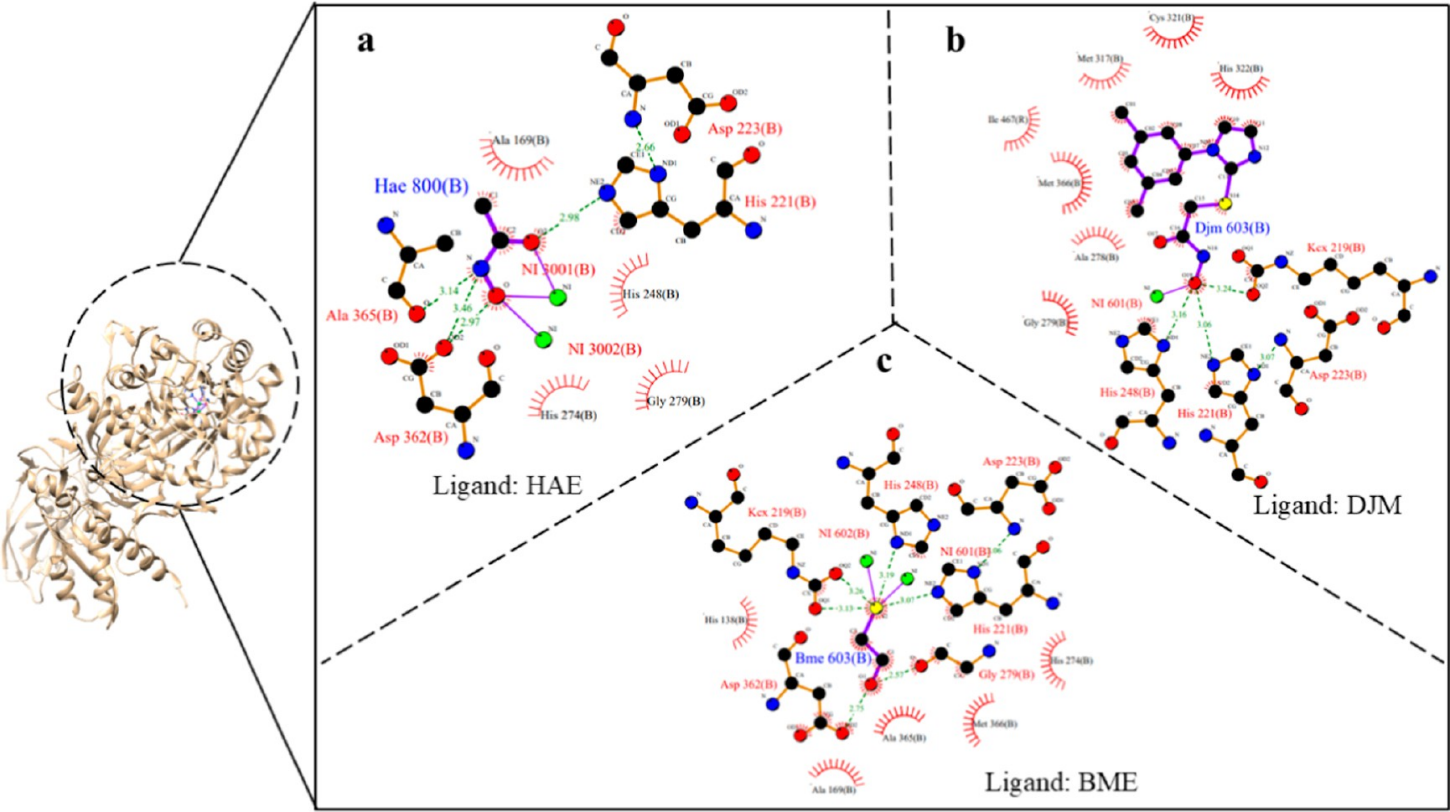
Ligplot diagram of (a) acetohydroxamic acid (HAE), (b) 2-{[1-(3,5-dimethylphenyl)-1*H*-imidazol-2-yl]­sulfanyl}-*N*-hydroxyacetamide (DJM), and (c) β-mercaptoethanol (BME) in the *H. pylori* urease active site.

**Table 3:** List of PDB ID of *H. pylori* Urease Bound to the Ligand with Details of Mutation and Active Site Residues

PDB ID	method used for determination of protein structure	resolution (Å)	pH	mutation	ligand	ligand activity	active site residues (as per PDBsum)
1E9Y^45^	X-ray diffraction	3.00	6.5	*N*6-carboxylysine is modified residue at position 219 of ureB	acetohydroxamic acid	*K*_i_: 96,000 ± 17,000 nM, IC_50_: 156 ± 32 μM	histidines (His221, His274, His248), aspartic acid (Asp362, Asp223), alanine (Ala169, Ala365) and glycine (Gly279)
6ZJA^136^	electron microscopy	2.00		no	2-{[1-(3,5-dimethylphenyl)-1*H*-imidazol-2-yl]sulfanyl}-*N*-hydroxyacetamide	*K*_i_: 0.630 μM, IC_50_: 19.6 μM	methionine (Met366, Met317), isoleucine (Ile467), aspartic acid (Asp223), histidine (His322, His248, His221), carbamylated lysine (KCX219), cysteine (Cys321), alanine (Ala278), and glycine (Gly279)
6QSU^136^	electron microscopy	2.40		no	β-mercaptoethanol	*K*_i_: 435 μM, IC_50_: 13.5 mM	histidines (His221, His138, His274, His248), aspartic acid (Asp362, Asp223), alanine (Ala169, Ala365) and glycine (Gly279)

## 5 Urease Inhibitors for Treatment of *H. pylori* Infection

Inhibiting urease enzyme activity has been explored by many researchers to prevent pathogenesis and make the organism less adaptable to the gastric acidic environment.^137–141^ In this section, we discuss the potent urease inhibitors based on their structural class. The reported half-maximal inhibitory concentration (IC_50_) values, type of inhibition, the key pharmacophoric features and its significance are highlighted in Table 4. Compounds showing urease inhibitory activity belonging to the classes imidazole, hydroxamic acid, flavonoid, barbituric acid, thiourea, coumarin, catechol, and chlorogenic acid derivatives are discussed.

**Table 4:**
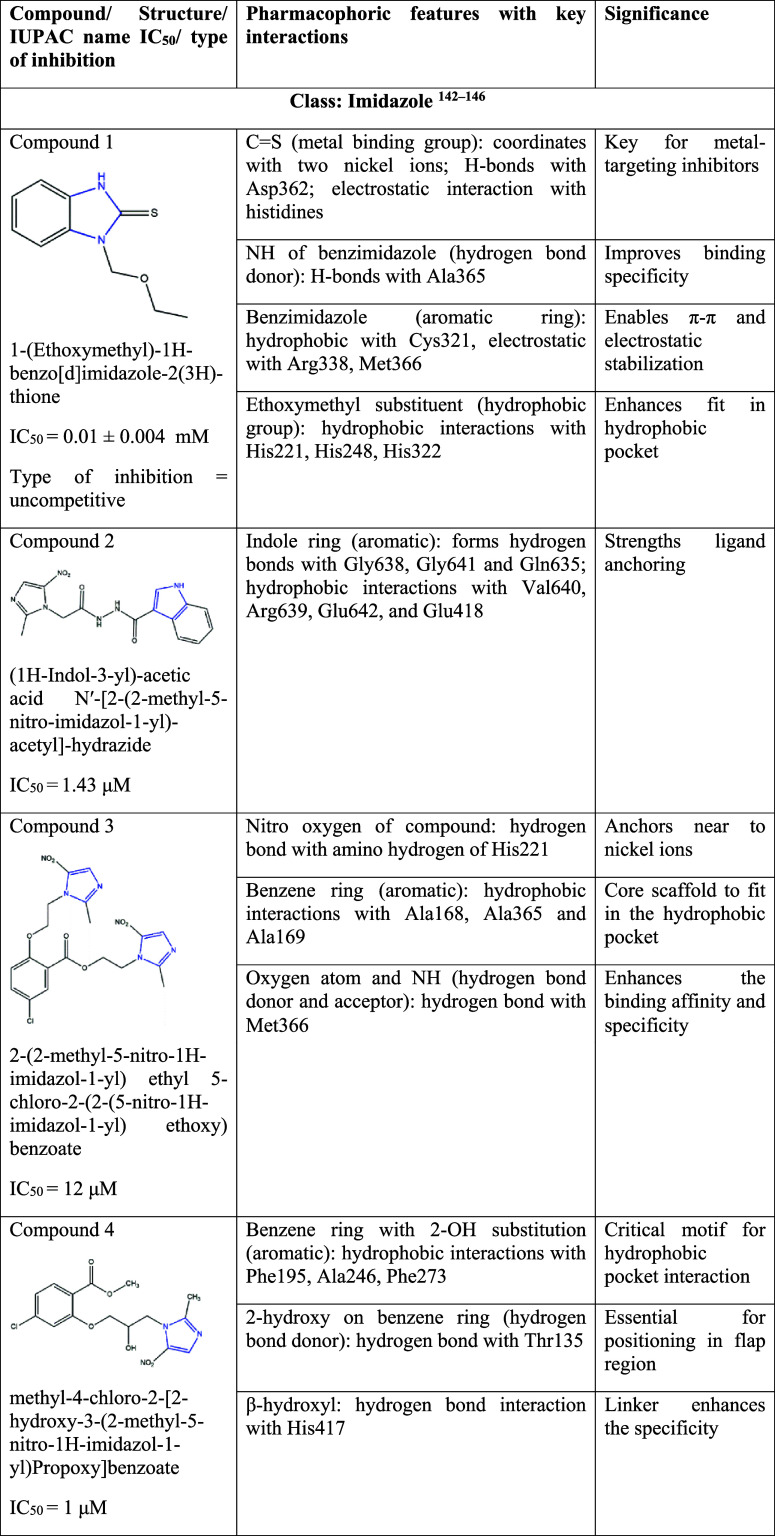

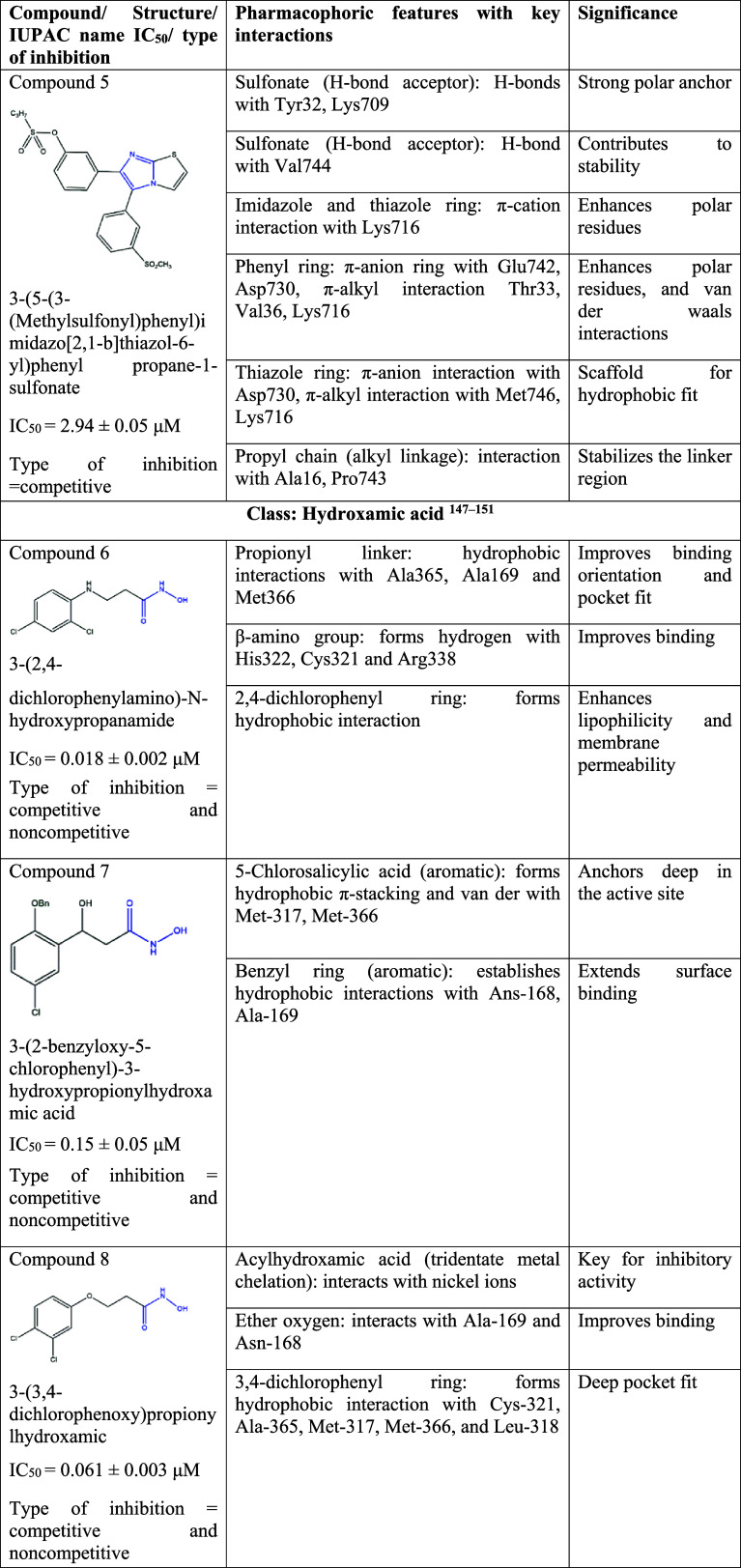

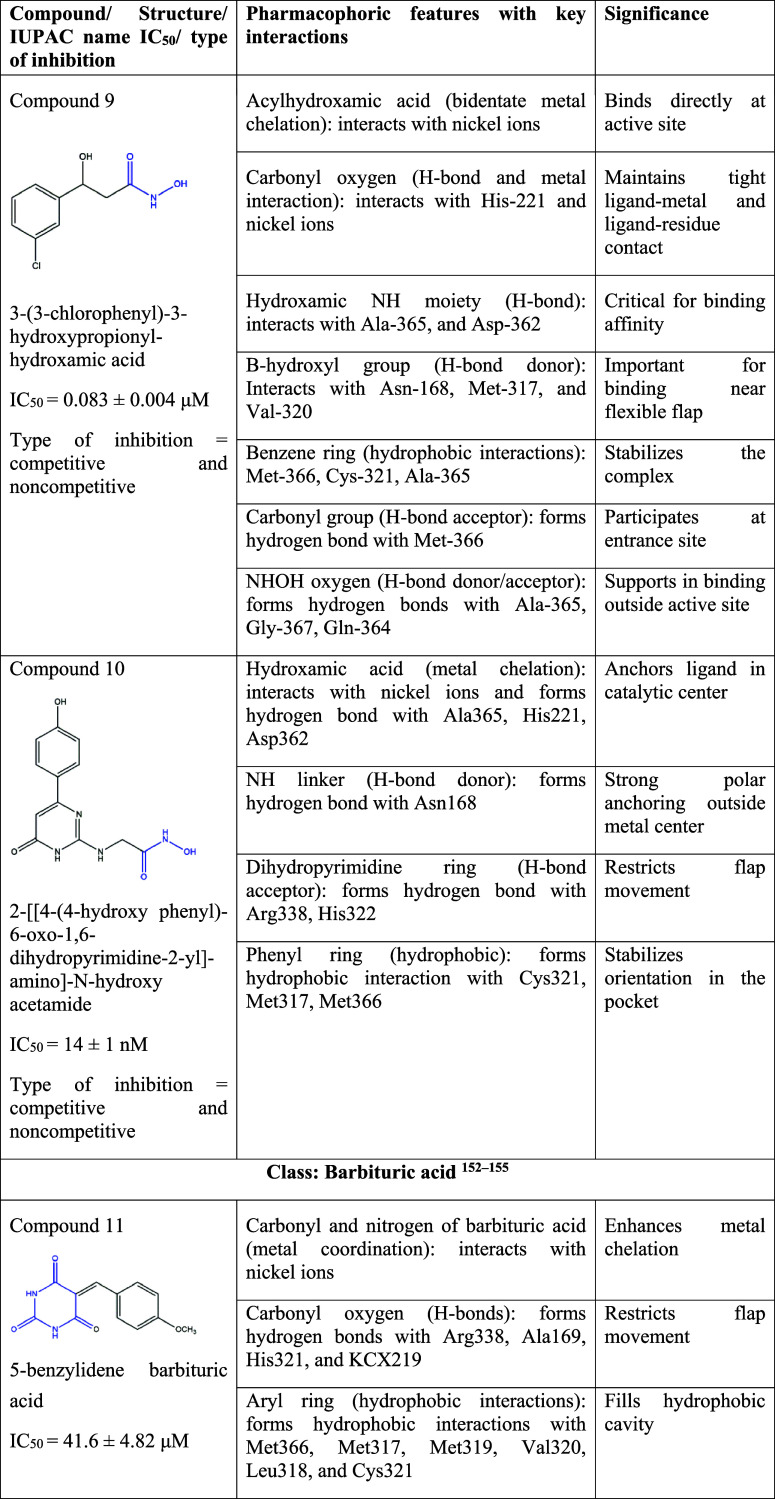

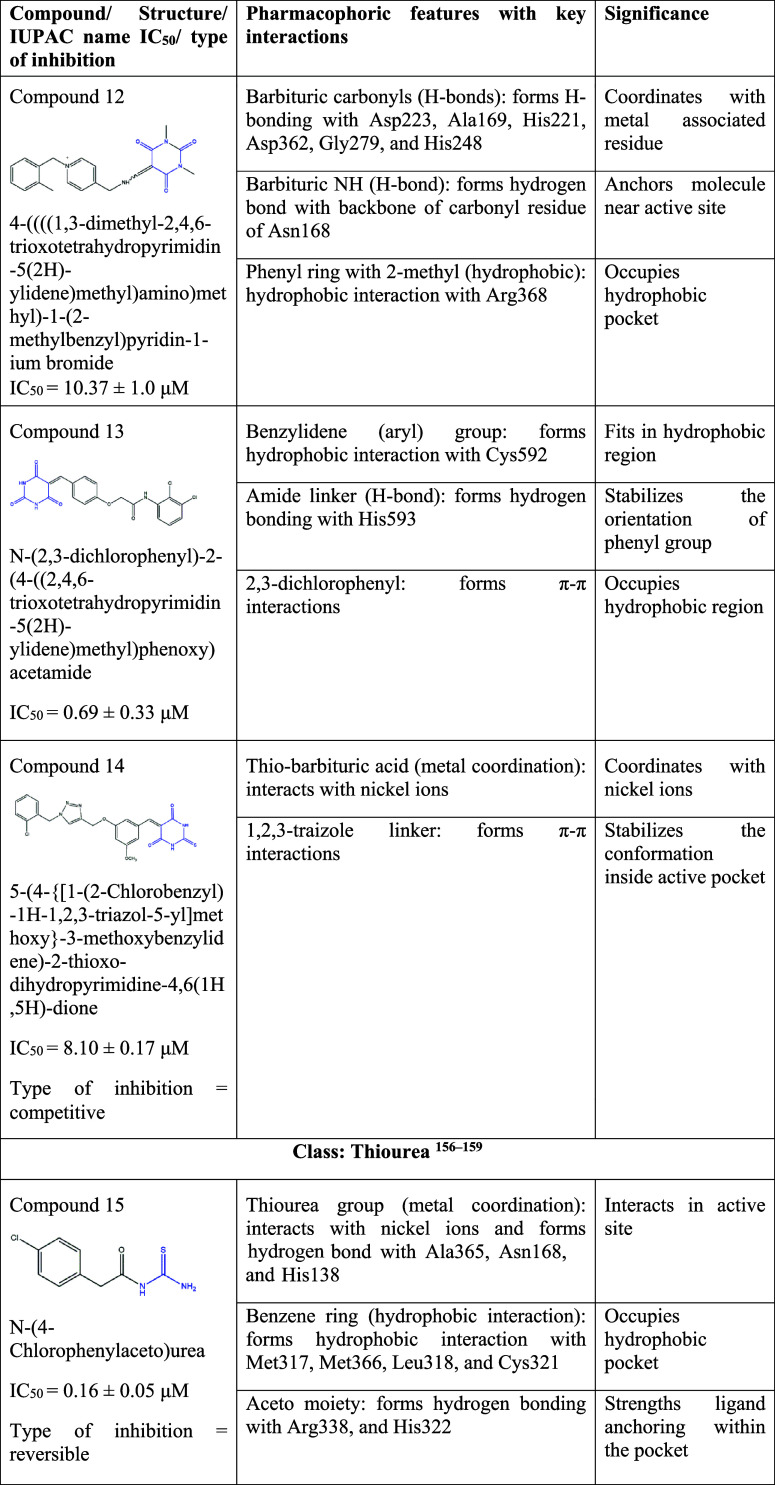

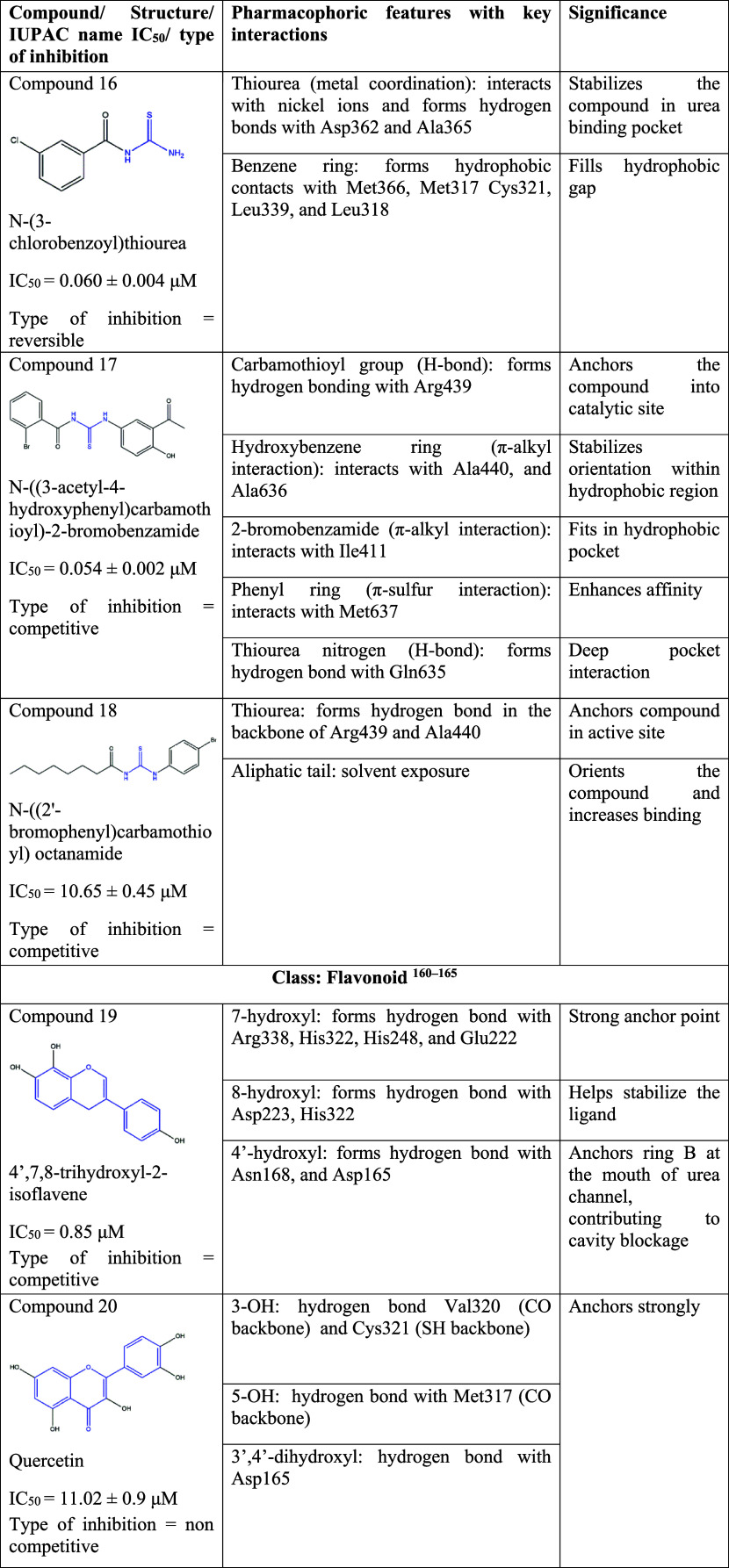

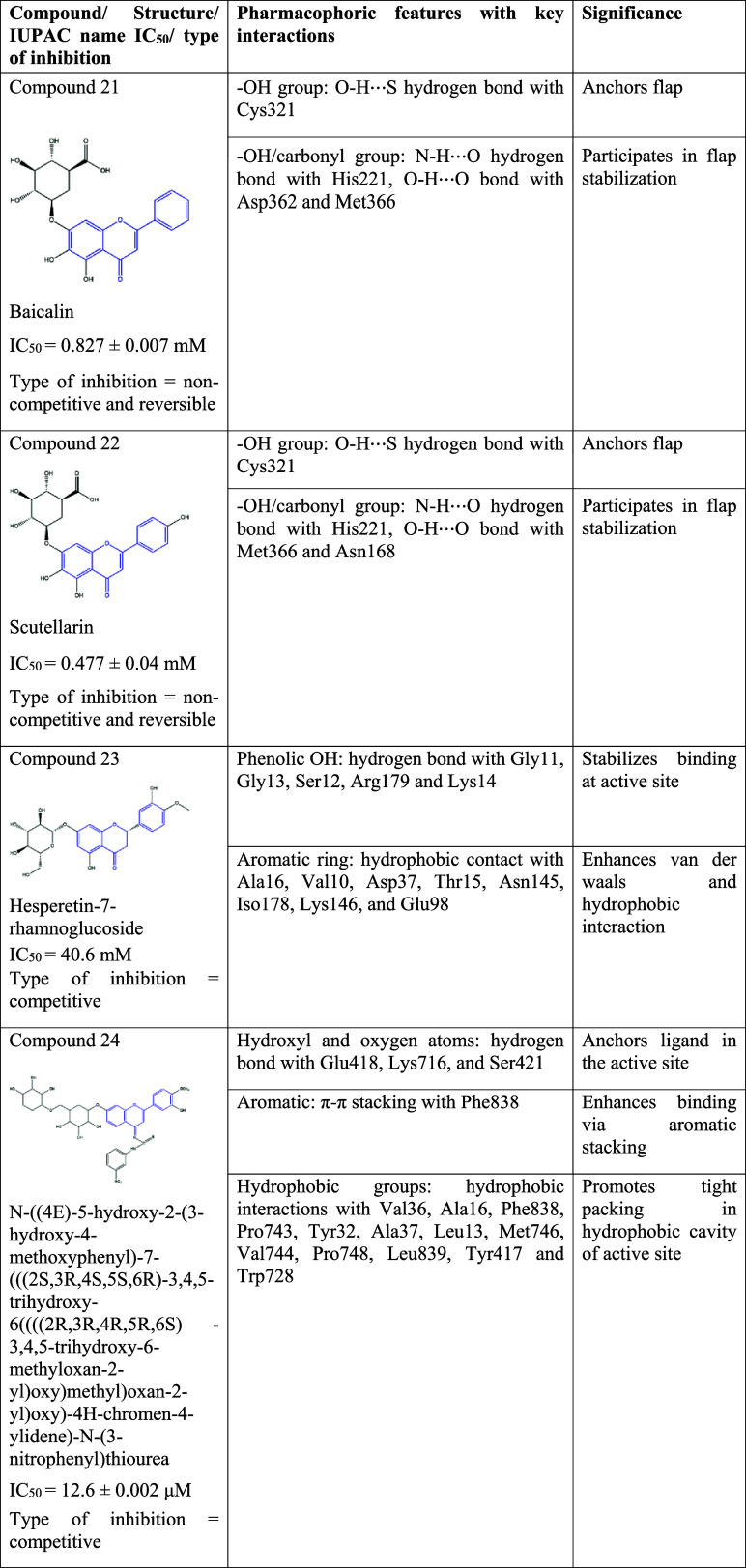

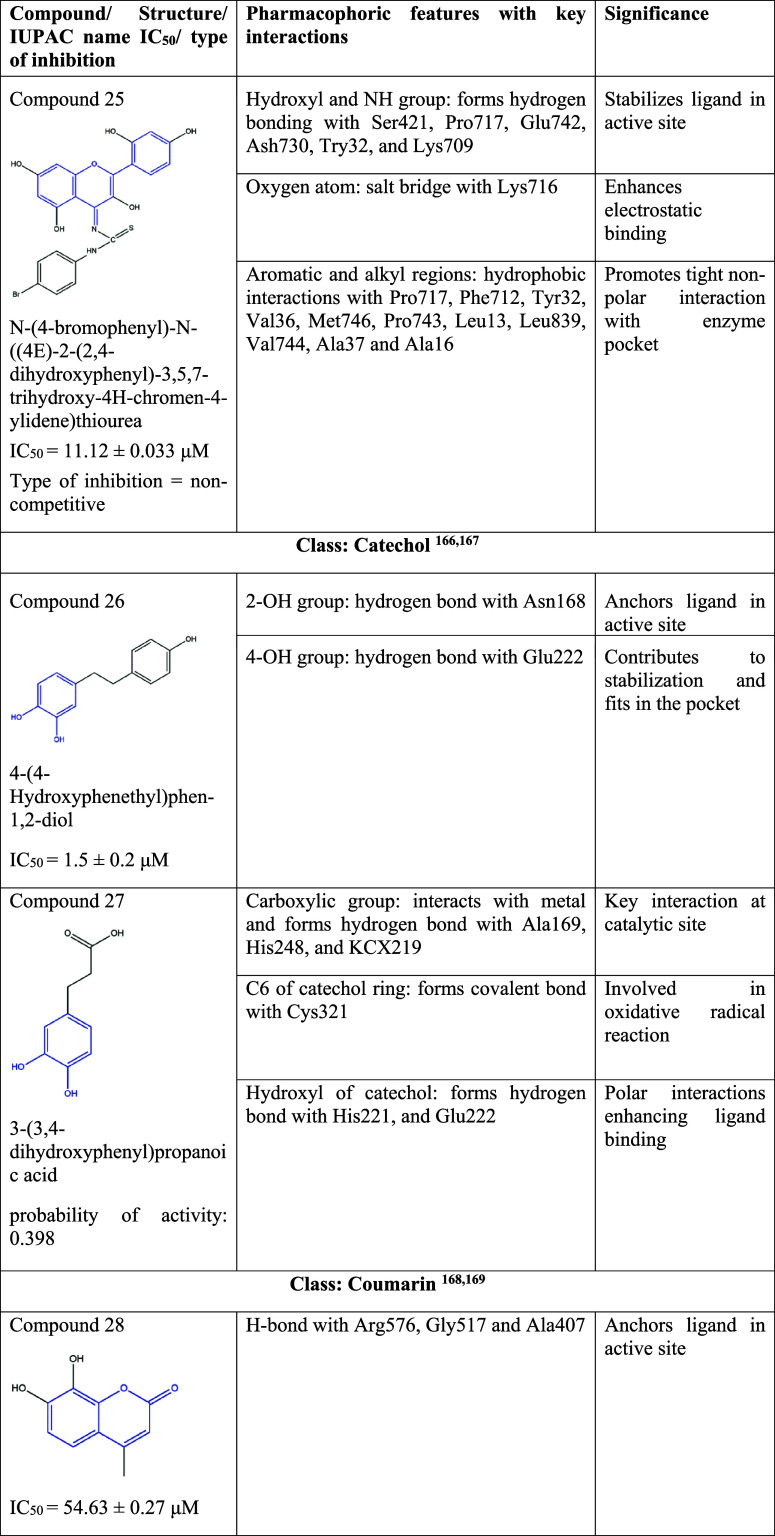

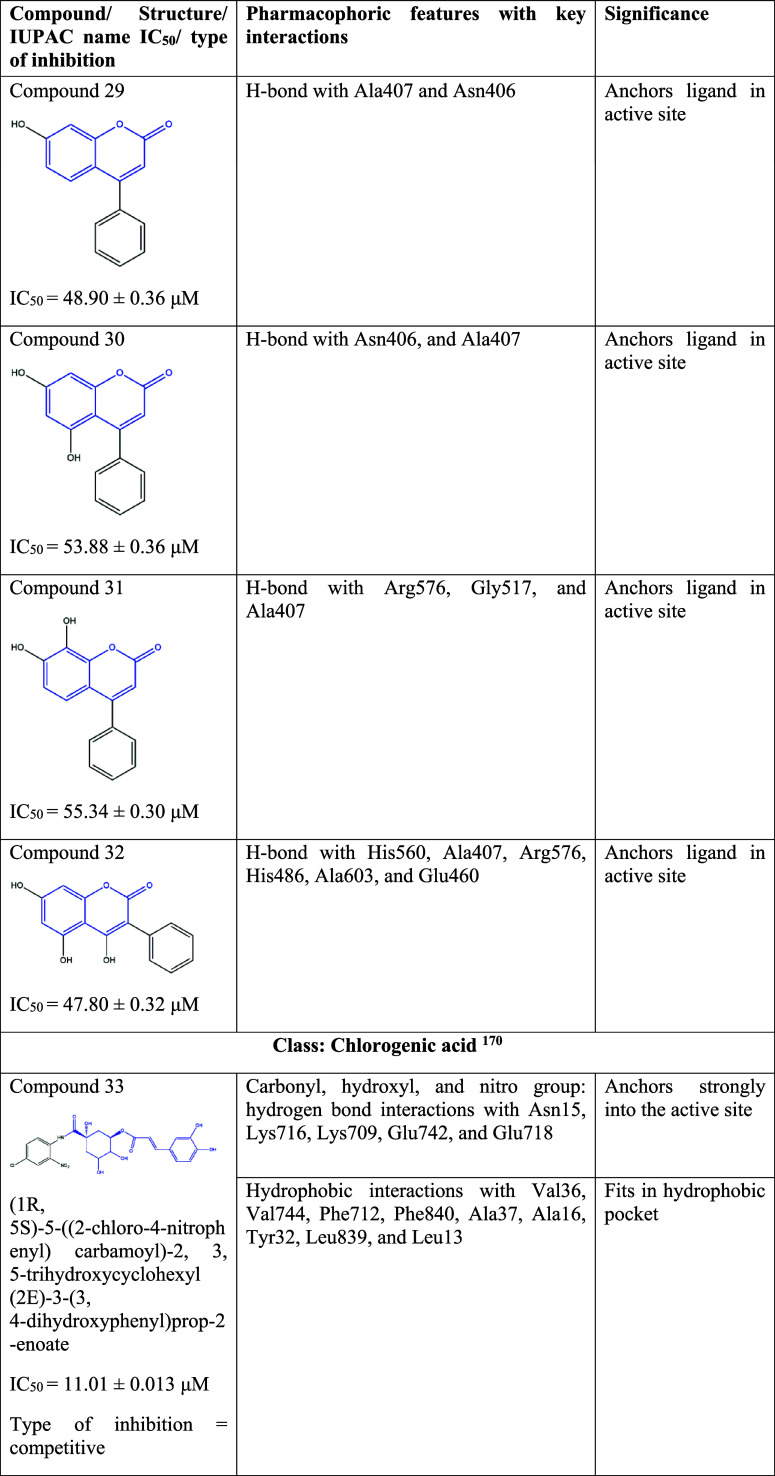
Urease Inhibitors with Their IUPAC Name, IC_50_ Value, Type of Inhibition, Pharmacophoric Features with Key Interactions and Their Significance^162^

### 5.1 Imidazole and Its Derivatives

Imidazole derivatives, characterized by their nitrogen-containing heterocyclic ring, have gained attention in research due to wide range of biological activities like antifungal, anticancer, anti-HIV, antitubercular, antibacterial, and analgesic effects.^171^ Notably, imidazole as a scaffold has been used for the design and synthesis of *H. pylori* urease inhibitors. Among these derivatives, five compounds showed promising activity (Table 4). A benzimidazole-2-thione derivative (**Compound 1**), showed a high binding free energy of −17.20 kcal/mol as compared to standard acetohydroxamic acid (binding free energy = −7.78 kcal/mol) in docking studies (PDB: 1E9Y). Docking interactions suggest a structural requirement of thiocarbonyl, benzimidazole, and ethoxymethyl groups as essential for activity in **Compound 1**. The in silico ADMET (adsorption, distribution, metabolism, excretion, and toxicity) and cytotoxicity study confirmed the safety.^142^ A nitroimidazole derivative, (**Compound 2**), demonstrated a 15-fold and 70-fold increase in urease inhibition compared to thiourea (IC_50_ of 22.01 μM) and hydroxyurea (IC_50_ of 100 μM), respectively. The docking study (PDB: 3LA4) showed binding energy of −9.54 kcal/mol revealing the presence of indole ring as a structural requirement for target affinity. Furthermore, the ADME studies also highlight its drug likeness.^143^ A metronidazole salicylate derivative (**Compound 3**), showed a 1.4-fold increase in urease inhibition as compared to standard acetohydroxamic acid (IC_50_ of 17 ± 2 μM). The docking studies (PDB: 1E9Z) supported the role of halogenated aromatic system and ether linkages essential for activity.^144^ A secnidazole salicylate conjugate (**Compound 4**), showed 16-fold increase in activity compared to acetohydroxamic acid (IC_50_ of 16 ± 2 μM). Docking studies (PDB ID: 1E9Z) established hydroxyl-substituted benzene rings key for activity.^145^ An imidazole­[2,1-*b*]­thiazole-sulfonate derivative, (**Compound 5**), was found to increase urease inhibition by 8-fold than standard thiourea (IC_50_ of 22.3 ± 0.031 μM). The docking study (PDB: 3LA4) of this compound revealed *N*-propyl and mesyl phenyl groups of the compound important for enzyme binding. Molecular dynamics also confirmed the stability of protein and its complexes with the compound.^146^

### 5.2 Hydroxamic Acid Derivatives

Hydroxamic acids are a class of compounds with a wide range of biological activities. Derivatives belonging to this class exhibit remarkable inhibitory properties targeting peroxidase, matrix metalloproteinase, and urease. They also function as siderophores, competing for iron­(III).^172^ Hydroxamic acid represents a promising avenue in managing *H. pylori* infection by targeting the urease enzyme. Acetohydroxamic acid from this class is used as a reference compound in studies to target the urease enzyme. Researchers have developed urease inhibitors based on the hydroxamic acid scaffold. In this discussion, we highlight five compounds exhibiting potent urease inhibitory activity (Table 4). An *N*-arylamino hydroxamic acid derivative, (**Compound 6**), exhibited 100% eradication rate showing 1500-fold more efficacy than standard acetohydroxamic (44.4% eradication rate). Docking studies (PDB: 1E9Y) revealed 2,4-dichloro substitution on benzene is essential for urease inhibition. The acute toxicity studies in mice showed LD_50_ of 3126.9 mg/kg supporting its potential for clinical development.^148^ A 3-arylpropionylhydroxamic acid derivative (**Compound 7**), demonstrated 20-fold increase in activity than standard acetohydroxamic acid (IC_50_ of 23.8 ± 1.5 μM). Docking studies (PDB: 1E9Y) of this compound revealed 5-chlorosalicylic acid and benzyl group as an essential structural feature for urease inhibition.^149^ A phenoxyacylhydroxamic acid derivative (**Compound 8**), showed 457-fold increase in the urease inhibitory activity than the standard acetohydroxamic acid (IC_50_ of 27.9 ± 1.5 μM). The docking studies (PDB: 1E9Y) showed the acylhydroxamic moiety, ether oxygen atom, and 3,4-dichlorophenyl group essential for potent inhibitory activity.^150^ A hydroxamic acid derivative containing a chromone ring system, (**Compound 9**), exhibited a 332-fold increase in activity as compared to acetohydroxamic acid (IC_50_ value of 27.6 ± 2.5 μM). Docking studies (PDB: 1E9Y) revealed that the compound can act in both, anionic and neutral forms, targeting the active site and the flap region, leading to potent inhibition.^151^ A dihydropyrimidine hydroxamic acid derivative, (**Compound 10**), demonstrated ∼2000-fold higher urease inhibitory activity than the commercially available acetohydroxamic acid (IC_50_ value of 27.4 ± 1.2 μM). Docking studies revealed that hydroxamic acid moiety, NH linker, carbonyl of dihydropyrimidine, and phenyl ring are essential for enhancing the activity.^147^

### 5.3 Barbituric Acid Derivatives

Barbituric acid is a versatile heterocyclic compound with a pyrimidine ring with three carboxyl groups and diverse pharmacological and biological applications. It serves as a core scaffold for synthesizing barbiturates, a class of drugs historically utilized as hypnotics, anticonvulsants, and sedatives. Barbituric acid derivatives exhibit activities such as antileprotic, antihistamine, antiurease, anti-inflammatory, antiviral, antioxidant, anti-AIDS, anesthetic, anticonvulsant, sedative-hypnotic, antimicrobial, anticancer, and antitumor properties.^173^ Barbituric acid has also been explored for *H. pylori* urease inhibitory activity. Among the barbituric acid derivatives, four compounds showed potent urease inhibition (Table 4). A 5-benzylidene barbiturate (**Compound 11**), exhibited an increase in activity of 2.4-fold compared to standard hydroxyurea (IC_50_ = 100 ± 3.03 μM). The docking study (PDB: 1E9Y) showed docking score of −9.47 kcal/mol revealing para-substitution important for enhancing the urease inhibition than ortho or meta substitution.^152^ An *N*,*N*-dimethylbarbituric-pyridinium derivative (**Compound 12**) showed an increase in urease inhibition of 2.2-fold and 10-fold in comparison to standard thiourea (IC_50_ value of 22.0 ± 0.03 μM) and hydroxyurea (IC_50_ of 100.0 ± 0.2 μM), respectively. Docking studies revealed that *N*,*N* dimethyl substitution, pyridinium ring, and 2-methylphenyl moieties are involved in potent urease inhibition.^153^ A thio-barbiturate, (**Compound 13**) exhibited 144-fold and 33-fold increase in urease inhibition as compared to standard urease inhibitors, such as hydroxyurea (IC_50_ of 100 ± 1.7 μM) and thiourea (IC_50_ of 23 ± 0.73 μM). Docking studies (PDB: 4H9M) showed that the benzylidene group, amide linker, and 2,3-dichlorophenyl have enhanced the urease inhibition. The in silico pharmacokinetic study demonstrated that the compound has a drug-like PK profile and good oral bioavailability.^154^ A 1,2,3-triazole–(thio)­barbituric acid derivative, (**Compound 14**), showed a 12.5-fold and 2.75-fold urease inhibition compared to hydroxyurea (IC_50_ of 100 ± 0.20 μM) and thiourea (IC_50_ of 22.0 ± 0.03 μM), respectively. Docking studies (PDB: 4H9M) revealed that a reduction in the flexibility of the flap region at the entrance of active site is essential for inhibiting the ureolytic activity. The in silico ADME profile showed that this compound is orally active and has no toxicity effect.^155^

### 5.4 Thiourea Derivatives

Thiourea (SC­(NH_2_)_2_), also known as thiocarbamide, is a multifunctional organosulfur compound, with its derivatives exhibiting various biological applications like antiviral, antibacterial, antifungal, anticonvulsant, anti-inflammatory, antitubercular, antithyroid, and anticancer properties.^174^ Derivatives from this class have been explored for their urease inhibition activity against *H. pylori* in which five compounds have shown potent urease inhibition (Table 4). The N-monosubstituted thiourea derivative (**Compound 15**), exhibited 170-fold more potency than standard acetohydroxamic acid (IC_50_ of 27.2 ± 0.7 μM). The docking studies (PDB: 1E9Y) revealed monosubstituted thiourea, benzene ring, and aceto and para positions of chlorine enhances the urease inhibition. The compound showed lower cell toxicity.^156^ An N-monosubstituted aroylthiourea (**Compound 16**), exhibited 450-fold and 543-fold more potency than standard acetohydroxamic acid (IC_50_ of 27.2 ± 0.7 μM) and thiourea (IC_50_ of 32.6 ± 0.9 μM), respectively. The compound was bound to the urea binding site with an exceptionally low *K*_D_ value of 0.420 ± 0.003 nM and a prolonged residence time of 6.7 min. The docking studies revealed that the thiourea moiety, *ortho*-nitro substituted benzene ring, and aceto moiety enhances the urease inhibition.^157^ An acetylphenol-based acyl thiourea derivative, (**Compound 17**), exhibited ∼413-fold more potent urease inhibition than thiourea (IC_50_ of 22.3 ± 0.031 μM). The docking studies (PDB: 3LA4) showed carbamothioyl, hydroxybenzene, 2-bromobenzamide, phenyl ring of bromobenzamide, and thiourea nitrogen are essential for activity. The in silico pharmacokinetic study revealed that this compound is safe.^158^ A thiourea moiety with lipophilic chain and phenyl residue derivative (**Compound 18**), showed potent urease inhibition of 1.7-fold than standard thiourea (IC_50_ value of 18.61 ± 0.11 μM). The docking studies (PDB: 4H9M) revealed that the aliphatic tail of this compound orienting toward the solvent and the bromo group at para position on benzene ring enhances the urease inhibition. The in silico ADME showed compound is safe to administer.^159^

### 5.5 Chromone Derivatives

#### 5.5.1 Flavonoid Derivatives

Flavonoids, a wide group of polyphenolic compounds with a benzo-γ-pyrone ring, are found in plants. They are known for their renowned health benefits, which include anti-inflammatory, antiviral, hepatoprotective, and antioxidant properties. Their biological activity depends on the structure, degree of hydroxylation, and functional group substitutions. In plants, flavonoids function as growth regulators and secondary antioxidants during biotic and abiotic stress.^175^ Flavonoid derivatives have gained attention as natural urease inhibitors for *H. pylori*. Here, we discuss seven flavonoid-based urease inhibitors (Table 4). A 4′,7,8-trihydroxyl-2-isoflavene (**Compound 19**), has shown 20-fold more potent urease inhibition than acetohydroxamic acid (IC_50_ value of 18.2 ± 1.6 μM). The docking study (PDB: 1E9Y) showed the isoflavene core, phenyl ring attached to position 2 of isoflavene, 7-hydroxyl, 8-hydroxyl moiety, and 4′-hydroxyl moiety essential for the urease inhibition.^160^ Quercetin (**Compound 20**), showed 1.76-fold increased urease inhibition than acetohydroxamic acid (IC_50_ value of 19.4 ± 2.0 μM). The docking studies (PDB: 1E9Y) showed 3-OH, 5-OH, and 3′,4′-dihydroxyl groups play a major role in the inhibition of *H. pylori* urease.^161^ It has low side effects and can be taken orally up to 1000 mg/d.^176,177^ Baicalin (**Compound 21**) and scutellarin (**Compound 22**), derived from *Scutellaria baicalensis*, have shown 0.17-fold and 0.30-fold increased urease inhibitory activity, respectively, than acetohydroxamic acid (IC_50_ value of 0.147 ± 0.05 mM). The docking study (PDB: 1E9Y) showed that these two compounds tightly anchored the flap region, preventing the flap from backing into its closed position. The 4′-hydroxyl group provides high binding affinity toward *H. pylori* urease. Baicalin and scutellarin have been reported to be safe with high LD_50_ values.^178,179^ Hesperetin-7-rhamnoglucoside (**Compound 23**), a bioflavonoid isolated from peel of *Citrus uranium* fruit has shown 2000-fold increase in urease inhibition compared to thiourea (IC_50_ value of 23.4 μM). The docking studies (PDB: 4HI0) revealed that hydroxyl group, aromatic ring, and alkyl substituents as essential for the activity.^163^ A hybrid of diosmin and thiourea (**Compound 24**), exhibited 1.8-fold increased urease inhibitory activity compared to standard thiourea which has IC_50_ value of 22.80 ± 0.011 μM. The substitution of nitro group at 3-position on aryl thiourea was responsible for potent urease inhibition. The docking study (PDB: 3LA4), showed good binding to the urease enzyme. The in silico ADMET study revealed the compound has drug-like profile and has good oral bioavailability.^164^ A hybrid of morin and thiourea (**Compound 25**), exhibited 2-fold increase in the urease inhibition compared to the control thiourea (IC_50_ value of 22.80 ± 0.011 μM). The anti-*H. pylori* activity showed a minimum inhibitory concentration (MIC) of 500 μg/mL with a zone of inhibition of 15 mm. The substitution of the bromo group and aryl thiourea enhanced the effectiveness of the compound in inhibiting the urease. The docking studies showed that the hydroxyl moiety, NH, and aromatic ring firmly fix the compound in the active site of the urease enzyme. The in silico ADMET study showed that this compound has favorable PK profile.^165^

#### 5.5.2 Catechol Derivatives

Catechol, are phenolic compounds that have shown antibacterial and antifungal activities.^180^ Researchers have used these as scaffolds to check their activity against the *H. pylori* urease enzyme (Table 4). A pyrogallol and catechol derivative (**Compound 26**), exhibited 11.4-fold increased urease inhibitory activity than acetohydroxamic acid (IC_50_ value of 17.2 ± 0.9 μM). The docking study (PDB: 1E9Y) showed a binding free energy of −11.48 kcal/mol revealing 2-hydroxyl moiety in A-ring and 4-hydroxyl moiety in B ring as responsible for inhibiting the urease activity.^166^ A catechol derivative (**Compound 27**), showed 0.398 probability of activity from prediction of activity spectra for substances test. The docking studies (PDB: 1E9Y) showed a docking score of −11.02 kcal/mol and revealed that catechol hydroxyls, carboxylic acid group, and aromatic C6 carbon are essential moieties for enhanced activity. The compound binds at the mouth of active site and also deep inside the cavity, thus inhibiting the enzyme.^167^

#### 5.5.3 Coumarin Derivatives

Coumarins (benzopyrones) and their derivatives are a diverse group of compounds found predominantly in plants, though they are present in animals and microorganisms. The study of coumarins dates back more than 200 years, with the first isolation of coumarin (simplest member), from *Coumarouna odorata* (*Dipteryx odorata*). Due to their structural versatility, they have gained significant attention for their diverse range of biological activities such as antimicrobial, anticancer, antiviral, anticoagulant, antioxidant, cardiovascular, anti-inflammatory, and CNS effects.^168^ Coumarins and their derivatives have also demonstrated *H. pylori* urease inhibitory activity. Jadhav et al., synthesized a few coumarin derivatives, among which **Compounds 28, 29, 30, 31, and 32** (Table 4) showed 0.8-fold, 0.9-fold, 0.82-fold, 0.8-fold, and -0.9-fold potent urease inhibition, respectively, than standard acetohydroxamic acid (IC_50_ value of 44.64 ± 0.36 μM). The experimental and docking data (PDB: 1E9Y) of *H. pylori* antiurease activity highlights that the presence of 4-, 5-, 7-, and/or 8-hydroxyl substitution and 4-phenyl group in benzenoid ring is essential to abolish the *H. pylori* urease activity.^169^ Coumarins has reported to be a safer molecule.^181^

#### 5.5.4 Chlorogenic Acid Derivatives

Chlorogenic acid, a naturally occurring polyphenolic compound, has garnered significant attention for its diverse biological activity such as anti-inflammatory, antioxidant, antimicrobial, anticarcinogenic, antihypertensive, antiviral, antihypercholesterolemia actions.^182^ Its role as a urease inhibitor has also been explored by researchers. A chlorogenic acid derivative (**Compound 33**) exhibited 2-fold increased urease inhibitory activity than standard thiourea (IC_50_ value of 22.80 ± 0.011 μM). The anti-*H. pylori* activity showed a zone of inhibition of 10.00 mm at an MIC of 500 μg/mL. The docking study (PDB: 3LA4) showed a docking score of −10.091 and a binding energy of −62.674 kcal/mol. It also showed that carbonyl, hydroxyl, and nitro group of the compound enhances the inhibition of urease (Table 4).^170^

From the above discussion, we can say that pharmacophoric features play an important role in identifying potent urease inhibitors. Pharmacophoric features define the key structural elements required for molecules to bind with the enzyme and enhance the inhibition. In the imidazole class of compounds, the linking of chloro substituted salicylic acid enhances the urease inhibition. In the hydroxamic class, compounds linking to the salicylic acid derivative, NH linkage, and carbon linkage and the substitution with a chloro or hydroxyl group increase the urease inhibition. Barbituric acids are also potent urease inhibitors. Substitution of a chloro or methyl group enhances the urease inhibitory activity. Aryolthiourea also plays a role in urease inhibition. Substituting electron-withdrawing groups such as chlorine or bromine on aryolthiourea enhances urease inhibition. The substitution of the hydroxyl moiety on catechol and coumarins will make the molecule effective in inhibiting the enzyme. Chlorogenic acid substituted with a chloro group also acts as urease inhibitors. Figure 7 depicts the pharmacophoric features responsible for urease inhibitory activity from various chemical classes.

**Figure 7:**
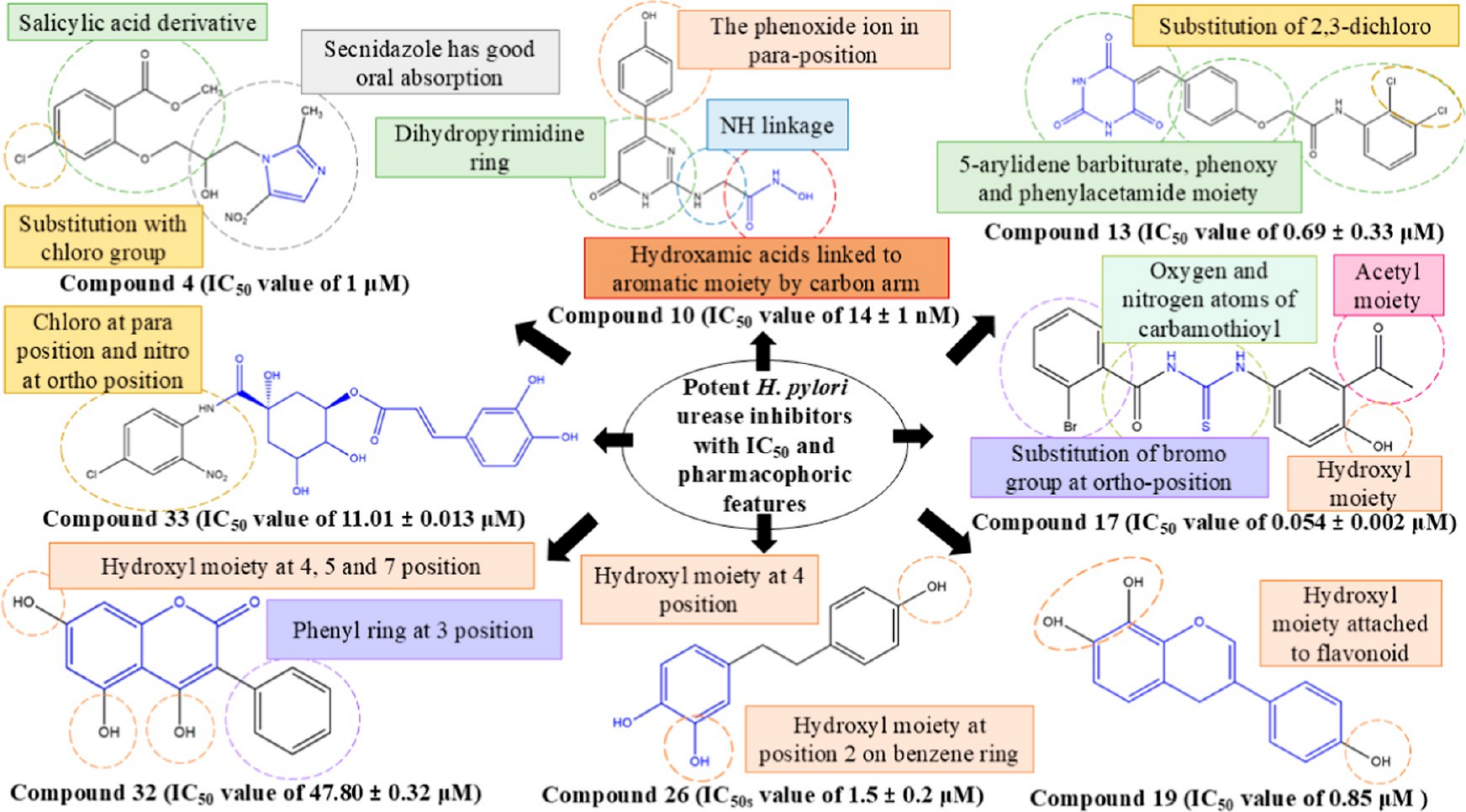
Pharmacophoric features of potent urease inhibitors of imidazole, hydroxamic acid, barbituric acid, thiourea, flavonoid, catechol, coumarin, and chlorogenic acid class.

##### 5.5.4.1 In Silico Evaluation of Promising Urease Inhibitors

Different PDB IDs can show variations in docking with a ligand due to differences in the resolution. These variations influence the binding affinity of the compound during molecular docking.^183^ Innovative approaches, such as reversible peptide tagging systems, have also been explored to regulate enzyme activity, offering alternative strategies for targeting urease.^184^ Therefore, to identify the best binding pose and interaction pattern, we performed molecular docking with the available PDB IDs of *H. pylori* urease on the most potent urease inhibitors identified from the available reports discussed in this manuscript. Accordingly, a docking study was performed on the available *H. pylori* urease PDB IDs such as 1E9Y, 6ZJA, and 6QSU using the Schrodinger suite (Maestro). We used Glide XP to identify compounds showing the best interaction. Among the selected eight compounds, **Compound 33**, **Compound 26**, and **Compound 10** from chlorogenic acid, catechol, and hydroxamic acid class, respectively, showed better docking score as compared to the other class of compounds (Figures 8, 9 and 10). The docking score and interactions of all the selected eight urease inhibitors in the active site residues are shown in Supporting Information Table S1. The interactions of these compounds in the active site of urease are given in Supporting Information Figures S21–S24. The docking study of **Compound 33** showed docking scores of −6.926, −8.145, and −8.767 against *H. pylori* urease PDB ID 1E9Y, 6ZJA, and 6QSU respectively. **Compound 26** belonging to pyrogallol and catechol showed docking scores of −6.578, −8.042, and −7.129 against *H. pylori* urease PDB ID 1E9Y, 6ZJA, and 6QSU, respectively. **Compound 10** belonging to the class hydroxamic acid showed docking scores of −7.228, −7.521, and −7.564 against *H. pylori* urease PDB ID 1E9Y, 6ZJA, and 6QSU respectively. The results show that difference in resolution affects the binding affinity of the compounds. These findings emphasize the importance of considering multiple PDB structures to ensure a more reliable and comprehensive assessment of inhibitor binding.

**Figure 8:**
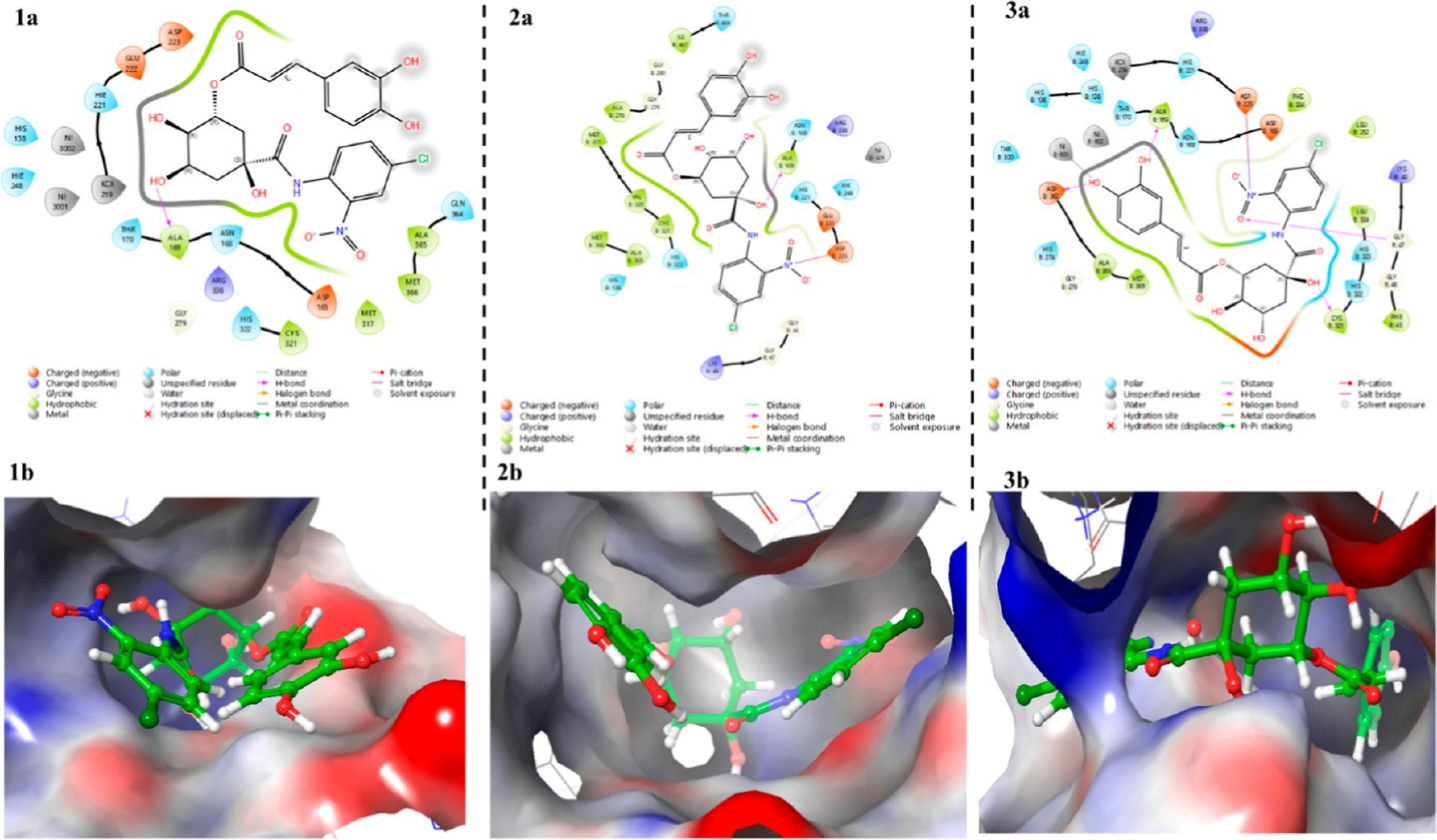
Binding mode of **Compound 33** in the active site of *H. pylori* urease: (1a) interactions of **Compound 33** with amino acid residues at the urease active site (PDB ID 1E9Y), (1b) 3D representation of **Compound 33** in the pocket of *H. pylori* urease (PDB ID 1E9Y), (2a) interactions of **Compound 33** with amino acid residues at the urease active site (PDB ID 6ZJA), (2b) 3D representation of **Compound 33** in the pocket of *H. pylori* urease (PDB ID 6ZJA), (3a) interactions of **Compound 33** with amino acid residues at the urease active site (PDB ID 6QSU), (3b) 3D representation of **Compound 33** in the pocket of *H. pylori* urease (PDB ID 6QSU).

**Figure 9:**
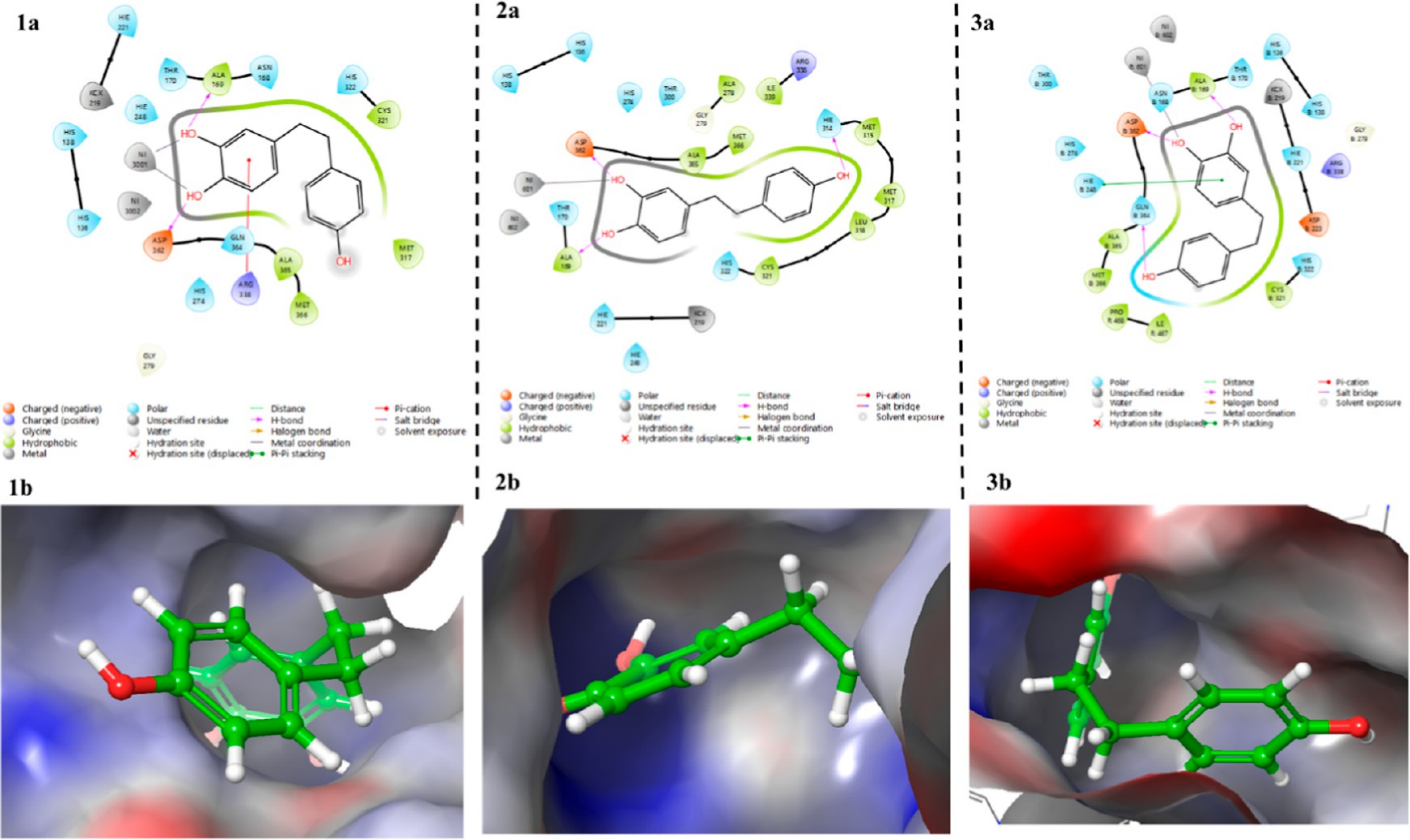
Binding mode of **Compound 26** in the active site of *H. pylori* urease: (1a) interactions of **Compound 26** with amino acid residues at urease active site (PDB ID 1E9Y), (1b) 3D representation of **Compound 26** in the pocket of *H. pylori* urease (PDB ID 1E9Y), (2a) interactions of **Compound 26** with amino acid residues at urease active site (PDB ID 6ZJA), (2b) 3D representation of **Compound 26** in the pocket of *H. pylori* urease (PDB ID 6ZJA), (3a) interactions of **Compound 26** with amino acid residues at urease active site (PDB ID 6QSU), (3b) 3D representation of **Compound 26** in the pocket of *H. pylori* urease (PDB ID 6QSU).

**Figure 10:**
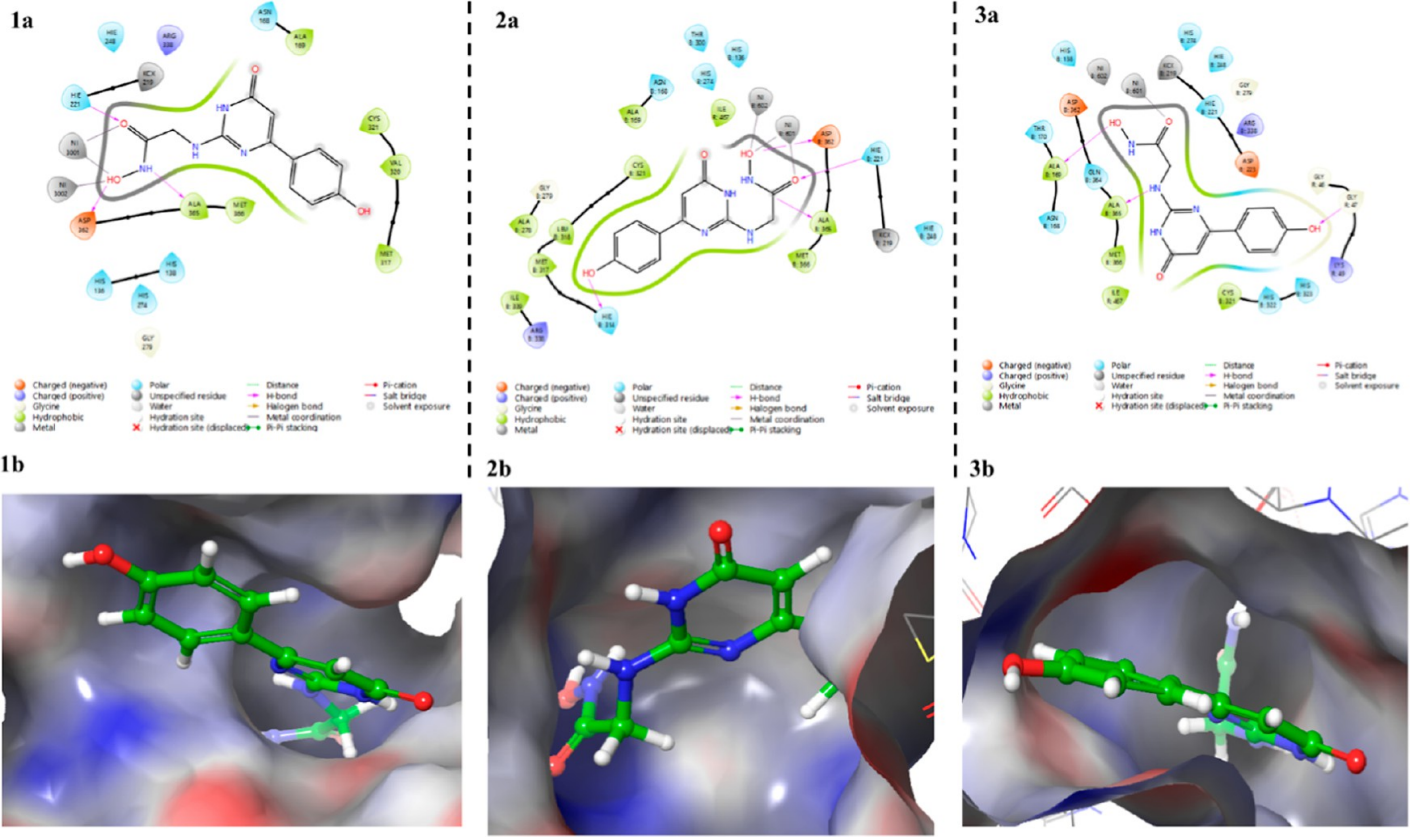
Binding mode of **Compound 10** in the active site of *H. pylori* urease: (1a) interactions of **Compound 10** with amino acid residues at the urease active site (PDB ID 1E9Y), (1b) 3D representation of **Compound 10** in the pocket of *H. pylori* urease (PDB ID 1E9Y), (2a) interactions of **Compound 10** with amino acid residues at the urease active site (PDB ID 6ZJA), (2b) 3D representation of **Compound 10** in the pocket of *H. pylori* urease (PDB ID 6ZJA), (3a) interactions of **Compound 10** with amino acid residues at the urease active site (PDB ID 6QSU), (3b) 3D representation of **Compound 10** in the pocket of *H. pylori* urease (PDB ID 6QSU).

## 6 Conclusions

The supramolecular assembly of *H. pylori* urease is crucial in enabling the bacteria to survive the gastric acidic condition. This enzyme is also involved in the processes of angiogenesis and platelet aggregation, leading to pathogenesis. A thorough analysis of urease enzyme as discussed in this review article, clearly points out that the urease enzyme could be a potential target for effectively eradicating *H. pylori*. Synthetic and natural compounds such as imidazole, hydroxamic acids, barbituric acids, thiourea, flavonoids, coumarins, catechol, and chlorogenic acid have shown potent urease inhibitory activity. A detailed review of the chemistry of these compounds showed that the pharmacophoric features play an important role in identifying potent urease inhibitors. By using pharmacophore modeling, virtual screening, and molecular docking, extensive libraries of chemicals can be effectively screened to find candidates with a good optimal binding affinity and good interaction patterns with *H. pylori* urease. Optimizing pharmacokinetic characteristics such as half-life, gastric stability, and oral bioavailability is also necessary, especially for drugs that act in the acidic environment of stomach. The molecular docking study that we performed showed that compounds belonging to the classes of chlorogenic acid, catechol, and hydroxamic acid have better docking scores than the other classes of reported compounds. It is essential to perform pharmacophore-based screening focusing specifically on the above three scaffolds to identify a promising urease inhibitor that is safe and effective and possesses necessary pharmacokinetic features. Considering the multifaceted role of the urease enzyme in the survival and pathogenesis of *H. pylori*, it could be concluded that the discovery of a promising urease inhibitor will revolutionize the *H. pylori* treatment. The inclusion of urease inhibitors as an adjuvant with existing antibiotic therapies could help in overcoming many of the existing treatment challenges in eradicating the organism from the affected individual. It can contribute to overcoming antibiotic resistance, a growing challenge worldwide. Furthermore, it may reduce the treatment duration and improve patient compliance, contributing to better clinical outcomes. There is also a future scope in discovering molecules that can inhibit urease genes discussed in this perspective and prevent their expression.

## Supplementary Material



## Notes

Docking score and interactions of potent compounds; and interactions of **Compounds 4, 10, 13, 17, 19, 26, 32, and 33** with amino acid residues at urease active site (PDF)

## References

[1] Malfertheiner P., Camargo M. C., El-Omar E., Liou J. M., Peek R., Schulz C., Smith S. I., Suerbaum S. (2023). *Helicobacter
pylori* infection. Nat. Rev. Dis. Primers.

[2] Krzyżek P., Gos’ciniak G. (2018). Morphology
of Helicobacter pylori as a result of peptidoglycan
and cytoskeleton rearrangements. Przegl. Gastroenterol..

[3] Sipponen P., Kosunen T. U., Valle J., Riihelä M., Seppälä K. (1992). Helicobacter pylori
infection and
chronic gastritis in gastric cancer. J. Clin.
Pathol..

[4] Mhaskar R. S., Ricardo I., Azliyati A., Laxminarayan R., Amol B., Santosh W., Boo K. (2013). Assessment
of risk
factors of helicobacter pylori infection and peptic ulcer disease. J. Global Infect. Dis..

[5] Kim J. S., Chung S. J., Choi Y. S., Cheon J. H., Kim C. W., Kim S. G., Jung H. C., Song I. S. (2007). *Helicobacter
pylori* eradication for low-grade gastric mucosa-associated
lymphoid tissue lymphoma is more successful in inducing remission
in distal compared to proximal disease. Br.
J. Cancer.

[6] Eradication of Helicobacter pylori for non-ulcer dyspepsia. Cochrane Database of Systematic Reviews. https://www.cochranelibrary.com/cdsr/doi/10.1002/14651858.CD002096.pub2/abstract, accessed on February 12, 2025.

[7] Lin K. D., Chiu G. F., Waljee A. K., Owyang S. Y., El-Zaatari M., Bishu S., Grasberger H., Zhang M., Wu D. C., Kao J. Y. (2019). Effects of anti-*Helicobacter pylori* therapy on incidence of autoimmune diseases, including inflammatory
bowel diseases. Clin. Gastroenterol. Hepatol..

[8] Polat F. R., Polat S. (2012). The Effect of Helicobacter
pylori on Gastroesophageal Reflux Disease. J.
Soc. Laparoendosc. Surg..

[9] Basyigit S., Aktas B., Simsek H., Kucukazman M. (2014). Diffuse intestinal
nodular lymphoid hyperplasia in an immunoglobulin-A-deficient patient
with *Helicobacter pylori* infection. Endoscopy.

[10] Alqutub A. N., Masoodi I. (2010). A case of gastric polyposis
in antral area of stomach
following prolonged proton-pump therapy. Ger.
Med. Sci..

[11] Kouitcheu
Mabeku L. B., Noundjeu Ngamga M. L., Leundji H. (2018). Potential risk factors
and prevalence of *Helicobacter pylori* infection among
adult patients with dyspepsia symptoms in Cameroon. BMC Infect. Dis..

[12] Öztekin M., Yılmaz B., Ağagündüz D., Capasso R. (2021). Overview of *Helicobacter pylori* Infection:
Clinical Features, Treatment, and Nutritional Aspects. Diseases.

[13] Jaime F., Villagra’n A., Herna’ndez C., Ortiz M., Serrano C., Harris P. R. (2018). Functional
gastrointestinal disorders in children from
low socio-economic status and *Helicobacter pylori* infection. Child Care Health Dev..

[14] Brenner H., Arndt V., Stürmer T., Stegmaier C., Ziegler H., Dhom G. (2000). Individual and joint
contribution
of family history and *Helicobacter pylori* infection
to the risk of gastric carcinoma. Cancer.

[15] Boehnke K. F., Brewster R. K., Sa’nchez B. N., Valdivieso M., Bussalleu A., Guevara M., Saenz C. G., Alva S. O., Gil E., Xi C. (2018). An assessment of drinking
water contamination with *Helicobacter pylori* in Lima,
Peru. Helicobacter.

[16] Ogihara A., Kikuchi S., Hasegawa A., Kurosawa M., Miki K., Kaneko E., Mizukoshi H. (2000). Relationship between *Helicobacter
pylori* infection and smoking and drinking habits. J. Gastroenterol. Hepatol..

[17] Malaty H. M., Graham D. Y., Klein D. G., Adam E. E., Evans D. J. (1991). Transmission
of Helicobacter pylori infection studies in families of healthy individuals. Scand. J. Gastroenterol..

[18] Amaral O., Fernandes I., Veiga N., Pereira C., Chaves C., Nelas P., Silva D. (2017). Living Conditions and *Helicobacter
pylori* in Adults. BioMed Res. Int..

[19] Liu Y., Lin H., Bai Y., Qin X., Zheng X., Sun Y., Zhang Y. (2008). Study on the relationship between *Helicobacter
pylori* in the dental plaque and the occurrence of dental
caries or oral
hygiene index. Helicobacter.

[20] Monno R., De Laurentiis V., Trerotoli P., Roselli A. M., Ierardi E., Portincasa P. (2019). *Helicobacter
pylori* infection: association
with dietary habits and socioeconomic conditions. Clin. Res. Hepatol. Gastroenterol..

[21] Katelaris P., Hunt R., Bazzoli F., Cohen H., Fock K. M., Gemilyan M., Malfertheiner P., Me’graud F., Piscoya A., Quach D., Vakil N., Vaz Coelho L. G., LeMair A., Melberg J. (2023). *Helicobacter
pylori* World Gastroenterology Organization Global Guideline. J. Clin. Gastroenterol..

[22] Emmanuel B. N., Peter D. A., Peter M. O., Adedayo I. S., Olaifa K. (2025). *Helicobacter
pylori* infection in Africa: comprehensive insight into its
pathogenesis, management, and future perspectives. J. Umm Al-Qura Univ. Appl. Sci..

[23] Fock K. M., Ang T. L. (2010). Epidemiology of *Helicobacter
pylori* infection and gastric cancer in Asia. J. Gastroenterol.
Hepatol..

[24] Zamani M., Ebrahimtabar F., Zamani V., Miller W. H., Alizadeh-Navaei R., Shokri-Shirvani J., Derakhshan M. H. (2018). Systematic review with meta-analysis:
the worldwide prevalence of *Helicobacter pylori* infection. Aliment. Pharmacol. Ther..

[25] Chandra, V. Indian Youth Population: Socio-Demographic Characteristics. In Handbook of the Sociology of Youth in BRICS Countries; Dwyer, T. , Gorshkov, M. K. , Modi, I. , Li, C. , Mapadimeng, M. S. , Eds.; World Scientific: Singapore, 2018; pp 145–165.

[26] Georgopoulos S. D., Papastergiou V., Karatapanis S. (2013). Current options for the treatment
of *Helicobacter pylori*. Expert
Opin. Pharmacother..

[27] Suzuki S., Kusano C., Horii T., Ichijima R., Ikehara H. (2022). The Ideal *Helicobacter pylori* Treatment
for the Present and the Future. Digestion.

[28] Sugano K., Tack J., Kuipers E. J., Graham D. Y., El-Omar E. M., Miura S., Haruma K., Asaka M., Uemura N., Malfertheiner P. (2015). Kyoto global
consensus report on *Helicobacter
pylori* gastritis. Gut.

[29] Fock K. M., Talley N., Moayyedi P., Hunt R., Azuma T., Sugano K., Xiao S. D., Lam S. K., Goh K. L., Chiba T., Uemura N., Kim J. G., Kim N., Ang T. L., Mahachai V., Mitchell H., Rani A. A., Liou J. M., Vilaichone R., Sollano J. (2008). Asia-Pacific consensus
guidelines on gastric cancer prevention. J.
Gastroenterol. Hepatol..

[30] Fujioka T., Yoshiiwa A., Okimoto T., Kodama M., Murakami K. (2007). Guidelines
for the management of *Helicobacter pylori* infection
in Japan: current status and future prospects. J. Gastroenterol..

[31] Song M., Ang T. L. (2014). Second and third line treatment options for *Helicobacter pylori* eradication. World
J. Gastroenterol..

[32] Zagari R. M., Rabitti S., Eusebi L. H., Bazzoli F. (2018). Treatment of *Helicobacter pylori* infection: A clinical practice update. Eur. J. Clin. Invest..

[33] Lin T.-F., Hsu P.-I. (2018). Second-line rescue treatment of *Helicobacter
pylori* infection: Where are we now?. World J. Gastroenterol..

[34] Gupta A., Shetty S., Mutalik S., Chandrashekar
HMathewJhaMishra R. E. M. A. B., Rajpurohit S., Ravi G., Saha M., Moorkoth S., Rajpurohit S., Ravi G., Saha M., Moorkoth S. (2023). Treatment
of *H. pylori* infection and gastric ulcer: Need for
novel Pharmaceutical formulation. Heliyon.

[35] Krishnamurthy P., Parlow M., Zitzer J. B., Vakil N. B., Mobley H. L. T., Levy M., Phadnis S. H., Dunn B. E. (1998). *Helicobacter
pylori* Containing Only Cytoplasmic Urease Is Susceptible
to Acid. Infect. Immun..

[36] Mobley H. (1996). The role of
Helicobacter pylori urease in the pathogenesis of gastritis and peptic
ulceration. Aliment. Pharmacol. Ther..

[37] Schoep T. D., Fulurija A., Good F., Lu W., Himbeck R. P., Schwan C., Choi S. S., Berg D. E., Mittl P. R. E., Benghezal M., Marshall B. J. (2010). Surface properties
of Helicobacter
pylori urease complex are essential for persistence. PLoS One.

[38] Scott D. R., Marcus E. A., Weeks D. L., Sachs G. (2002). Mechanisms
of acid
resistance due to the urease system of *Helicobacter pylori*. Gastroenterology.

[39] Kao C. Y., Sheu B. S., Wu J. J. (2016). Helicobacter pylori
infection: An
overview of bacterial virulence factors and pathogenesis. Biomed. J..

[40] Sgouras D. N., Trang T. T. H., Yamaoka Y. (2015). Pathogenesis of Helicobacter pylori
Infection. Helicobacter.

[41] Mobley H. L. T., Hu L. T., Foxall P. A. (1991). Helicobacter pylori Urease: Properties
and Role in Pathogenesis. Scand. J. Gastroenterol..

[42] Turbett G. R., Nandapalan N., Campbell I. G., Nikoletti S. M., Mee B. J. (1991). Characterisation
of the urease from *Helicobacter
pylori* and comparison with the ureases from related spiral
gastric bacteria. FEMS Microbiol. Immunol..

[43] Mobley, H. L. T. Urease. In Helicobacter pylori: Physiology and Genetics; Mobley, H. L. , Mendz, G. L. , Hazell, S. L. , Eds.; ASM Press: WA, 2001; pp 177–191. 21290711

[44] Rokita E., Makristathis A. (2001). Genetic complementation of the urease-negative *Helicobacter pylori* mutant N6ureB::TnKm. FEMS Immunol. Med. Microbiol..

[45] Ha N. C., Oh S. T., Sung J. Y., Cha K. A., Lee M. H., Oh B.-H. (2001). Supramolecular assembly
and acid resistance of *Helicobacter
pylori* urease. Nat. Struct. Mol. Biol..

[46] Hu L. T., Foxall P. A., Russell R., Mobley H. L. (1992). Purification of
recombinant Helicobacter pylori urease apoenzyme encoded by ureA and
ureB. Infect. Immun..

[47] Biagi F., Musiani F., Ciurli S. (2013). Structure
of the UreD-UreF-UreG-UreE
complex in *Helicobacter pylori*: a model study. J. Biol. Inorg. Chem..

[48] Bellucci M., Zambelli B., Musiani F., Turano P., Ciurli S. (2009). *Helicobacter
pylori* UreE, a urease accessory protein: specific Ni^2+^- and Zn^2+^-binding properties and interaction
with its cognate UreG. Biochem. J..

[49] Masetti M., Falchi F., Gioia D., Recanatini M., Ciurli S., Musiani F. (2020). Targeting the protein
tunnels of
the urease accessory complex: a theoretical investigation. Molecules.

[50] Fong Y. H., Wong H. C., Chuck C. P., Chen Y. W., Sun H., Wong K. B. (2011). Assembly of preactivation
complex for urease maturation
in Helicobacter pylori: crystal structure of UreF-UreH protein complex. J. Biol. Chem..

[51] Maier R. J., Benoit S. L., Seshadri S. (2007). Nickel-binding and accessory proteins
facilitating Ni-enzyme maturation in *Helicobacter pylori*. BioMetals.

[52] Skouloubris S., Thiberge J. M., Labigne A., De Reuse H. (1998). The *Helicobacter
pylori* UreI protein is not involved in urease activity but
is essential for bacterial survival *in vivo*. Infect. Immun..

[53] Mehta N., Olson J. W., Maier R. J. (2003). Characterization
of Helicobacter
pylori nickel metabolism accessory proteins needed for maturation
of both urease and hydrogenase. J. Bacteriol..

[54] Cussac V., Ferrero R. L., Labigne A. (1992). Expression
of Helicobacter Pylori
urease genes in Escherichia Coli grown under nitrogen-limiting conditions. J. Bacteriol..

[55] Valenzuela M., Cerda O., Toledo H. (2003). Overview on
chemotaxis and acid resistance
in Helicobacter pylori. Biol. Res..

[56] Stingl K., De Reuse H. (2005). Staying alive overdosed:
how does *Helicobacter
pylori* control urease activity?. Int.
J. Med. Microbiol..

[57] Rektorschek M., Buhmann A., Weeks D., Schwan D., Bensch K. W., Eskandari S., Scott D., Sachs G., Melchers K. (2000). Acid resistance
of *Helicobacter pylori* depends on the UreI membrane
protein and an inner membrane proton barrier. Mol. Microbiol..

[58] Weeks D. L., Eskandari S., Scott D. R., Sachs G. (2000). A H+-gated urea channel:
the link between Helicobacter pylori urease and gastric colonization. Science.

[59] Mulrooney S. B., Hausinger R. P. (2003). Nickel uptake and utilization by
microorganisms. FEMS Microbiol. Rev..

[60] Vannini A., Pinatel E., Costantini P. E., Pelliciari S., Roncarati D., Puccio S., De Bellis G., Peano C., Danielli A. (2017). Comprehensive mapping of the *Helicobacter pylori* NikR regulon provides new insights in
bacterial nickel responses. Sci. Rep..

[61] Fischer F., Robbe-Saule M., Turlin E., Mancuso F., Michel V., Richaud P., Veyrier F. J., De Reuse H., Vinella D. (2016). Characterization
in *Helicobacter pylori* of a Nickel Transporter Essential
for Colonization That Was Acquired during Evolution by Gastric Helicobacter
Species. PLoS Pathog..

[62] Abraham L. O., Li Y., Zamble D. B. (2006). The metal-
and DNA-binding activities of *Helicobacter
pylori* NikR. J. Inorg. Biochem..

[63] Muller C., Bahlawane C., Aubert S., Delay C. M., Schauer K., Michaud-Soret I., De Reuse H. (2011). Hierarchical regulation of the NikR-mediated
nickel response in *Helicobacter pylori*. Nucleic Acids Res..

[64] Chivers P. T., Sauer R. T. (1999). NikR is a ribbon-helix-helix DNA-binding protein. Protein Sci..

[65] Chivers P. T., Sauer R. T. (2002). NikR repressor: high-affinity nickel binding to the
C-terminal domain regulates binding to operator DNA. Chem. Biol..

[66] Carrington P. E., Chivers P. T., Al-Mjeni F., Sauer R. T., Maroney M. J. (2003). Nickel
coordination is regulated by the DNA-bound state of NikR. Nat. Struct. Mol. Biol..

[67] Nolan K. J., McGee D. J., Mitchell H. M., Kolesnikow T., Harro J. M., O’Rourke J., Wilson J. E., Danon S. J., Moss N. D., Mobley H. L. T., Lee A. (2002). *In vivo* behavior of a *Helicobacter pylori* SS1 nixA mutant
with reduced urease activity. Infect. Immun..

[68] Fulkerson J. F., Garner R. M., Mobley H. L. T. (1998). Conserved
residues and motifs in
the NixA protein of *Helicobacter Pylori* are critical
for the high affinity transport of nickel ions. J. Biol. Chem..

[69] Mobley H. L. T., Garner R. M., Bauerfeind P. (1995). *Helicobacter pylori* nickel-transport gene nixA: synthesis of catalytically active urease
in *Escherichia coli* independent of growth conditions. Mol. Microbiol..

[70] Yang X., Li H., Cheng T., Xia W., Lai Y. T., Sun H. (2014). Nickel translocation
between metallochaperones HypA and UreE in *Helicobacter pylori*. Metallomics.

[71] Sachs, G. ; Scott, D. R. ; Weeks, D. L. ; Rektorscheck, M. ; Melchers, K. Regulation of Urease for Acid Habitation. In Helicobacter pylori: Physiology and Genetics; Mobley, H. L. , Mendz, G. L. , Hazell, S. L. , Eds.; ASM Press: WA, 2001; pp 277–283. 21290728

[72] Brito B. B. d., Silva F. A. F. d., Soares A. S., Pereira V. A., Santos M. L. C., Sampaio M. M., Neves P. H. M., Melo F. F. d. (2019). Pathogenesis
and clinical management of *Helicobacter pylori* gastric
infection. World J. Gastroenterol..

[73] Sachs G., Weeks D. L., Wen Y., Marcus E. A., Scott D. R., Melchers K. (2005). Acid Acclimation by *Helicobacter pylori*. Physiology.

[74] Dunne C., Dolan B., Clyne M. (2014). Factors that
mediate colonization
of the human stomach by *Helicobacter pylori*. World J. Gastroenterol..

[75] Debowski A. W., Walton S. M., Chua E. G., Tay A. C. Y., Liao T., Lamichhane B., Himbeck R., Stubbs K. A., Marshall B. J., Fulurija A., Benghezal M. (2017). *Helicobacter
pylori* gene silencing *in vivo* demonstrates
urease is essential
for chronic infection. PLoS Pathog..

[76] Eaton K. A., Brooks C. L., Morgan D. R., Krakowka S. (1991). Essential role of urease
in pathogenesis of gastritis induced by Helicobacter pylori in gnotobiotic
piglets. Infect. Immun..

[77] Olivera-Severo D., Uberti A. F., Marques M. S., Pinto M. T., Gomez-Lazaro M., Figueiredo C., Leite M., Carlini C. R. (2017). A New Role for *Helicobacter
pylori* Urease: Contributions to Angiogenesis. Front. Microbiol..

[78] Tsujii M., Kawano S., Tsuji S., Fusamoto H., Kamada T., Sato N. (1992). Mechanism of gastric
mucosal damage induced by ammonia. Gastroenterology.

[79] Zhao S. Q., Zheng H. L., Zhong X. T., Wang Z. Y., Su Y., Shi Y. Y. (2024). Effects and mechanisms
of *Helicobacter pylori* infection on the occurrence
of extra-gastric tumors. World J. Gastroenterol..

[80] Uberti A. F., Olivera-Severo D., Wassermann G. E., Scopel-Guerra A., Moraes J. A., Barcellos-de-Souza P., Barja-Fidalgo C., Carlini C. R. (2013). Pro-inflammatory properties and neutrophil
activation
by *Helicobacter pylori* urease. Toxicon.

[81] Valenzuela-Valderrama M., Cerda-Opazo P., Backert S., Gonza’lez M. F., Carrasco-Ve’liz N., Jorquera-Cordero C., Wehinger S., Canales J., Bravo D., Quest A. F. G. (2019). The *Helicobacter Pylori* Urease Virulence
Factor Is Required
for the Induction of Hypoxia-Induced Factor-1α in Gastric Cells. Cancers.

[82] Baj J., Forma A., Sitarz M., Portincasa P., Garruti G., Krasowska D., Maciejewski R. (2021). *Helicobacter
Pylori* Virulence Factors—Mechanisms of Bacterial Pathogenicity
in the Gastric Microenvironment. Cells.

[83] Scridon A. (2022). Platelets
and Their Role in Hemostasis and Thrombosis—From Physiology
to Pathophysiology and Therapeutic Implications. Int. J. Mol. Sci..

[84] Holinstat M. (2017). Normal Platelet
Function. Cancer Metastasis Rev..

[85] Scopel-Guerra A., Olivera-Severo D., Staniscuaski F., Uberti A. F., Callai-Silva N., Jaeger N., Porto B. N., Carlini C. R. (2017). The Impact of *Helicobacter pylori* Urease upon Platelets and Consequent
Contributions to Inflammation. Front. Microbiol..

[86] Wassermann G. E., Olivera-Severo D., Uberti A. F., Carlini C. R. (2010). *Helicobacter
pylori* urease activates blood platelets through a lipoxygenase-mediated
pathway. J. Cell. Mol. Med..

[87] Byrne M. F., Kerrigan S. W., Corcoran P. A., Atherton J. C., Murray F. E., Fitzgerald D. J., Cox D. M. (2003). *Helicobacter pylori* binds von Willebrand
factor and interacts with GPIb to induce platelet
aggregation. Gastroenterology.

[88] Yeh J. J., Tsai S., Wu D. C., Wu J. Y., Liu T. C., Chen A. (2010). P-selectin-dependent
platelet aggregation and apoptosis may explain
the decrease in platelet count during *Helicobacter pylori* infection. Blood.

[89] Kountouras J., Gavalas E., Zavos C., Stergiopoulos C., Chatzopoulos D., Kapetanakis N., Gisakis D. (2007). Alzheimer’s
disease and *Helicobacter pylori* infection: defective
immune regulation and apoptosis as proposed common links. Med. Hypotheses.

[90] Malaguarnera M., Bella R., Alagona G., Ferri R., Carnemolla A., Pennisi G. (2004). *Helicobacter pylori* and Alzheimer’s
disease: a possible link. Eur. J. Intern. Med..

[91] Uberti A. F., Callai-Silva N., Grahl M. V. C., Piovesan A. R., Nachtigall E. G., Furini C. R. G., Carlini C. R. (2022). *Helicobacter pylori* Urease: Potential Contributions to Alzheimer’s Disease. Int. J. Mol. Sci..

[92] Adlimoghaddam A., Sabbir M. G., Albensi B. C. (2016). Ammonia
as a potential neurotoxic
factor in Alzheimer’s disease. Front.
Mol. Neurosci..

[93] Palacios E., Lobos-Gonza’lez L., Guerrero S., Kogan M. J., Shao B., Heinecke J. W., Quest A. F. G., Leyton L., Valenzuela-Valderrama M. (2023). *Helicobacter
pylori* outer membrane vesicles induce astrocyte reactivity
through nuclear
factor-kappa B activation and cause neuronal damage *in vivo* in a murine model. J. Neuroinflammation.

[94] Wang F., Yao Z., Jin T., Mao B., Shao S., Shao C. (2024). Research progress
on Helicobacter pylori infection related neurological diseases. Ageing Res. Rev..

[95] Jiang H. X., Qin S. Y., Min Z., Xie M. Z., Lin T., Hu B. L., Guo X. Y. (2013). Association
of Helicobacter pylori
with elevated blood ammonia levels in cirrhotic patients: a meta-analysis. Yonsei Med. J..

[96] Elhadidy A. A., Basiouny M. A. (2021). Evaluation of *Helicobacter
pylori* infection
as a potential risk factor of acute ischemic cerebrovascular stroke. Alexandria J. Med..

[97] Wang Z. W., Li Y., Huang L. Y., Guan Q. K., Xu D. W., Zhou W. K., Zhang X. Z. (2012). *Helicobacter
pylori* infection contributes
to high risk of ischemic stroke: evidence from a meta-analysis. J. Neurol..

[98] Knopman D. S., Amieva H., Petersen R. C., Che’telat G., Holtzman D. M., Hyman B. T., Nixon R. A., Jones D. T. (2021). Alzheimer
Disease. Nat. Rev. Dis. Primers.

[99] Chmiela M., Walczak N., Rudnicka K. (2018). *Helicobacter
pylori* outer membrane vesicles involvement in the infection
development
and *Helicobacter pylori*-related diseases. J. Biomed. Sci..

[100] Park A. M., Tsunoda I. (2022). *Helicobacter
pylori* infection in the stomach induces neuroinflammation:
the potential
roles of bacterial outer membrane vesicles in an animal model of Alzheimer’s
Disease. Inflammation Regener..

[101] Roszczenko-Jasin’ska P., Wojtys’ M. I., Jagusztyn-Krynicka E. K. (2020). *Helicobacter pylori* treatment in the post-antibiotics era—searching for new drug
targets. Appl. Microbiol. Biotechnol..

[102] Gisbert J. P. (2005). The recurrence of *Helicobacter
pylori* Infection: incidence and variables influencing it.
A critical review. Am. J. Gastroenterol..

[103] Garza-Gonza’lez E., Perez-Perez G. I., Maldonado-Garza H. J., Bosques-Padilla F. J. (2014). A review of *Helicobacter
pylori* diagnosis, treatment, and methods to detect eradication. World J. Gastroenterol..

[104] Hooi J. K. Y., Lai W. Y., Ng W. K., Suen M. M. Y., Underwood F. E., Tanyingoh D., Malfertheiner P., Graham D. Y., Wong V. W. S., Wu J. C. Y., Chan F. K. L., Sung J. J. Y., Kaplan G. G., Ng S. C. (2017). Global Prevalence
of *Helicobacter pylori* Infection: Systematic Review
and Meta-Analysis. Gastroenterology.

[105] Graham D. Y. (1998). Antibiotic resistance in *Helicobacter Pylori*: implications for therapy. Gastroenterology.

[106] Savoldi A., Carrara E., Graham D. Y., Conti M., Tacconelli E. (2018). Prevalence of antibiotic resistance in *Helicobacter
pylori*: A systematic review and meta-analysis in World Health
Organization regions. Gastroenterology.

[107] Corte’s P., Nelson A. D., Bi Y., Stancampiano F. F., Murray L. P., Pujalte G. G. A., Gomez V., Harris D. M. (2021). Treatment
Approach of Refractory *Helicobacter pylori* Infection:
A Comprehensive Review. J. Prim. Care Community
Health.

[108] Sarem M., Corti R. (2016). Role of *Helicobacter
pylori* coccoid forms in infection and recrudescence. Gastroenterol. Hepatol..

[109] Faghri J., Poursina F., Moghim S., Zarkesh
Esfahani H., Nasr Esfahani B., Fazeli H., Mirzaei N., Jamshidian A., Ghasemian Safaei H. (2014). Morphological and Bactericidal Effects
of Different Antibiotics on Helicobacter Pylori. Jundishapur J. Microbiol..

[110] Berry V., Jennings K., Woodnutt G. (1995). Bactericidal and morphological
effects of amoxicillin on Helicobacter pylori. Antimicrob. Agents Chemother..

[111] Varbanova M., Malfertheiner P. (2011). Bacterial load and degree of gastric
mucosal inflammation in Helicobacter pylori infection. Dig. Dis..

[112] Belda S., Saez J., Santiba’ñez M., Rodri’guez J. C., Sola-Vera J., Ruiz-Garci’a M., Brotons A., Lo’pez-Girona E., Pe’rez E., Sillero C., Royo G. (2012). Relationship between
bacterial load,
morbidity and cagA gene in patients infected by *Helicobacter
pylori*. Clin. Microbiol. Infect..

[113] Lai Y. C., Wang T. H., Huang S. H., Yang S. S., Wu C. H., Chen T. K., Lee C. L. (2003). Density
of *Helicobacter pylori* may affect the efficacy of
eradication
therapy and ulcer healing in patients with active duodenal ulcers. World J. Gastroenterol..

[114] Cammarota G., Branca G., Ardito F., Sanguinetti M., Ianiro G., Cianci R., Torelli R., Masala G., Gasbarrini A., Fadda G., Landolfi R., Gasbarrini G. (2010). Biofilm Demolition
and Antibiotic Treatment to Eradicate Resistant *Helicobacter
Pylori*: A Clinical Trial. Clin. Gastroenterol.
Hepatol..

[115] Cammarota G., Sanguinetti M., Gallo A., Posteraro B. (2012). Review article:
biofilm formation by *Helicobacter pylori* as a target
for eradication of resistant infection. Aliment.
Pharmacol. Ther..

[116] Garci’a A., Salas-Jara M. J., Herrera C., Gonza’lez C. (2014). Biofilm and *Helicobacter
pylori*: From environment to human host. World J. Gastroenterol..

[117] Hathroubi S., Servetas S. L., Windham I., Merrell D. S., Ottemann K. M. (2018). *Helicobacter pylori* Biofilm Formation
and Its Potential Role in Pathogenesis. Microbiol.
Mol. Biol. Rev..

[118] Rocha G. R., Lemos F. F. B., Silva L. G. d. O., Luz M. S., Correa Santos G. L., Rocha Pinheiro S. L., Calmon M. S., de Melo F. F. (2025). Overcoming antibiotic-resistant *Helicobacter pylori* infection: Current challenges and emerging
approaches. World J. Gastroenterol..

[119] Hou C., Yin F., Wang S., Zhao A., Li Y., Liu Y. (2022). *Helicobacter
pylori* Biofilm-Related Drug Resistance
and New Developments in Its Anti-Biofilm Agents. Infect. Drug Resist..

[120] Wu X., Wu D., Cui G., Lee K. H., Yang T., Zhang Z., Liu Q., Zhang J., Chua E. G., Chen Z. (2024). Association Between
Biofilm Formation and Structure and Antibiotic
Resistance in *H. pylori*. Infect.
Drug Resist..

[121] Mohiuddin N., Hajree S. A., Fatima R., Akmal M. M., Hassan S. I., Rasheed A., Khan A. A. (2021). Comparison
of Once-Daily
Triple Therapy versus Conventional Triple Therapy for Patient Compliance
and the Eradication of Helicobacter Pylori Infection. Int. J. Med. Res. Health Sci..

[122] Huguet J. M., Ferrer-Barcelo’ L., Sua’rez P., Barcelo-Cerda S., Sempere J., Saracino I. M., Fiorini G., Vaira D., Pe’rez-Ai’sa A’., Jonaitis L., Tepes B., Castro-Fernandez M., Pabo’n-Carrasco M., Keco-Huerga A., Voynovan I., Lucendo A. J., Lanas A. ’., Marti’nez-Domi’nguez S. J., Alfaro Almajano E., Rodrigo L., Vologzanina L., Bordin D. S., Gasbarrini A., Babayeva G., Lerang F., Leja M., Kupčinskas J., Rokkas T., Marcos-Pinto R., Meštrovic’ A., Gridnyev O., Phull P. S., Smith S. M., Boltin D., Buza’s G. M., Kral J., Şimşek H., Matysiak-Budnik T., Milivojevic V., Marlicz W., Venerito M., Boyanova L., Doulberis M., Capelle L. G., Cano-Català A., Moreira L., Nyssen O. P., Me’graud F., O’Morain C., Gisbert J. P. (2024). Role of compliance in *Helicobacter
pylori* eradication treatment: Results of the European Registry
on *H. pylori* management. United
Eur. Gastroenterol. J..

[123] Salvo F., De Sarro A., Caputi A. P., Polimeni G. (2009). Amoxicillin
and amoxicillin plus clavulanate: a safety review. Expert Opin. Drug Saf..

[124] Leung W. K., Graham D. Y. (2000). Clarithromycin for Helicobacter Pylori
infection. Expert Opin. Pharmacother..

[125] Roberts L. T., Issa P. P., Sinnathamby E. S., Granier M., Mayeux H., Eubanks T. N., Malone K., Ahmadzadeh S., Cornett E. M., Shekoohi S., Kaye A. D. (2022). Helicobacter
Pylori: A Review of Current Treatment Options in Clinical Practice. Life.

[126] Hafeez M., Qureshi Z. A., Khattak A. L., Saeed F., Asghar A., Azam K., Khan M. A. (2021). Helicobacter
Pylori
Eradication Therapy: Still a Challenge. Cureus.

[127] Shaalan H., Azrad M., Peretz A. (2024). The effect
of three
urease inhibitors on *H. pylori* viability, urease
activity and urease gene expression. Front.
Microbiol..

[128] Zhong Z., Wang X., Li J., Zhang B., Yan L., Xu S., Chen G., Gao H. (2022). A study on the diagnosis
of the *Helicobacter pylori* coccoid form with artificial
intelligence technology. Front. Microbiol..

[129] Reshetnyak V. I., Reshetnyak T. M. (2017). Significance
of dormant forms of *Helicobacter pylori* in ulcerogenesis. World J. Gastroenterol..

[130] Fauzia K. A., Effendi W. I., Alfaray R. I., Malaty H. M., Yamaoka Y., Mifthussurur M. (2024). Molecular Mechanisms of Biofilm Formation
in *Helicobacter pylori*. Antibiotics.

[131] Vijayan S. K., James E., Venugopal R. P. (2019). Appropriateness
of Helicobacter pylori Eradication Therapy in Gastroenterology Patients
of a Tertiary Care Hospital. J. Appl. Pharm.
Sci..

[132] Salcedo J. A., Al-Kawas F. (1998). Treatment of Helicobacter
pylori
infection. Arch. Intern. Med..

[133] Hassan S. T. S., Šudomova’ M. (2017). The Development of
Urease Inhibitors: What Opportunities Exist for Better Treatment of
Helicobacter pylori infection in Children?. Children.

[134] Francis A., Nair A. S., Gomez L. J., Rahim B. A., V S. R., R P.
G. (2020). Assessment of medication
adherence
in Helicobacter pylori positive patients on standard triple therapy:
a prospective study. Int. J. Basic Clin. Pharmacol..

[135] Barazorda-Ccahuana H.
L., Go’mez B., Mas F., Madurga S. (2020). Effect of pH on the Supramolecular Structure of *Helicobacter pylori* Urease by Molecular Dynamics Simulations. Polymers.

[136] Cunha E. S., Chen X., Sanz-Gaitero M., Mills D. J., Luecke H. (2021). Cryo-EM structure of *Helicobacter
pylori* urease with an inhibitor in the active site at 2.0
Å resolution. Nat. Commun..

[137] el Nujumi A. M., Dorrian C. A., Chittajallu R. S., Neithercut W. D., McColl K. E. (1991). Effect of inhibition of Helicobacter
pylori urease activity by acetohydroxamic acid on serum gastrin in
duodenal ulcer subjects. Gut.

[138] Ahmad S., Abdul Qadir M., Ahmed M., Imran M., Yousaf N., Asari A., Hameed A., Muddassar M. (2024). Acetylsalicylic
acid-sulfa drugs conjugates as potential urease inhibitors and anti-inflammatory
agents: bio-oriented drug synthesis, molecular docking, and dynamics
simulation studies. J. Biomol. Struct. Dyn..

[139] Ahmad S., Abdul Qadir M., Ahmed M., Imran M., Ahmad M., Yousaf N., Wani T. A., Zargar S., Ali I., Muddassar M. (2023). Exploring
the Potential of New Benzamide-Acetamide
Pharmacophore Containing Sulfonamide as Urease Inhibitors: Structure-Activity
Relationship, Kinetics Mechanism, and *In Silico* Studies. ACS Omega.

[140] Ahmad S., Abdul Qadir M., Ahmed M., Imran M., Yousaf N., Wani T. A., Zargar S., Ali I., Muddassar M. (2023). Exploring the potential of propanamide-sulfonamide
based drug conjugates as dual inhibitors of urease and cyclooxygenase-2:
biological and their *in silico* studies. Front. Chem..

[141] Ahmad S., Abdul Qadir M., Ahmed M., Imran M., Yousaf N., Wani T. A., Zargar S., Ali I., Muddassar M. (2023). New Acetamide-Sulfonamide-Containing
Scaffolds: Antiurease
Activity Screening, Structure-Activity Relationship, Kinetics Mechanism,
Molecular Docking, and MD Simulation Studies. Molecules.

[142] Mohammed S. O., El Ashry S. H. E., Khalid A., Amer M. R., Metwaly A. M., Eissa I. H., Elkaeed E. B., Elshobaky A., Hafez E. E. (2022). Expression, Purification, and Comparative
Inhibition
of *Helicobacter pylori* Urease by Regio-Selectively
Alkylated Benzimidazole 2-Thione Derivatives. Molecules.

[143] Talebi M., Hamidian E., Niasari-Naslaji F., Rahmani S., Hosseini F. S., Boumi S., Montazer M. N., Asadi M., Amanlou M. (2021). Synthesis, Molecular Docking, and
Biological Evaluation of Nitroimidazole Derivatives as Potent Urease
Inhibitors. Med. Chem. Res..

[144] Mao W. J., Lv P. C., Shi L., Li H. Q., Zhu H. L. (2009). Synthesis, molecular docking and
biological evaluation
of metronidazole derivatives as potent *Helicobacter pylori* urease inhibitors. Bioorg. Med. Chem..

[145] Huang X.-S., Liu K., Yin Y., Li W.-M., Ran W., Duan M., Wang L.-S., Zhu H.-L. (2011). The Synthesis, Structure
and Activity Evaluation of Secnidazole Derivatives as Helicobacter
pylori Urease Inhibitors. Curr. Bioact. Compd..

[146] Shahin A. I., Zaib S., Zaraei S.-O., Kedia R. A., Anbar H. S., Younas M. T., Al-Tel T. H., Khoder G., El-Gamal M. I. (2023). Design and synthesis of novel anti-urease
imidazothiazole
derivatives with promising antibacterial activity against *Helicobacter pylori*. PLoS One.

[147] Mamidala R., Bhimathati S. R. S., Vema A. (2021). Discovery of Novel
Dihydropyrimidine and hydroxamic acid hybrids as potent *Helicobacter
pylori* Urease Inhibitors. Bioorg. Chem..

[148] Liu Q., Shi W. K., Ren S. Z., Ni W. W., Li W. Y., Chen H. M., Liu P., Yuan J., He X. S., Liu J. J., Cao P., Yang P. Z., Xiao Z. P., Zhu H. L. (2018). Arylamino containing hydroxamic acids
as potent urease
inhibitors for the treatment of *Helicobacter pylori* infection. Eur. J. Med. Chem..

[149] Shi W. K., Deng R. C., Wang P. F., Yue Q. Q., Liu Q., Ding K. L., Yang M. H., Zhang H. Y., Gong S. H., Deng M., Liu W. R., Feng Q. J., Xiao Z. P., Zhu H. L. (2016). 3-Arylpropionylhydroxamic
acid derivatives as *Helicobacter pylori* urease inhibitors:
Synthesis, molecular
docking and biological evaluation. Bioorg. Med.
Chem..

[150] Ni W. W., Liu Q., Ren S. Z., Li W. Y., Yi L. L., Jing H., Sheng L. X., Wan Q., Zhong P. F., Fang H. L., Ouyang H., Xiao Z. P., Zhu H. L. (2018). The synthesis and
evaluation of phenoxyacylhydroxamic
acids as potential agents for *Helicobacter pylori* infections. Bioorg. Med. Chem..

[151] Xiao Z. P., Peng Z. Y., Dong J. J., Deng R. C., Wang X. D., Ouyang H., Yang P., He J., Wang Y. F., Zhu M., Peng X. C., Peng W. X., Zhu H. L. (2013). Synthesis, molecular docking and kinetic properties
of β-hydroxy-β-phenylpropionyl-hydroxamic acids as *Helicobacter pylori* urease inhibitors. Eur. J. Med. Chem..

[152] Azizian H., Nabati F., Sharifi A., Siavoshi F., Mahdavi M., Amanlou M. (2012). Large-scale virtual
screening for
the identification of new *Helicobacter pylori* urease
inhibitor scaffolds. J. Mol. Model..

[153] Biglar M., Mirzazadeh R., Asadi M., Sepehri S., Valizadeh Y., Sarrafi Y., Amanlou M., Larijani B., Mohammadi-Khanaposhtani M., Mahdavi M. (2020). Novel N,N-dimethylbarbituric-pyridinium
derivatives as potent urease inhibitors: synthesis, *in vitro*, and *in silico* studies. Bioorg.
Chem..

[154] Sedaghati S., Azizian H., Montazer M. N., Mohammadi-Khanaposhtani M., Asadi M., Moradkhani F., Ardestani M. S., Asgari M. S., Yahya-Meymandi A., Biglar M., Larijani B., Sadat-Ebrahimi S. E., Foroumadi A., Amanlou M., Mahdavi M. (2021). Novel (thio)­barbituric-phenoxy-N-phenylacetamide
derivatives as potent urease inhibitors: synthesis, *in vitro* urease inhibition, and *in silico* evaluations. Struct. Chem..

[155] Asgari M. S., Azizian H., Nazari Montazer M., Mohammadi-Khanaposhtani M., Asadi M., Sepehri S., Ranjbar P. R., Rahimi R., Biglar M., Larijani B., Amanlou M., Mahdavi M. (2020). New 1,2,3-triazole-(thio)­barbituric
acid hybrids as urease inhibitors: Design, synthesis, *in vitro* urease inhibition, docking study, and molecular dynamic simulation. Arch. Pharm..

[156] Li W. Y., Ni W. W., Ye Y. X., Fang H. L., Pan X. M., He J. L., Zhou T. L., Yi J., Liu S. S., Zhou M., Xiao Z. P., Zhu H. L. (2020). N-monoarylacetothioureas
as potent urease inhibitors: synthesis, SAR, and biological evaluation. J. Enzyme Inhib. Med. Chem..

[157] Ni W. W., Fang H. L., Ye Y. X., Li W. Y., Liu L., Fu Z. J., Dawalamu, Zhu W. Y., Li K., Li F., Zou X., Ouyang H., Xiao Z. P., Zhu H. L. (2021). Synthesis and Structure-Activity
Relationship Studies of N-Monosubstituted Aroylthioureas as Urease
Inhibitors. Med. Chem..

[158] Zahra U., Zaib S., Saeed A., Rehman M. U., Shabir G., Alsaab H. O., Khan I. (2022). New acetylphenol-based
acyl thioureas broaden the scope of drug candidates for urease inhibition:
synthesis, *in vitro* screening and *in silico* analysis. Int. J. Biol. Macromol..

[159] Yaqoob S., Hameed A., Ahmed M., Imran M., Qadir M. A., Ramzan M., Yousaf N., Iqbal J., Muddassar M. (2022). Antiurease screening of alkyl chain-linked
thiourea
derivatives: *in vitro* biological activities, molecular
docking, and dynamic simulations studies. RSC
Adv..

[160] Xiao Z. P., Peng Z. Y., Dong J. J., He J., Ouyang H., Feng Y. T., Lu C. L., Lin W. Q., Wang J. X., Xiang Y. P., Zhu H. L. (2013). Synthesis, structure-activity
relationship analysis and kinetics study of reductive derivatives
of flavonoids as *Helicobacter pylori* urease inhibitors. Eur. J. Med. Chem..

[161] Xiao Z. P., Wang X. D., Peng Z. Y., Huang S., Yang P., Li Q. S., Zhou L. H., Hu X. J., Wu L. J., Zhou Y., Zhu H. L. (2012). Molecular
Docking,
Kinetics Study, and Structure-Activity Relationship Analysis of Quercetin
and Its Analogous as *Helicobacter pylori* Urease Inhibitors. J. Agric. Food Chem..

[162] Yu X. D., Zheng R. B., Xie J. H., Su J. Y., Huang X. Q., Wang Y. H., Zheng Y. F., Mo Z. Z., Wu X. L., Wu D. W., Liang Y., Zeng H.-F., Su Z.-R., Huang P. (2015). Biological evaluation and molecular
docking of baicalin and scutellarin as *Helicobacter pylori* urease inhibitors. J. Ethnopharmacol..

[163] Sharaf M., Arif M., Hamouda H. I., Khan S., Abdalla M., Shabana S., Rozan H. E., Khan T. U., Chi Z., Liu C. (2022). Preparation, urease
inhibition mechanisms, and anti-*Helicobacter pylori* activities of hesperetin-7-rhamnoglucoside. Curr. Res. Microb. Sci..

[164] Kataria R., Khatkar A. (2019). *In-Silico* Designing,
ADMET Analysis, Synthesis and Biological Evaluation of Novel Derivatives
of Diosmin Against Urease Protein and *Helicobacter pylori* Bacterium. Curr. Top. Med. Chem..

[165] Kataria R., Khatkar A. (2019). Molecular docking,
synthesis, kinetics
study, structure-activity relationship and ADMET analysis of morin
analogous as *Helicobacter pylori* urease inhibitors. BMC Chem..

[166] Xiao Z. P., Ma T. W., Fu W. C., Peng X. C., Zhang A. H., Zhu H. L. (2010). The synthesis, structure
and activity
evaluation of pyrogallol and catechol derivatives as *Helicobacter
pylori* urease inhibitors. Eur. J. Med.
Chem..

[167] Chopdar K. S., Dash G. C., Mohapatra P. K., Nayak B., Raval M. K. (2019). In-silico
Design of Covalently Bonding
Catechol based Urease Inhibitors as Potential Candidates for Treatment
of *Helicobacter pylori* Infection. Int. J. Pharma Res. Health Sci..

[168] Borges F., Roleira F., Milhazes N., Santana L., Uriarte E. (2005). Simple coumarins and analogues in
medicinal chemistry:
occurrence, synthesis and biological activity. Curr. Med. Chem..

[169] Jadhav S. G., Meshram R. J., Gond D. S., Gacche R. N. (2013). Inhibition
of growth of *Helicobacter pylori* and its urease by
coumarin derivatives: Molecular docking analysis. J. Pharm. Res..

[170] Kataria R., Khatkar A. (2019). *In-silico* design,
synthesis, ADMET studies and biological evaluation of novel derivatives
of chlorogenic acid against urease protein and *H. pylori* bacterium. BMC Chem..

[171] Verma A., Joshi S., Singh D. (2013). Imidazole: Having Versatile
Biological Activities. J. Chem..

[172] Muri E. M. F., Nieto M. J., Sindelar R. D., Williamson J. S. (2002). Hydroxamic
Acids as Pharmacological Agents. Curr. Med.
Chem..

[173] Kaur N., Kaur M., Sohal H. S., Han H., Bhowmik P. K. (2024). A Review
on Barbituric Acid and Its Derivatives: Synthesis,
Reactions, and Bio-Applications. Organics.

[174] Naz S., Zahoor M., Umar M. N., Alghamdi S., Sahibzada M. U. K., UlBari W. (2020). Synthesis, characterization,
and pharmacological evaluation
of thiourea derivatives. Open Chem..

[175] Kumar S., Pandey A. K. (2013). Chemistry and Biological
Activities
of Flavonoids: An Overview. Sci. World J..

[176] Quercetin. LiverTox: Clinical and Research Information on Drug-Induced Liver Injury; National Institute of Diabetes and Digestive and Kidney Diseases, 2012.

[177] Andres S., Pevny S., Ziegenhagen R., Bakhiya N., Schäfer B., Hirsch-Ernst K. I., Lampen A. (2018). Safety Aspects of the Use of Quercetin as a Dietary
Supplement. Mol. Nutr. Food Res..

[178] Yang J., Li M., Zhang C., Liu D. (2021). Pharmacological
properties of baicalin on liver diseases: a narrative review. Pharmacol. Rep..

[179] Li X., Wang L., Li Y., Bai L., Xue M. (2011). Acute and
subacute toxicological evaluation of scutellarin in rodents. Regul. Toxicol. Pharmacol..

[180] Kocaçalışkan I., Talan I., Terzi I. (2006). Antimicrobial
Activity of Catechol and Pyrogallol as Allelochemicals. Z. Naturforsch., C: J. Biosci..

[181] Lake B. G. (1999). Coumarin Metabolism, Toxicity and Carcinogenicity:
Relevance for Human Risk Assessment. Food Chem.
Toxicol..

[182] Joneidi S., Alizadeh S. R., Ebrahimzadeh M. A. (2024). Chlorogenic
Acid Derivatives: Structural Modifications, Drug Design, and Biological
Activities: A Review. Mini Rev. Med. Chem..

[183] Knegtel R. M., Kuntz I. D., Oshiro C. M. (1997). Molecular
docking
to ensembles of protein structures. J. Mol.
Biol..

[184] Chen Y., Shi Y., Ming D., Huang H., Jiang L. (2025). Reversible peptide tagging system
to regulate enzyme activity. Cell Rep. Phys.
Sci..

