# Supplementary material for: Targeting Urease: A Promising Adjuvant Strategy for Effective Helicobacter pylori Eradication
Source: ACS Omega. 2025 Jul 4;10(27):28643–69. doi: 10.1021/acsomega.5c02725 (PMC12268749; doi:10.1021/acsomega.5c02725)
Supplement: Supplementary file 1 [file ao5c02725_si_001.pdf]

Targeting Urease: A Promising Adjuvant Strategy for Effective *H. pylori* Eradication

Shivani Kunkalienkar[1], Neha S. Gandhi[2], Ashutosh Gupta[1], Moumita Saha[1], Aravinda Pai[3], Shiran Shetty[4], Abhishek Gupta[5], Namdev Dhas[6], Raghu C Hariharapura[7], Krishnadas Nandakumar[8], Nagalakshmi Narasimhaswamy[9], Sudheer Moorkoth[1]*

[1]Department of Pharmaceutical Quality Assurance, Manipal College of Pharmaceutical Sciences, Manipal Academy of Higher Education, Karnataka, Manipal, 576 104, India.

[2]Department of Biotechnology, Manipal Institute of Technology, Manipal Academy of Higher Education, Manipal, 576 104, India.

[3]Department of Pharmaceutical Chemistry, Manipal College of Pharmaceutical Sciences, Manipal Academy of Higher Education, Karnataka, Manipal, 576 104, India.

[4]Department of Gastroenterology and Hepatology, Kasturba Medical College, Manipal Academy of Higher Education, Karnataka, Manipal, 576 104, India.

[5]School of Pharmacy and Life Sciences, Faculty of Science and Engineering, Wulfruna Street, University of Wolverhampton, WV1 1LY, United Kingdom

[6]Department of Pharmaceutics, Manipal College of Pharmaceutical Sciences, Manipal Academy of Higher Education, Karnataka, Manipal, 576 104, India.

[7]Department of Pharmaceutical Biotechnology, Manipal College of Pharmaceutical Sciences, Manipal Academy of Higher Education, Karnataka, Manipal, 576 104, India.

[8]Department of Pharmacology, Manipal College of Pharmaceutical Sciences, Manipal Academy of Higher Education, Karnataka, Manipal, 576 104, India.

[9]Department of Microbiology, Melaka Manipal Medical College, Manipal Academy of Higher Education, Karnataka, Manipal, 576 104, India.

Corresponding Author

*Sudheer Moorkoth

Contact no.- 9980371383

Email address: moorkoth.s@manipal.edu

Supporting information. Docking score and interactions of potent compounds (Table S1); Interactions of compound 4, 10, 13, 17, 19, 26, 32, and 33 with amino acid residues at urease active site (Figure S1-S24)

Table S1. Docking score and interactions of potent compounds

Compounds	Docking score			Interactions in active site residues		
	**1E9Y**	**6ZJA**	**6QSU**	**1E9Y**	**6ZJA**	**6QSU**
Compound 4	-4.489	-4.273	-5.710	**Hydrogen bond interactions:** oxygen of $COOCH_3$ on benzene ring with HIE221, hydroxyl moiety with ASN168<br><br>**Metal coordination:** oxygen of $COOCH_3$ on benzene ring with nickel ion<br><br>**Hydrophobic interactions:** MET366, ALA365, MET317, ILE140, CYS321, ALA169	**Hydrogen bond interactions:** oxygen of $COOCH_3$ on benzene ring with HIE221,<br><br>**Metal coordination:** oxygen of $COOCH_3$ on benzene ring with nickel ion<br><br>**Hydrophobic interactions:** ALA365, MET366, ALA169, ILE467, MET317, LEU318, CYS321, ALA278	**Hydrophobic interactions:** MET366, ALA365, PRO468, ILE467, ALA169, PHE45, CYS321

Compound 10	-7.228	-7.521	-7.564	**Hydrogen bond interactions:** oxygen of hydroxamic acid moiety with ALA365, ASP362, HIE221<br><br>**Metal coordination:** oxygen of hydroxamic acid moiety with nickel ions<br><br>**Hydrophobic interactions:** ALA365, MET366, MET317, VAL320, CYS321, ALA169	**Hydrogen bond interactions:** oxygen of hydroxamic moiety with ASP362, ALA365, HIE221, carbon of hydroxamic acid moiety with ALA365<br><br>**Metal coordination:** oxygen of hydroxamic acid moiety with nickel ions<br><br>**Hydrophobic interactions:** ILE339, MET317, LEU318, ALA278, CYS321, ALA169, ILE467, ALA365, MET366	**Hydrogen bond interactions:** hydroxyl moiety of hydroxamic acid with ALA169, NH with ALA365, hydroxyl moiety on benzene ring with GLY47<br><br>**Metal coordination:** oxygen of hydroxamic acid moiety with nickel ion<br><br>**Hydrophobic interactions:** ALA365, ALA169, MET366, ILE467, CYS321
Compound 13	-4.113	-5.150	-4.021	**Hydrogen bond interactions:** barbituric acid moiety with ARG338, GLY279<br><br>**Metal coordination:** barbituric acid	**Hydrogen bond interactions:** barbituric acid moiety with HIE221, ALA169<br><br>**Metal coordination:** barbituric acid	**Hydrogen bond interactions:** barbituric acid moiety with ALA365, NH attached to benzene with HIS322, oxygen with GLY47

				moiety with nickel ion<br><br>**Hydrophobic interactions:** ALA365, MET366, MET317, VAL320, CYS321, ALA169	moiety with nickel ions<br><br>**Hydrophobic interactions:** ALA122, PRO468, ILE467, CYS321, MET317, MET366, ALA365, ALA169	**Metal coordination:** barbituric acid moiety with nickel ion<br><br>**π-π stacking:** benzene ring with HIS322<br><br>**Hydrophobic interactions:** ALA169, PHE45, ALA365, MET366, CYS321, LEU324, LEU252, PHE334
Compound 17	-5.085	-4.059	-3.418	**Hydrogen bond interactions:** oxygen on benzene ring with HIE221<br><br>**Metal coordination:** oxygen on benzene ring with nickel ions<br><br>**π-cation stacking:** benzene ring with ARG338<br><br>**Hydrophobic interactions:** ALA365, MET366,	**Hydrogen bond interactions:** hydroxyl on benzene ring with CYS321, NH of thiourea with ASP223, S of thiourea with water<br><br>**Metal coordination:** oxygen and N with nickel ion, bromine with ASP362<br><br>**π-π stacking:** benzene ring with HIS322	**Hydrogen bond interactions:** oxygen of acetyl moiety with HIE221<br><br>**Metal coordination:** oxygen of acetyl moiety with nickel ion<br><br>**π-π stacking:** benzene ring with HIS322<br><br>**Hydrophobic interactions:** ALA365, MET366,

				CYS321, ALA169	**Hydrophobic interactions:** CYS321, LEU318, MET366, ALA365, ALA278, PHE45, ALA169	PRO468, ILE467, ALA169, PHE45, CYS321
Compound 19	-5.321	-7.069	-4.986	**Hydrogen bond interactions:** hydroxyl moiety with ASP362, ALA169 **Metal coordination:** hydroxyl moiety with nickel ions **π-cation stacking:** benzene ring with ARG338 **π-π stacking:** benzene ring with HIS322 **Hydrophobic interactions:** ALA365, MET366, MET317, CYS321, ALA169	**Hydrogen bond interactions:** hydroxyl moiety with ASP362, ALA169, CYS321, oxygen with HIE221 **Metal coordination:** hydroxyl moiety with nickel ions **Hydrophobic interactions:** CYS321, MET366, ALA365, ALA169	**Hydrogen bond interactions:** hydroxyl moiety with ALA365 **π-cation stacking:** benzene ring with ARG338 **Hydrophobic interactions:** ALA365, CYS321, MET366, ALA169
Compound 26	-6.578	-8.042	-7.129	**Hydrogen bond interactions:** hydroxyl	**Hydrogen bond interactions:** hydroxyl moiety	**Hydrogen bond interactions:**

				moiety with ASP362, ALA169<br>**Metal coordination:** hydroxyl moiety with nickel ions<br>**π-cation stacking:** benzene ring with ARG338<br>**Hydrophobic interactions:** ALA365, MET366, MET317, CYS321, ALA169	on benzene ring with ASP362, ALA169, HIE314<br>**Metal coordination:** hydroxyl moiety on benzene ring with nickel ion<br>**Hydrophobic interactions:** ALA365, CYS321, ALA169	hydroxyl moiety with ALA169, ASP362, GLN364<br>**Metal coordination:** hydroxyl moiety with nickel ion<br>**π-π stacking:** benzene ring with HIE248<br>**Hydrophobic interactions:** ALA365, MET366, PRO468, ILE467, CYS321, ALA169
Compound 32	-5.105	-6.554	-5.284	**Hydrogen bond interactions:** oxygen on benzene ring with HIE221<br>**Metal coordination:** oxygen on benzene ring with nickel ions<br>**π-π stacking:** benzene ring with HIE221	**Hydrogen bond interactions:** oxygen with water<br>**Metal coordination:** oxygen on benzene ring with nickel ions<br>**Hydrophobic interactions:** ALA365, CYS321, ALA169, MET366	**Hydrogen bond interactions:** hydroxyl on benzene ring with ALA365, ARG338<br>**Hydrophobic interactions:** MET366, ALA365, ALA169, CYS321, PHE45

				**Hydrophobic interactions:** ALA365, CYS321, ALA169		
Compound 33	-6.926	-8.145	-8.767	**Hydrogen bond interactions:** hydroxyl moiety with ALA169<br><br>Hydrophobic interactions: ALA365, CYS321, MET366, MET317, ALA365	**Hydrogen bond interactions:** hydroxyl moiety with ALA169, oxygen with water molecule<br><br>Hydrophobic interactions: ALA278, MET366, ALA365, MET317, VAL320, CYS321, ILE467	**Hydrogen bond interactions:** hydroxyl moiety with ALA169, ASP362, CYS321, oxygen of nitro group with GLY47<br><br>Metal coordination: hydroxyl moiety with nickel ion<br><br>Hydrophobic interactions: ALA365, MET366, ALA169, PHE334, LEU252, LEU324, CYS321, PHE45

1. Interaction of potent compounds on PDB ID 1E9Y

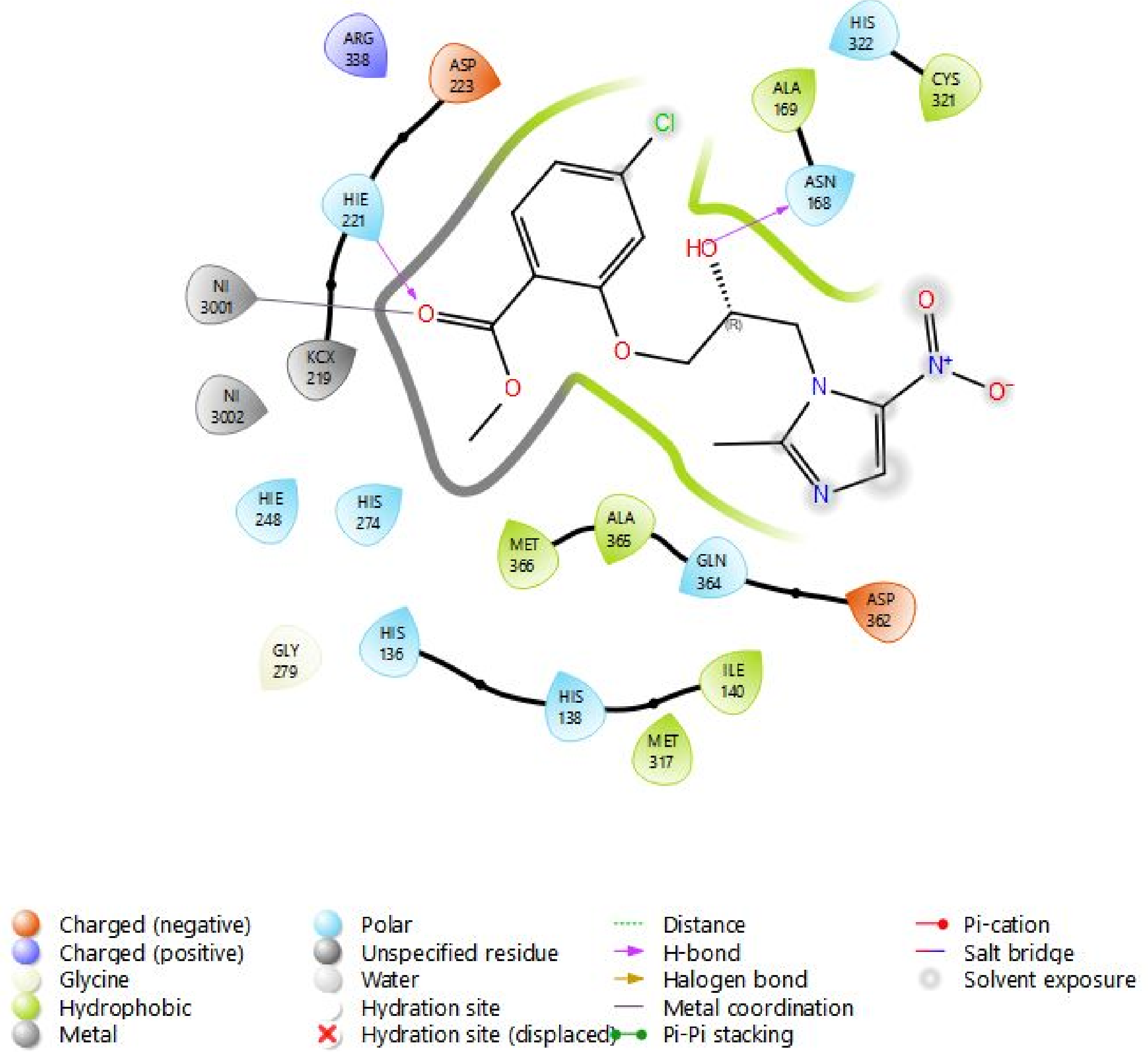

Figure S1. Interactions of compound 4 with amino acid residues at urease active site (PDB ID 1E9Y)

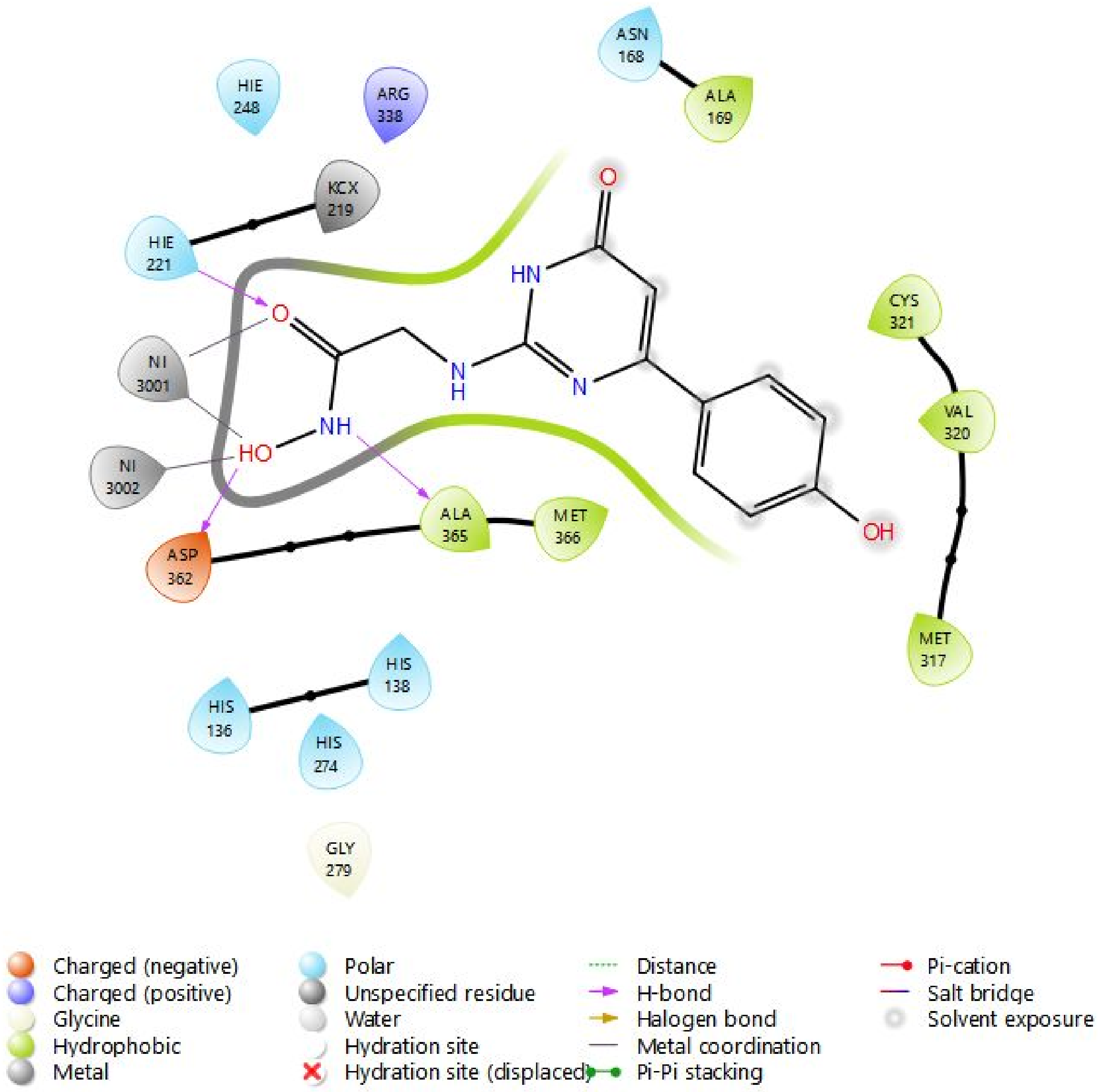

Figure S2. Interactions of compound 10 with amino acid residues at urease active site (PDB ID 1E9Y)

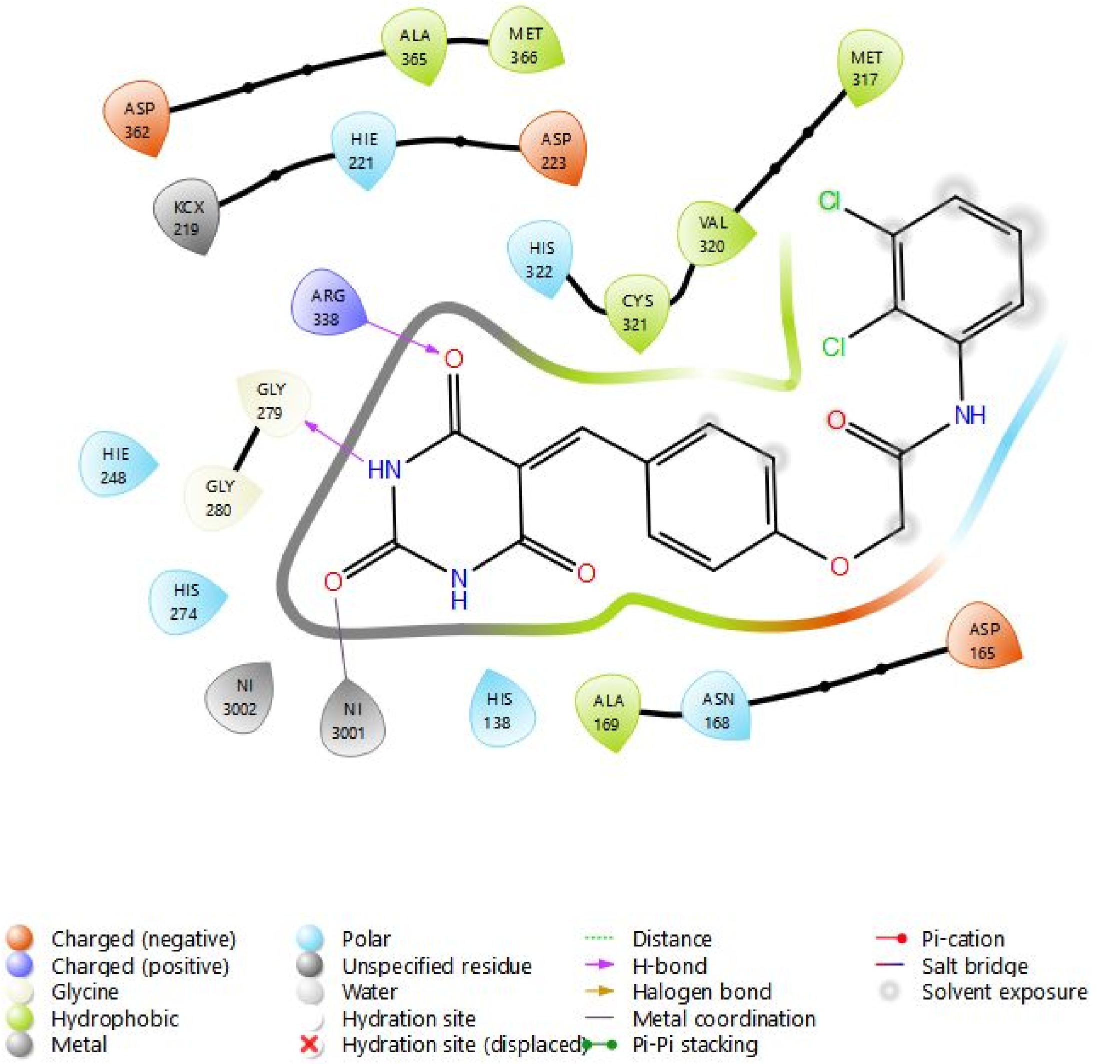

Figure S3. Interactions of compound 13 with amino acid residues at urease active site (PDB ID 1E9Y)

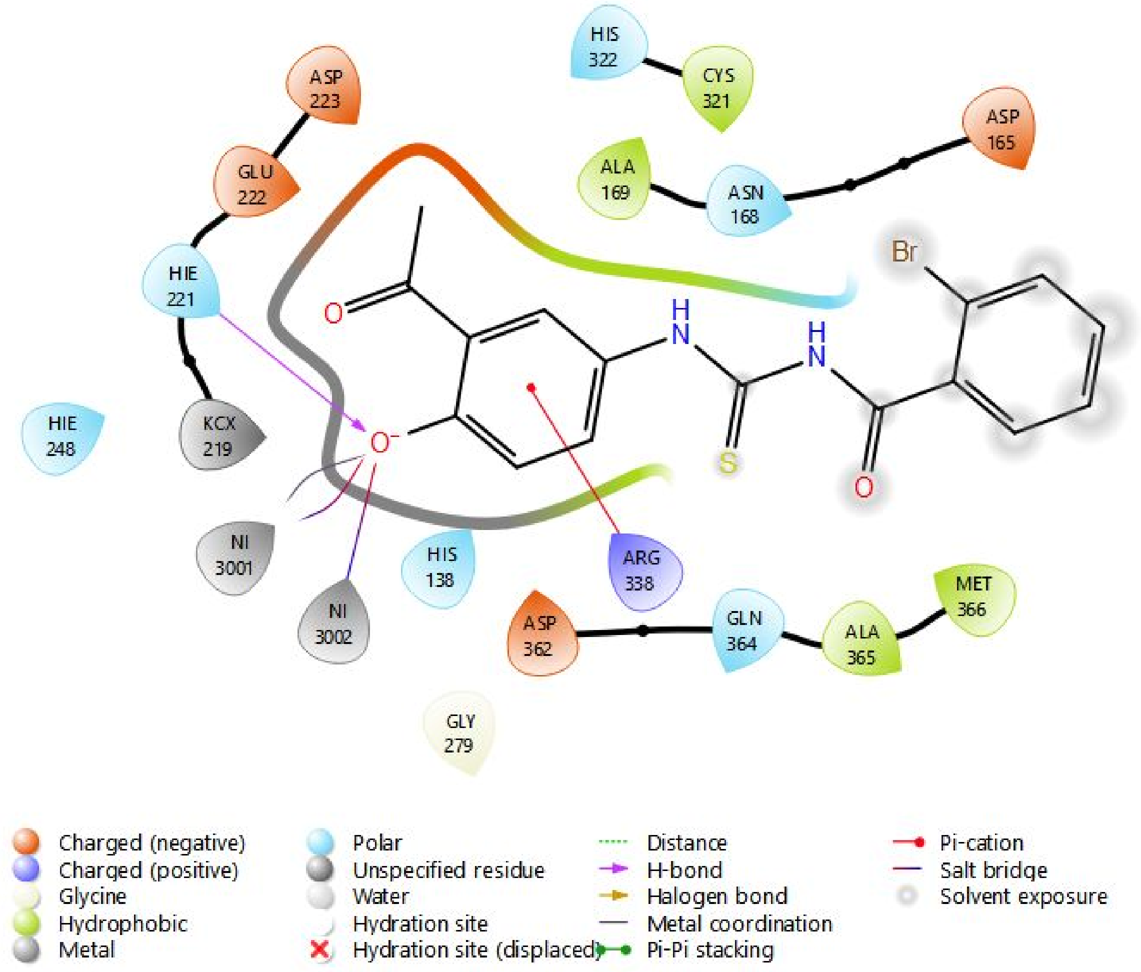

Figure S4. Interactions of compound 17 with amino acid residues at urease active site (PDB ID 1E9Y)

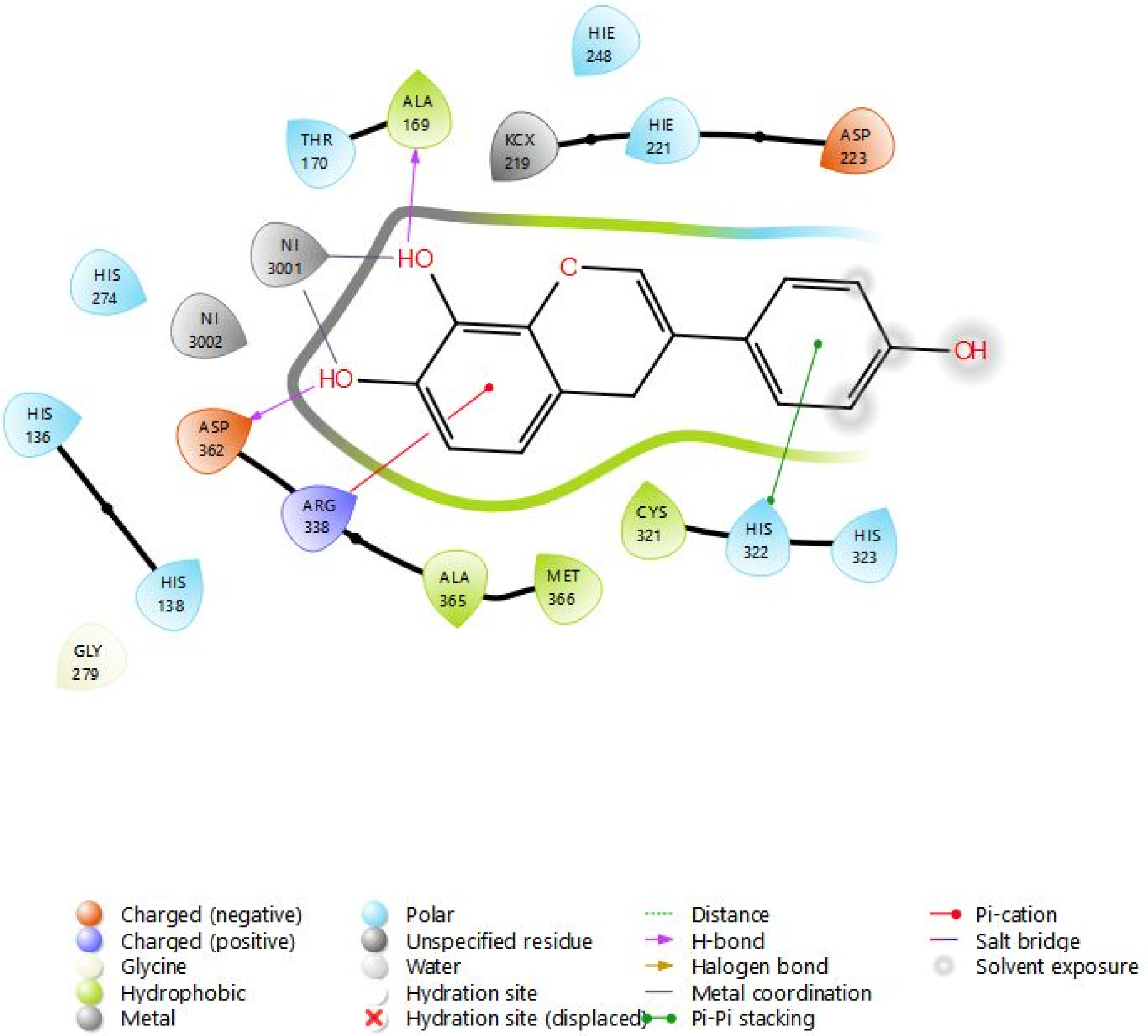

Figure S5. Interactions of compound 19 with amino acid residues at urease active site (PDB ID 1E9Y)

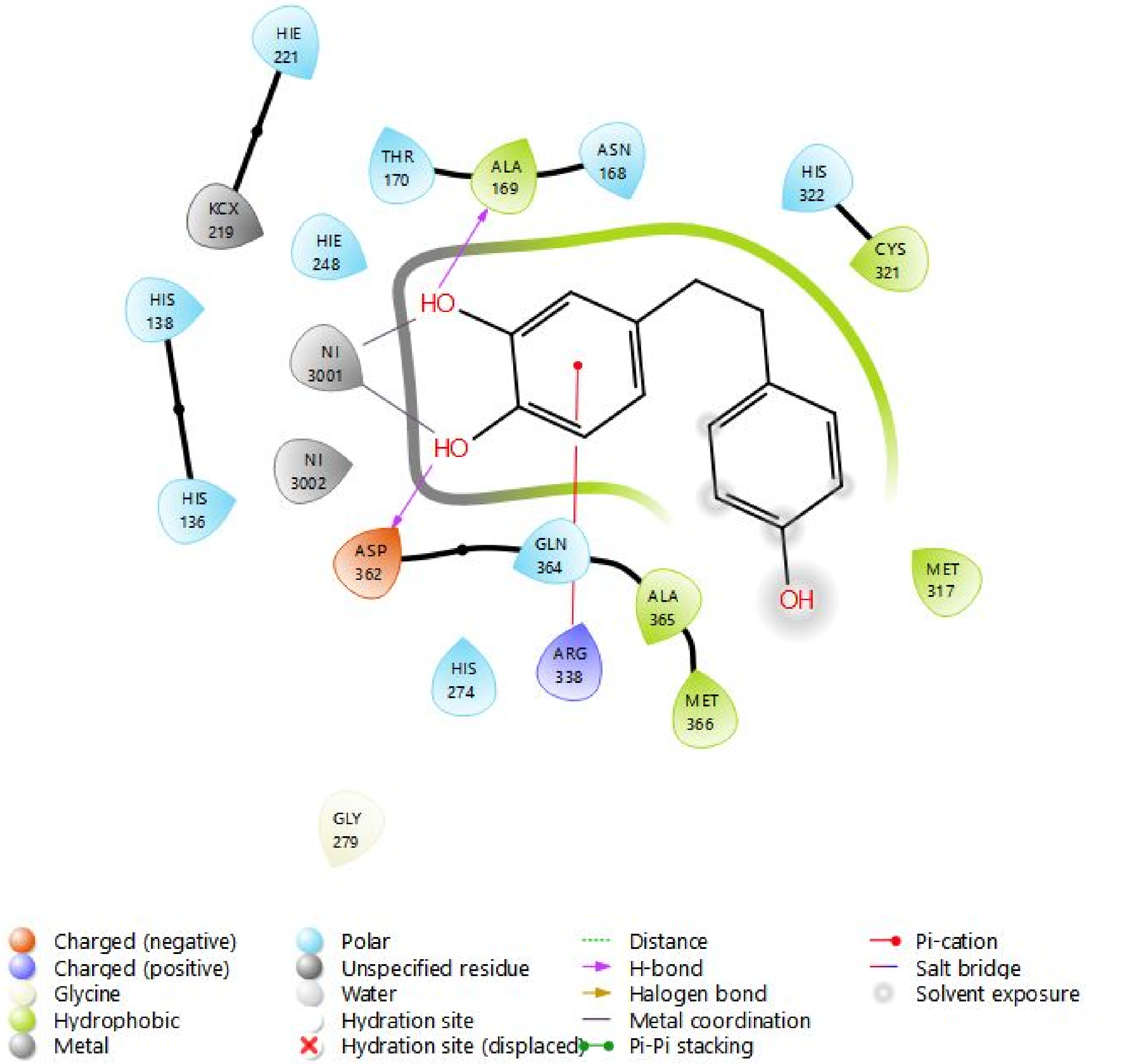

Figure S6. Interactions of compound 26 with amino acid residues at urease active site (PDB ID 1E9Y)

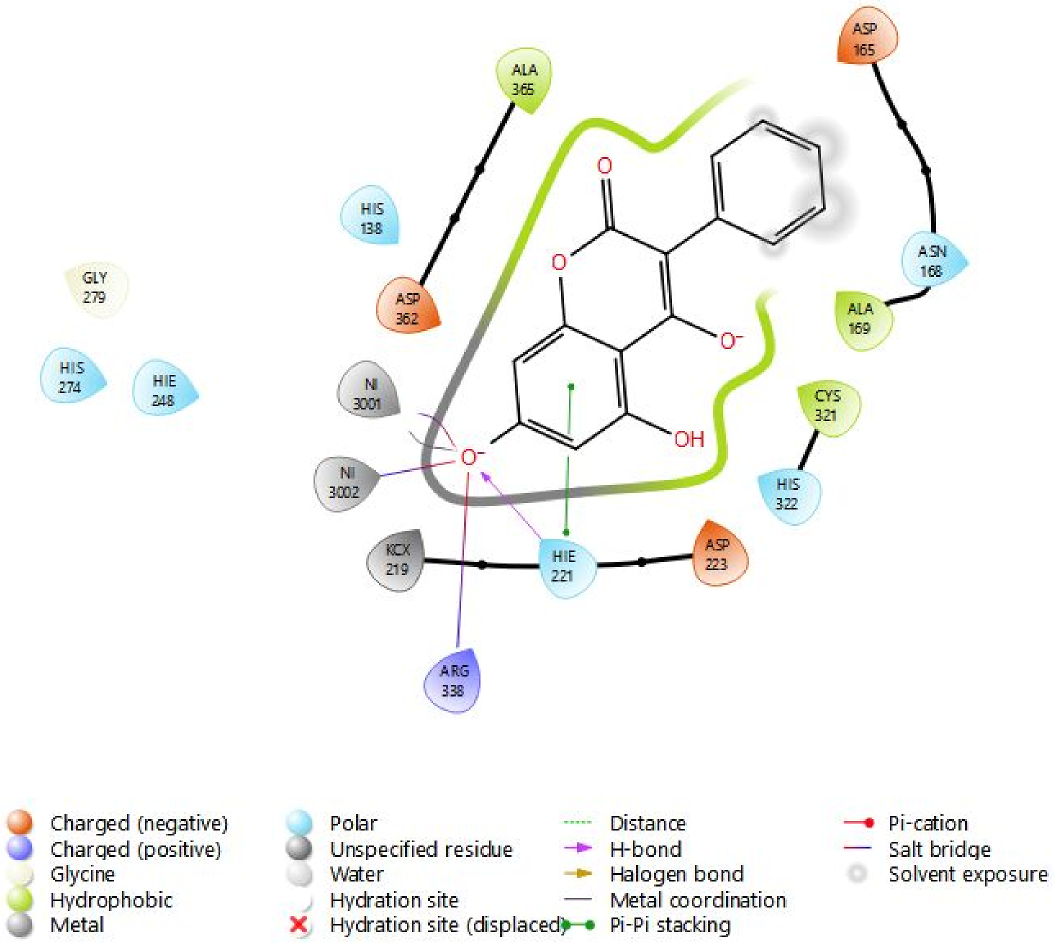

Figure S7. Interactions of compound 32 with amino acid residues at urease active site (PDB ID 1E9Y)

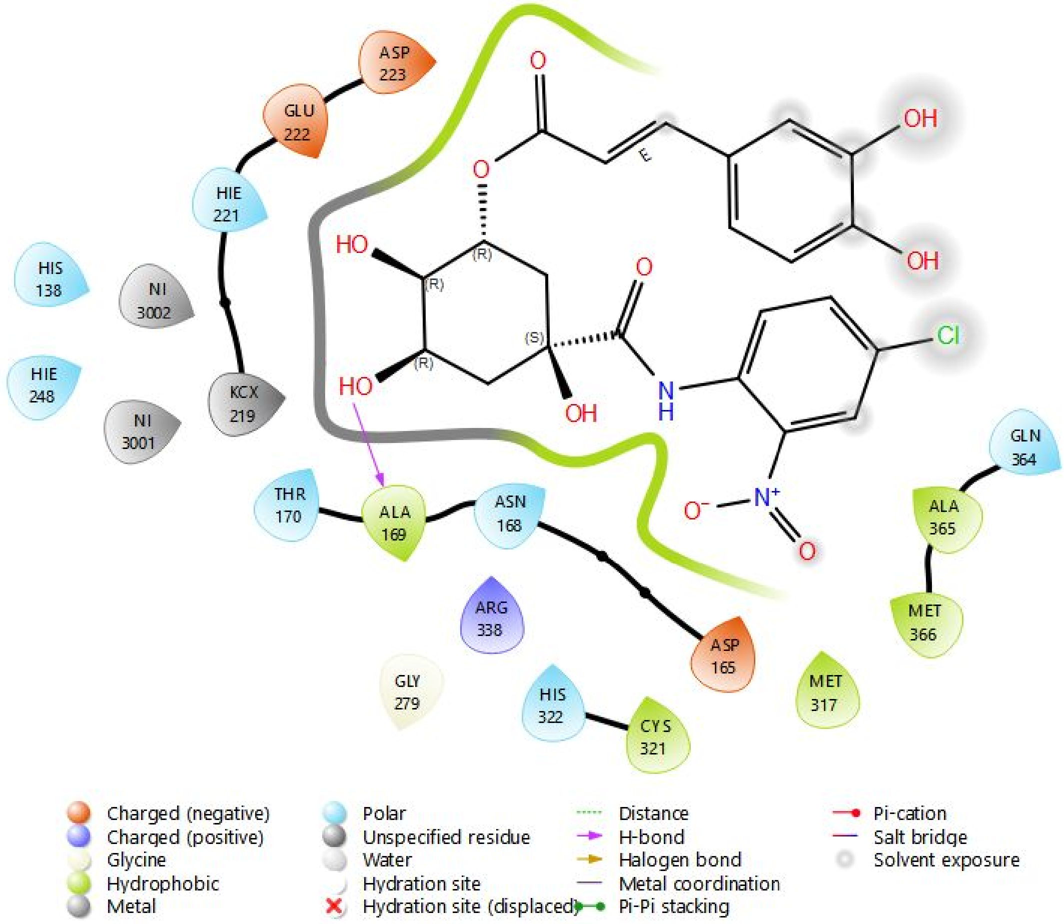

Figure S8. Interactions of compound 33 with amino acid residues at urease active site (PDB ID 1E9Y)

2. Interaction of potent compounds on PDB ID 6ZJA

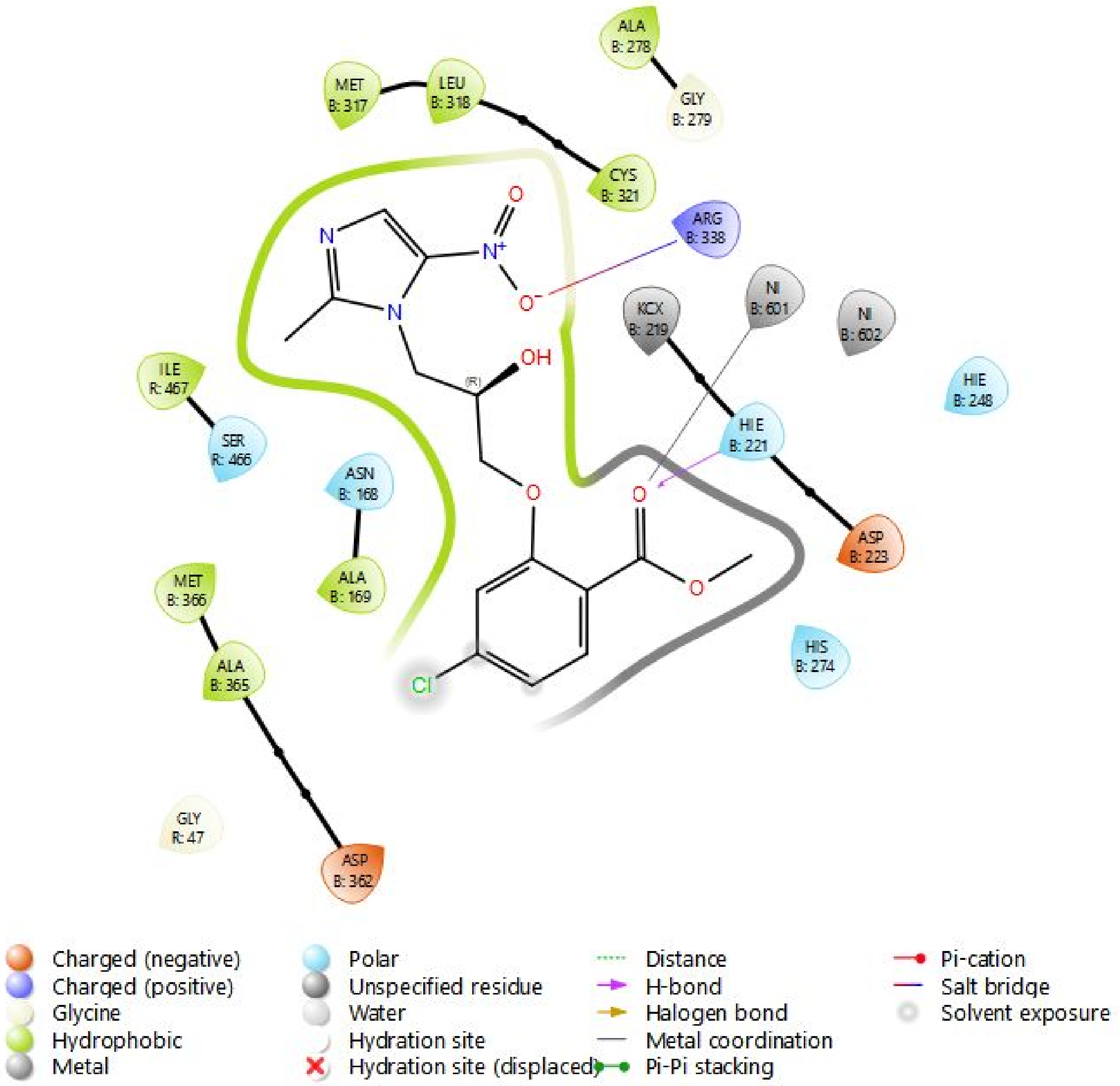

Figure S9. Interactions of compound 4 with amino acid residues at urease active site (PDB ID 6ZJA)

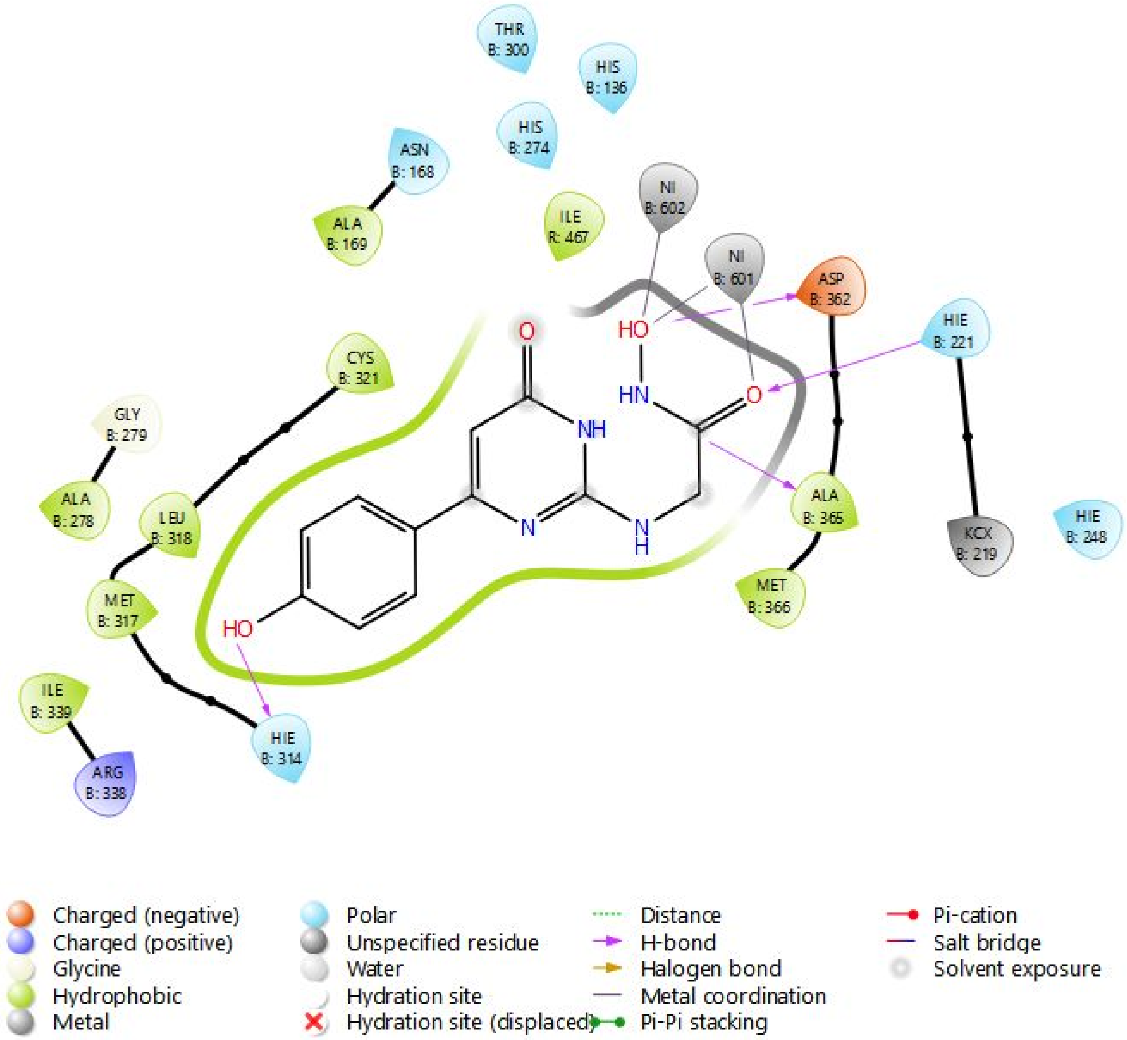

Figure S10. Interactions of compound 10 with amino acid residues at urease active site (PDB ID 6ZJA)

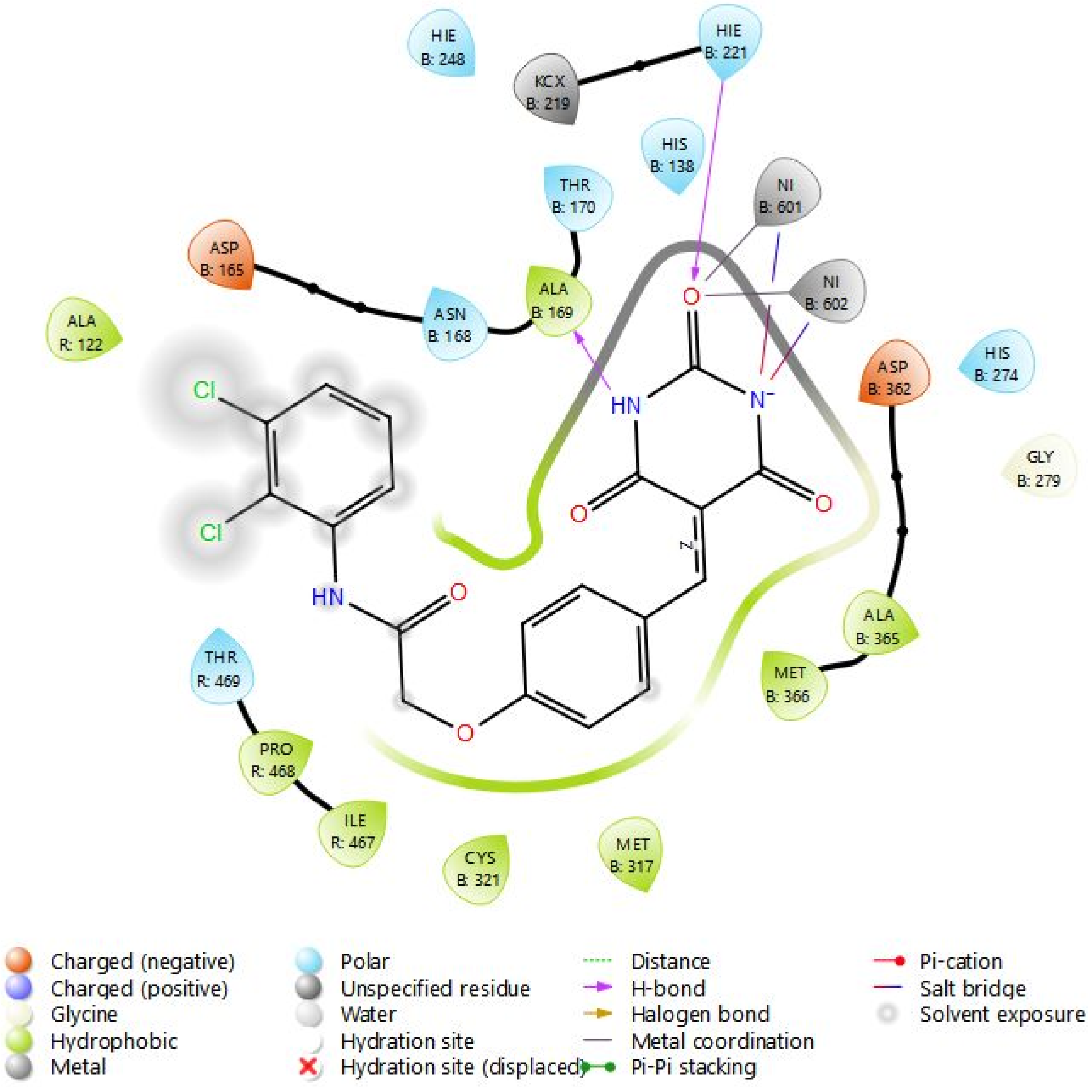

Figure S11. Interactions of compound 13 with amino acid residues at urease active site (PDB ID 6ZJA)

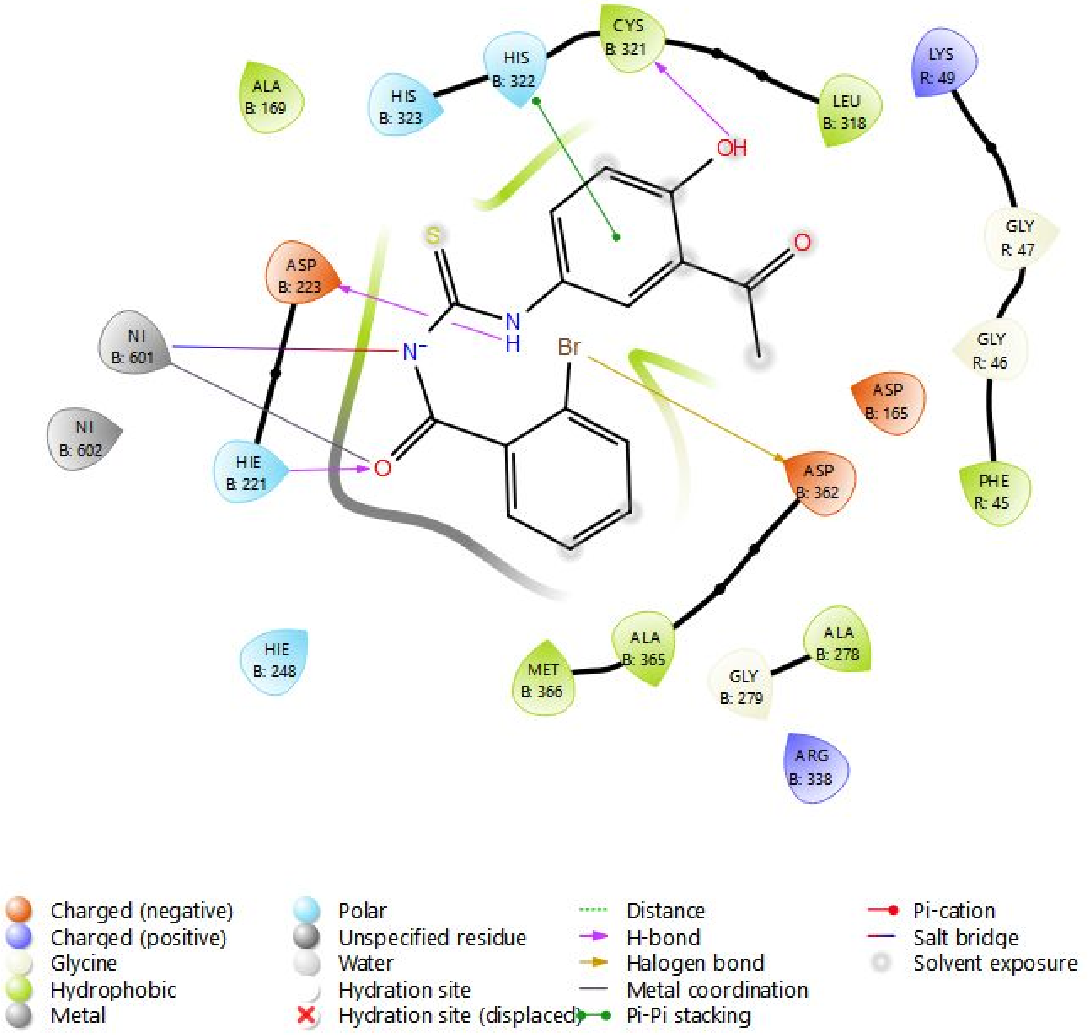

Figure S12. Interactions of compound 17 with amino acid residues at urease active site (PDB ID 6ZJA)

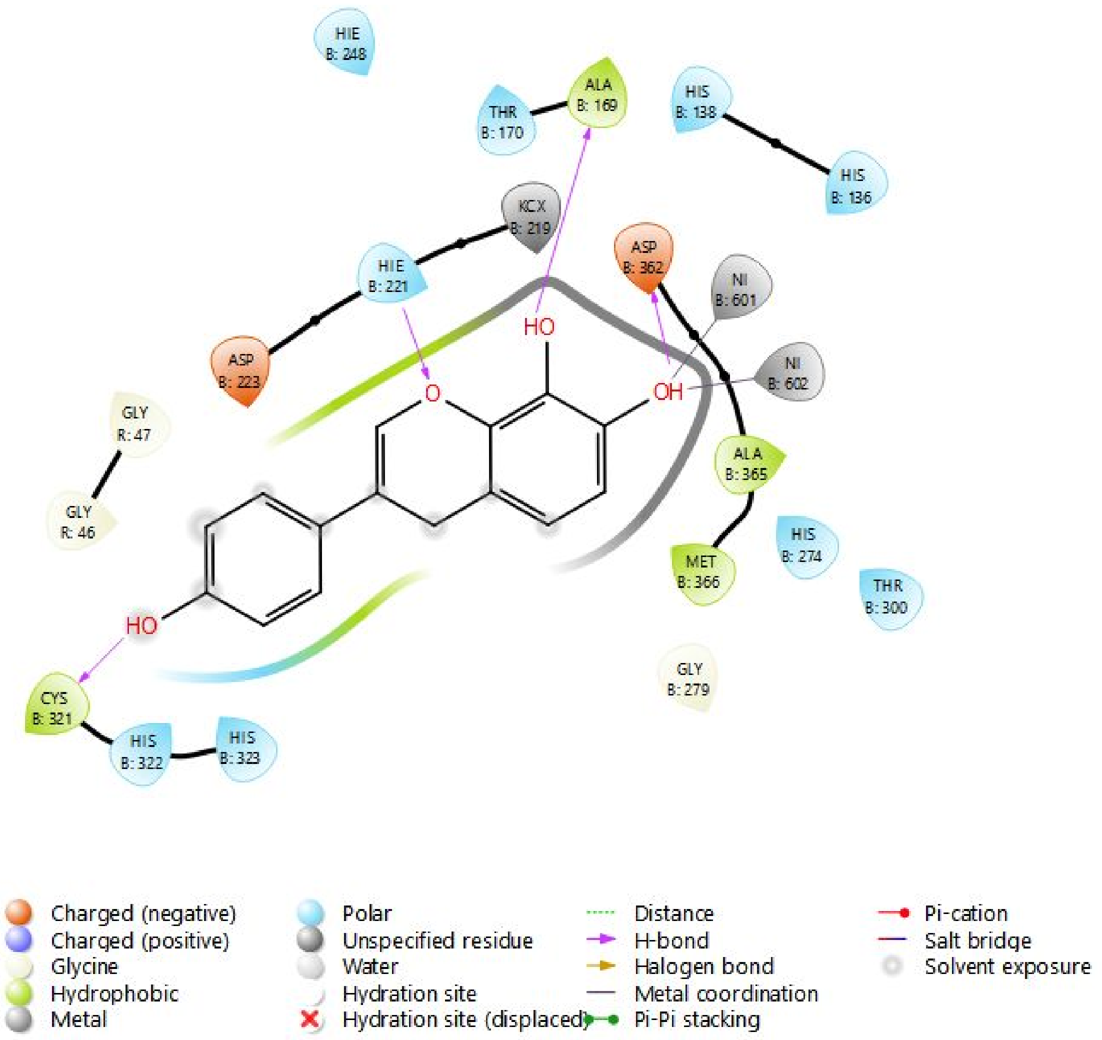

Figure S13. Interactions of compound 19 with amino acid residues at urease active site (PDB ID 6ZJA)

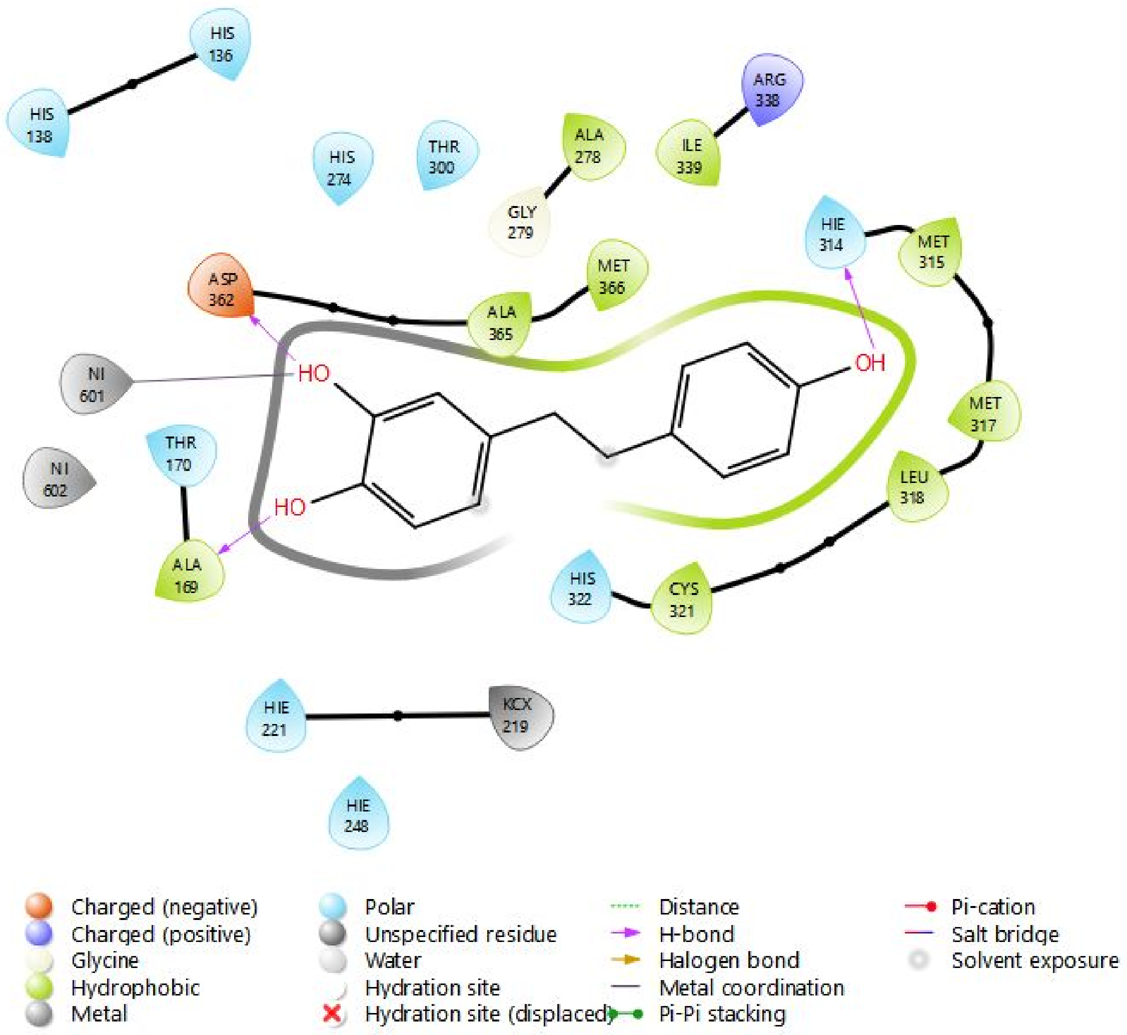

Figure S14. Interactions of compound 26 with amino acid residues at urease active site (PDB ID 6ZJA)

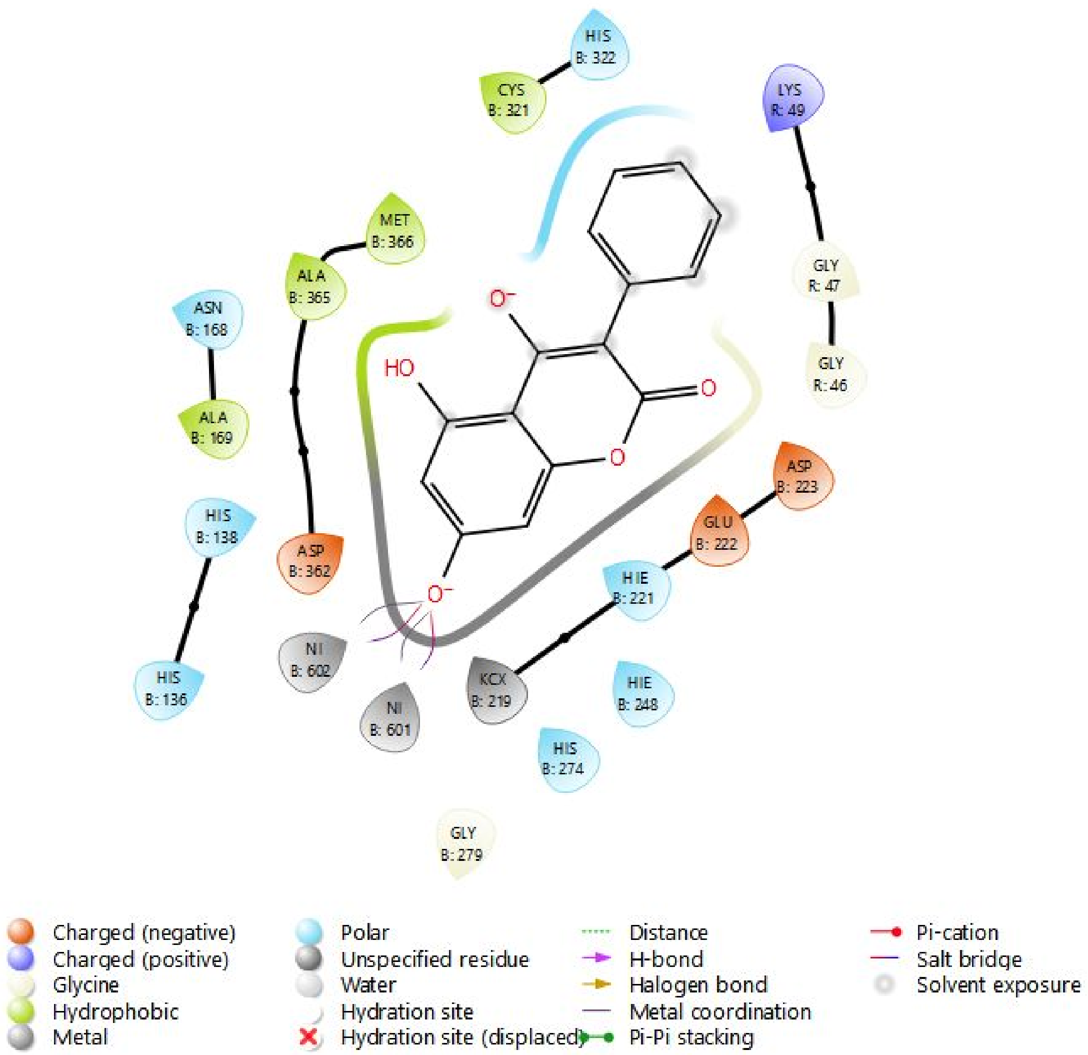

Figure S15. Interactions of compound 32 with amino acid residues at urease active site (PDB ID 6ZJA)

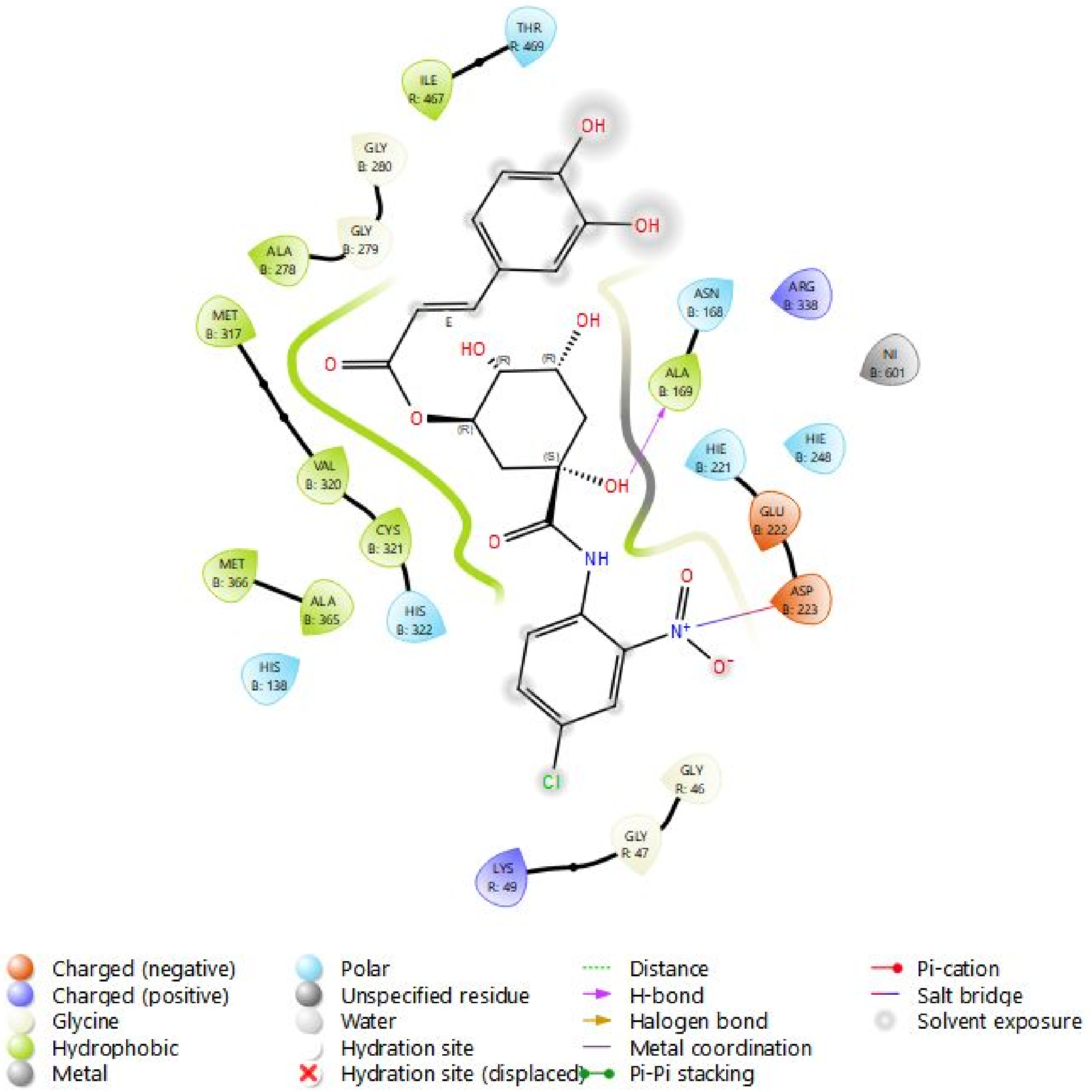

Figure S16. Interactions of compound 33 with amino acid residues at urease active site (PDB ID 6ZJA)

3. Interaction of potent compounds on PDB ID 6QSU

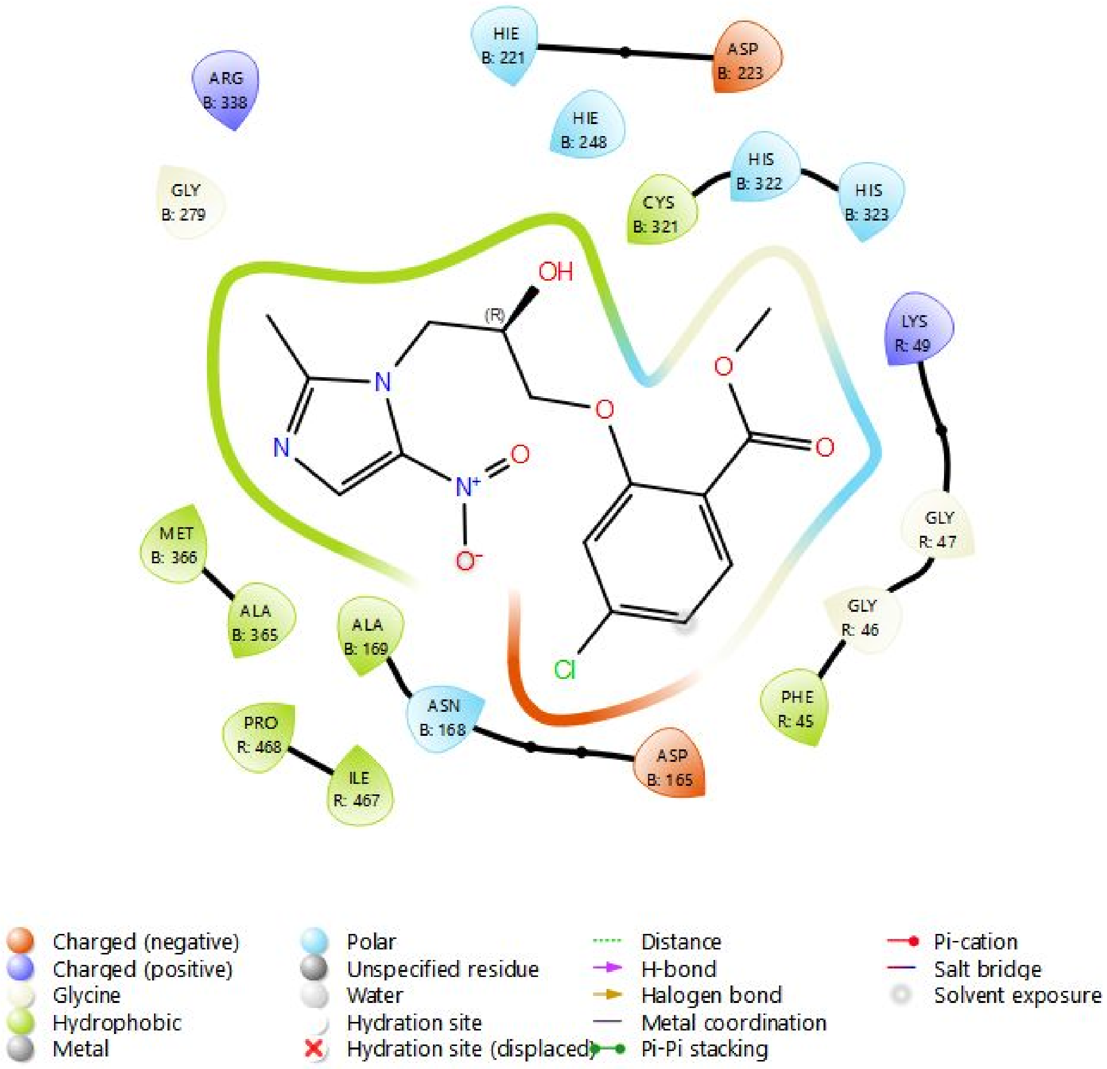

Figure S17. Interactions of compound 4 with amino acid residues at urease active site (PDB ID 6QSU)

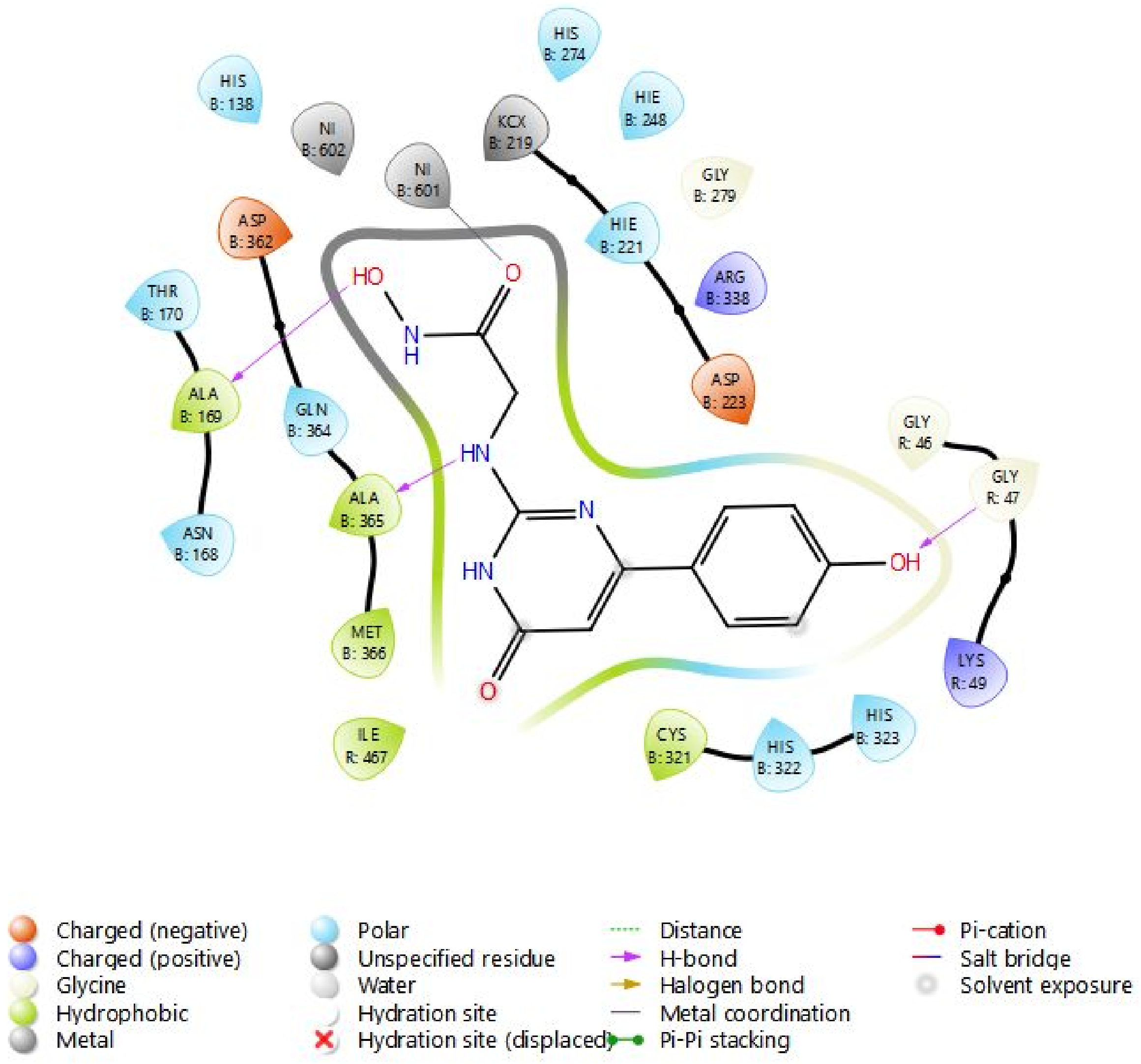

Figure S18. Interactions of compound 10 with amino acid residues at urease active site (PDB ID 6QSU)

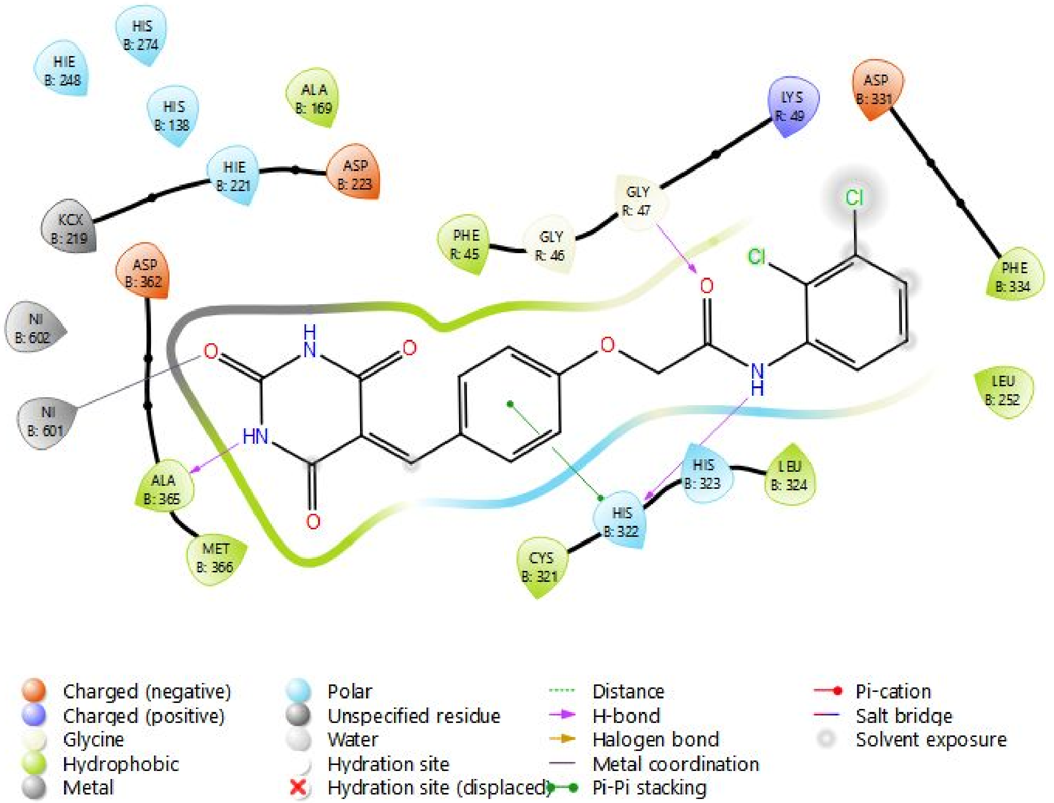

Figure S19. Interactions of compound 13 with amino acid residues at urease active site (PDB ID 6QSU)

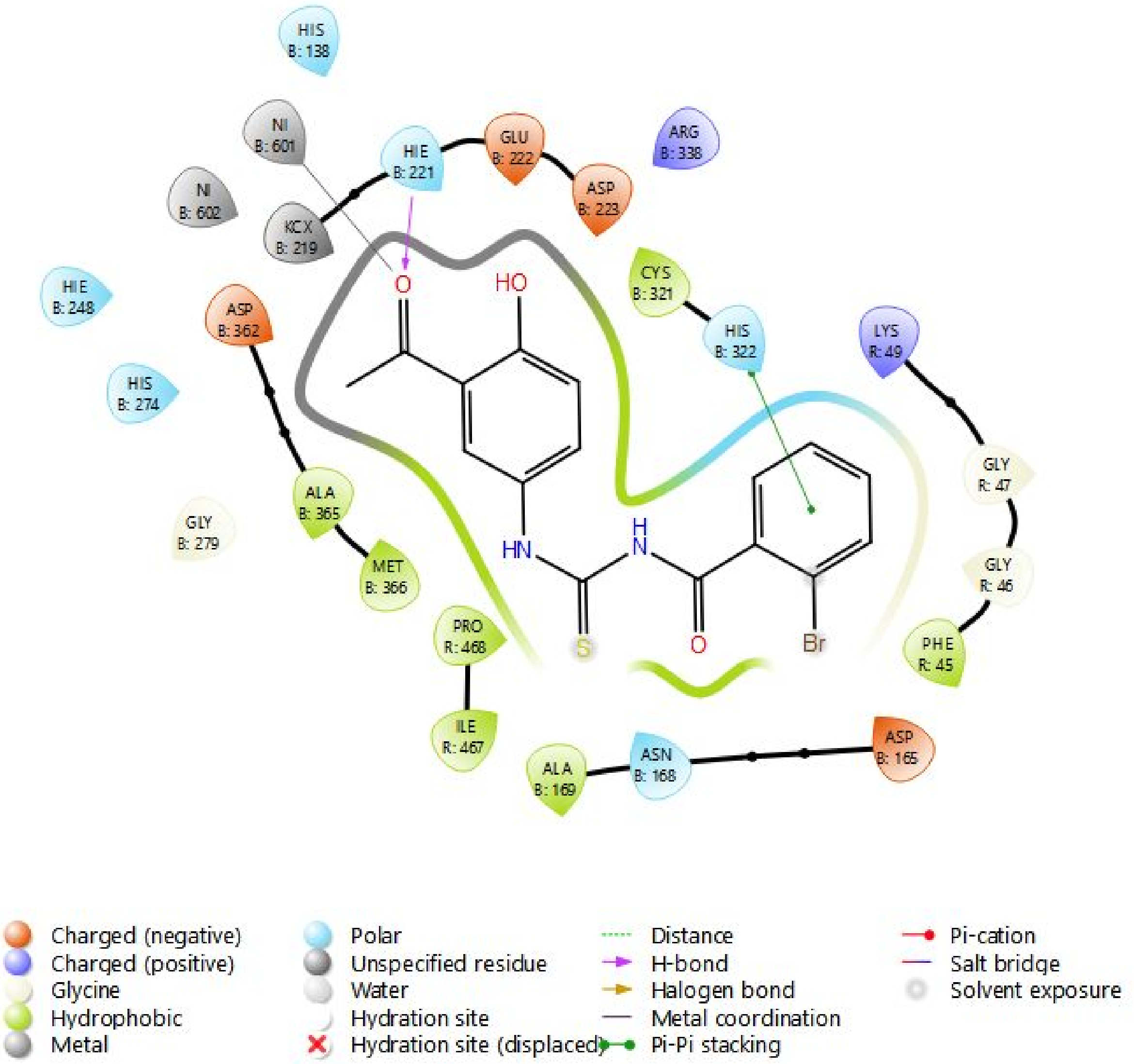

Figure S20. Interactions of compound 17 with amino acid residues at urease active site (PDB ID 6QSU)

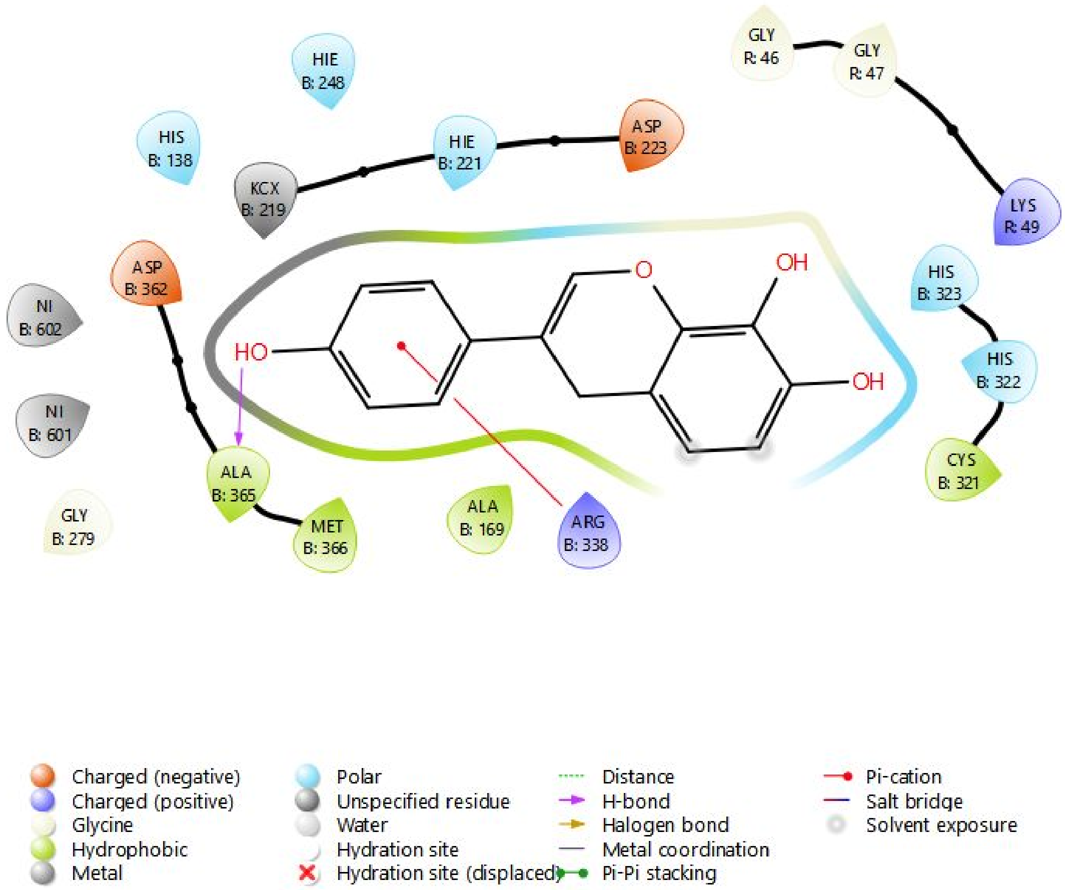

Figure S21. Interactions of compound 19 with amino acid residues at urease active site (PDB ID 6QSU)

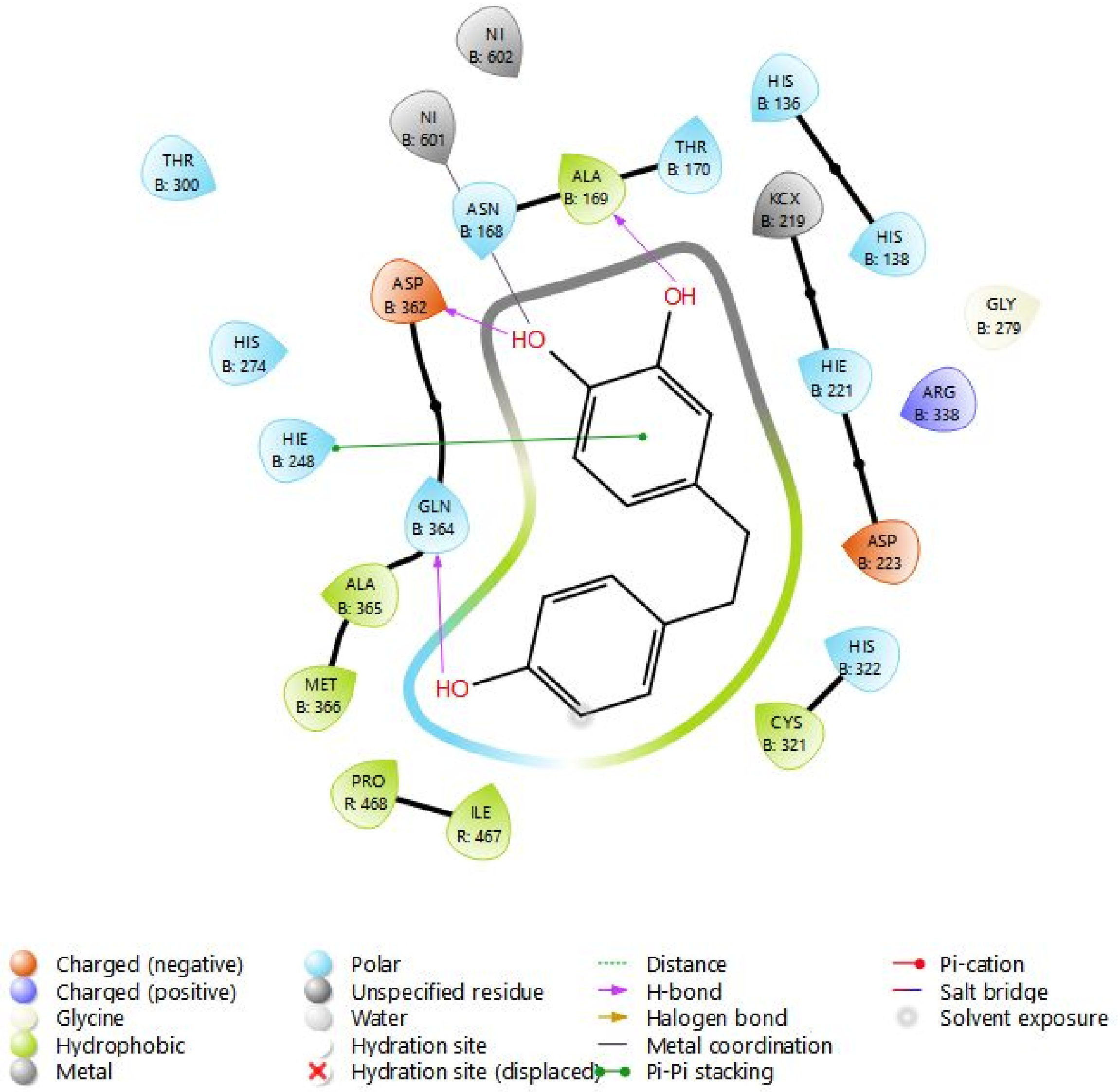

Figure S22. Interactions of compound 26 with amino acid residues at urease active site (PDB ID 6QSU)

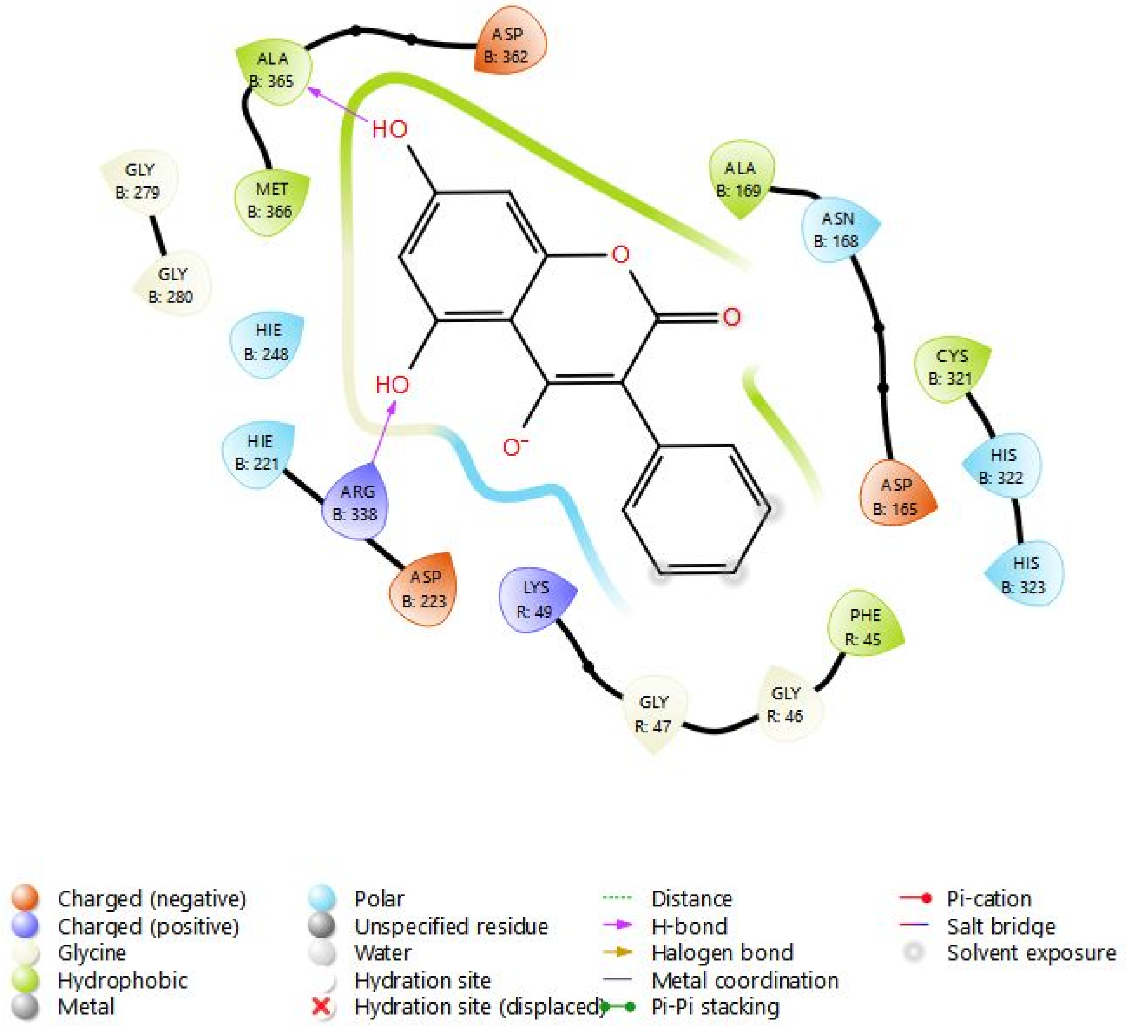

Figure S23. Interactions of compound 32 with amino acid residues at urease active site (PDB ID 6QSU)

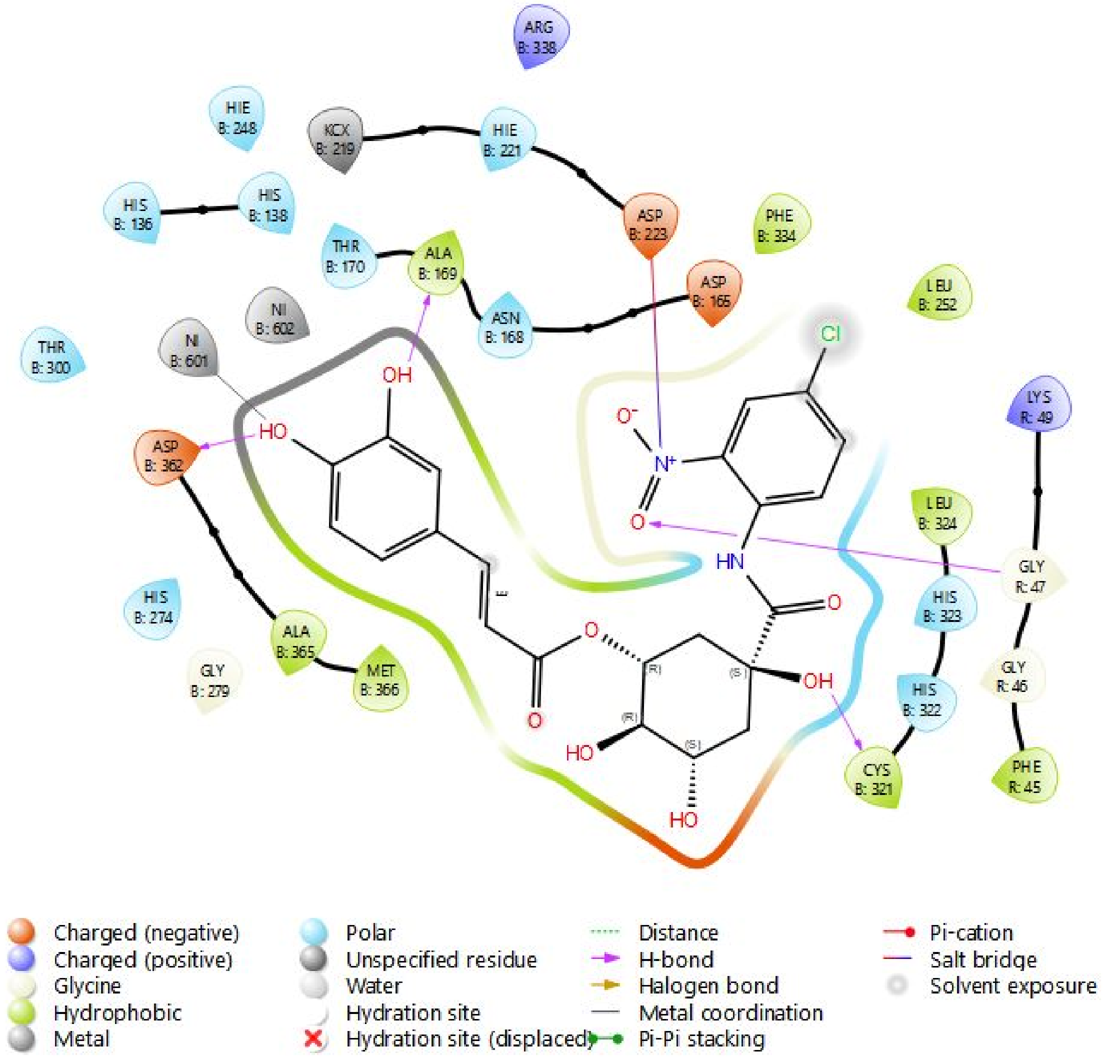

Figure S24. Interactions of compound 33 with amino acid residues at urease active site (PDB ID 6QSU)
